# Chalcogen substitution co-tunes photochromism and hydrogen bonding in semicarbazone photoswitches

**DOI:** 10.1039/d6sc03055f

**Published:** 2026-06-09

**Authors:** Bengi Sentürk, Siebe Lekanne Deprez, Westley Hennesen, Célia Fonseca Guerra, Fabian Eisenreich

**Affiliations:** a Department of Chemical Engineering and Chemistry, Institute for Complex Molecular Systems, Eindhoven University of Technology 5600 MB Eindhoven The Netherlands f.r.eisenreich@tue.nl; b Department of Chemistry and Pharmaceutical Sciences, AIMMS, Vrije Universiteit Amsterdam De Boelelaan 1108 Amsterdam 1081 HZ The Netherlands

## Abstract

Replacing even a single atom can profoundly alter the performance of photoswitches. Yet, using this strategy to co-tune light-responsiveness and supramolecular function in photoswitches remains unexplored. We synthesized two series of semicarbazone photoswitches, varying the C=X unit (X = O, S, Se) and the substituent on the imine moiety (phenyl *vs.* methoxy-pyridyl). UV-vis spectroscopy and DFT analysis reveal a red-shift in absorption towards the visible region as the π_HOMO_–π_LUMO_ gap narrows from O to Se. In parallel, heavier chalcogens increase the *E* → *Z* photoisomerization quantum yield. Beyond these optical effects, chalcogen substitution reshapes hydrogen-bonding pathways. In the phenyl series, it amplifies supramolecular self-association, yielding more stable π–π stacked, hydrogen-bonded aggregates. In the pyridyl series, it reinforces intramolecular hydrogen bonding, locking the sulfur and selenium analogue in the *Z*-isomer, whereas the oxygen derivative remains exclusively in the *E*-form. In mixtures of O-, S-, and Se-derivatives, we achieve wavelength-selective, stepwise deactivation of supramolecular aggregates, switching off the strongest associating species first. Overall, swapping a single chalcogen atom provides control over where these photoswitches absorb, how they isomerize, and how they self-associate. More broadly, this atom-level modification offers a strategy to modify both photophysics and supramolecular organization across carbonyl-containing photoswitch families.

## Abstract 2

Single-atom exchange reshapes how photoswitches interact with light and with each other. O/S/Se substitution in semicarbazones enables wavelength-selective, stepwise disassembly of mixed aggregates, linking atom-level design to programmable assembly.

## Graphical abstract

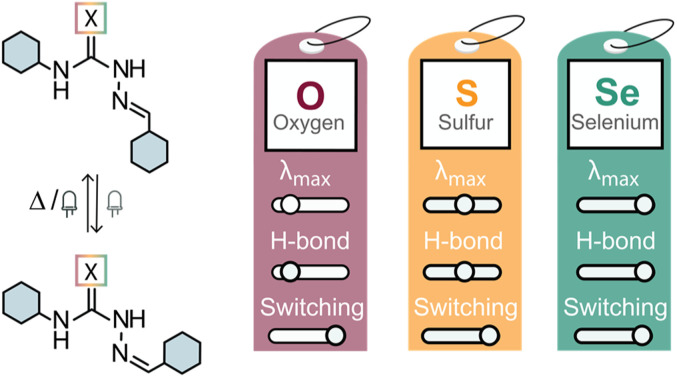

## Introduction

In diverse areas of chemistry, the replacement of a single atom within a functional group can reshape molecular properties. Chalcogen exchange (O → S/Se) stands out because it can simultaneously impact the electronic character of the group and its hydrogen-bonding interactions. In medicinal chemistry, O → S/Se exchange is widely used to tune biological activity and selectivity,^1–3^ exemplified by urea/thiourea pairs in which the sulfur analogue often shows enhanced hydrogen-bond donation.^4^ In materials science, replacing urea with thiourea moieties in densely hydrogen-bonded networks can induce a transition from semicrystalline to amorphous packing, with major consequences for mechanical robustness and self-healing.^5^ Thionation of carbonyls red-shifts absorption and enhances electron affinity, improving organic semiconductor performance.^6–8^ In supramolecular chemistry, amide → thioamide exchange in tricarboxamide-based monomers can reorganize directional hydrogen-bonding patterns, leading to more cooperative polymerization and, in chiral systems, enhanced supramolecular chirality.^9,10^ Fonseca Guerra and co-workers rationalized these effects computationally, emphasizing that the consequences of O → S/Se exchange are not captured by electronegativity trends alone, but often reflect atom size-driven changes in bonding and electronic structure.^11,12^ Collectively, these examples highlight atomic substitution as a powerful molecular design principle with consequences well beyond conventional substituent effects.

Photoswitches provide a particularly sensitive platform to probe this principle. These molecules undergo reversible structural changes enabling precise control over molecular properties in contexts ranging from adaptive materials^13^ and photopharmacology^14^ to supramolecular polymers.^15^ Because their electronic structure is highly responsive, even small modifications to the core or its substituents can strongly affect absorption spectra, isomerization barriers, and thermal stability.^16–20^ To translate these light-induced changes into supramolecular function, most designs append hydrogen-bonding motifs to photochromic scaffolds, such as azobenzenes,^21–30^ diarylethenes,^31,32^ and stiff-stilbenes.^33^ In our previous work,^34^ we took a different approach and introduced thiosemicarbazones as a new photoswitch class, in which the hydrogen-bonding motif is embedded directly into the switching core. In both appended and embedded designs, however, tuning is typically achieved by varying substituents around an otherwise fixed core. This raises the question of whether much subtler single-atom changes within the core can be used systematically to control not only photophysical properties but also supramolecular interactions.

Chalcogen substitution has been explored sporadically in photoswitch design and is used as a structural variant to adjust photophysics. For instance, hemithioindigos,^35^ a distinct photoswitch family, arise from an O → S substitution of hemiindigos,^36^ which shifts the lowest-energy absorption band to shorter wavelength, while remaining in the visible region.^37^ Thermal half-lives also change, without a consistent trend across derivatives.^37^ In alkoxy-substituted azobenzenes,^38^ chalcogen substitution (S → Se → Te) produces a pronounced bathochromic shift of the n → π* absorption (Δ*λ*_max_ ≈ 100 nm across the series); while tellurium substitution suppresses photoswitching under ambient conditions. Related heteroatom exchanges have been reported in a few other photochromes, such as fulgides,^39^ diarylethenes,^40,41^ and *N*-acylketoenamines.^42^ In contrast, carbonyl-containing photoswitches, such as hydrazones,^43,44^ acylhydrazones,^45^ donor–acceptor Stenhouse adducts,^46^ and thiosemicarbazones,^34^ are particularly well suited for systematic O → S/Se exchange at the carbonyl position, yet this approach has not been examined.

**Fig. 1:**
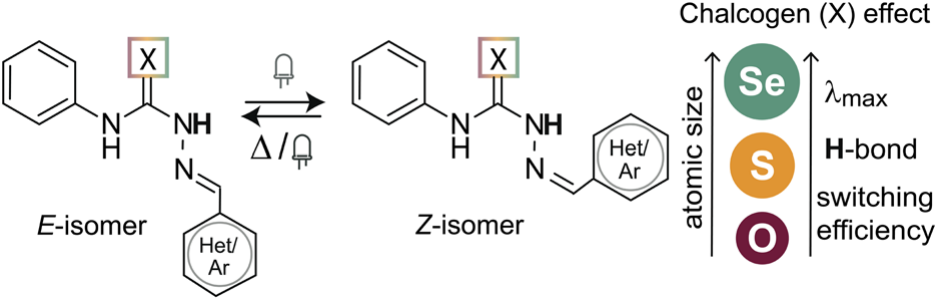
Systematic chalcogen substitution at the carbonyl group of semicarbazone photoswitches.

Here, semicarbazone photoswitches serve as a model platform to test whether targeted substitution at the carbonyl heteroatom can simultaneously tune photoswitch performance and supramolecular binding. While we introduced thiosemicarbazone photoswitches in our earlier report,^34^ the corresponding oxygen analogues remain comparatively underexplored,^47^ and selenosemicarbazone photoswitches are essentially unknown. In this work, we show that systematic O/S/Se exchange within the semicarbazone switching unit governs both photophysical and supramolecular properties (Fig. 1): it red-shifts absorption, alters PSS distributions and thermal half-lives, as well as strengthens intra- and intermolecular noncovalent interactions, while preserving clean isosbestic behavior and good fatigue resistance. These coupled changes enable wavelength-selective, stepwise deactivation of semicarbazone association in mixed systems. Although our study focuses on semicarbazones, we anticipate that analogous single-atom substitution strategies could be explored in other photoswitch platforms to tune photophysics and, when combined with suitable interaction sites, supramolecular behavior.

## Results and discussion

### Molecular design and synthesis

We designed two structurally coherent series of semicarbazone photoswitches to isolate the effect of chalcogen substitution (X = O, S, Se) on photochromic and supramolecular behavior. The two series were intentionally chosen to represent distinct interaction modes. In the first series, X-SC1, an unsubstituted phenyl ring was selected to eliminate additional electronic effects from the aromatic backbone, allowing a direct comparison of atomic substitution within the carbonyl group while favoring intermolecular association through noncovalent interactions. In the second series, X-SC2, a methoxy-substituted pyridyl group was introduced to promote six-membered intramolecular hydrogen bonding in the *Z*-isomer. This design choice was guided by our previous findings that such interactions can significantly stabilize the otherwise metastable *Z*-configuration in thiosemicarbazones.^34^ Comparing oxygen, sulfur, and selenium analogues across these two series allows us to examine how single-atom chalcogen substitution modulates two complementary design regimes: intermolecular aggregation in the X-SC1 series and intramolecular hydrogen-bond stabilization in the X-SC2 series, and how these effects influence isomer stability and photochromic response.

**Fig. 2:**
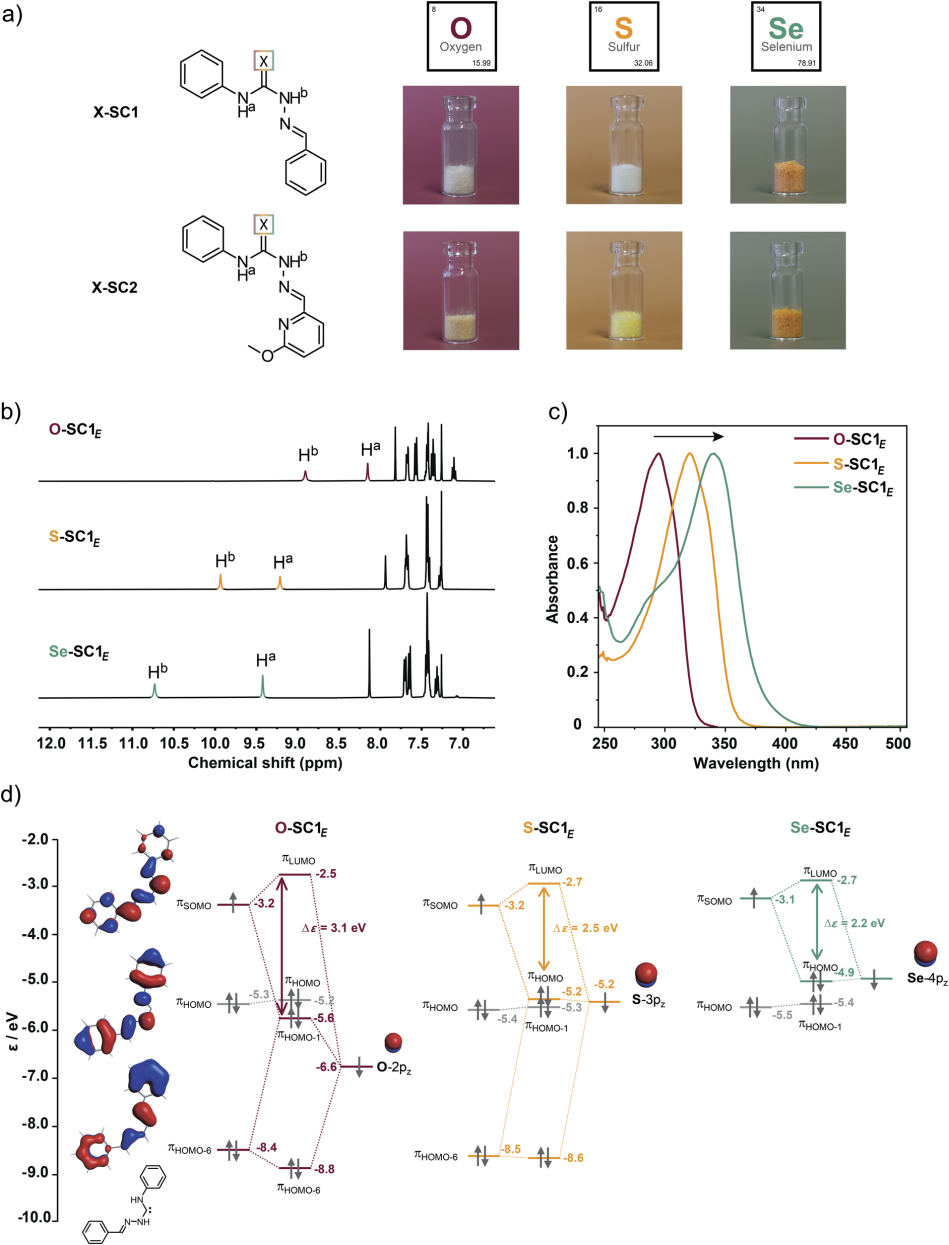
(a) Chemical structure and photographs of semicarbazone photoswitches X-SC1 and X-SC2 with chalcogen substitution (X = O, S, Se). (b) ^1^H NMR spectra of X-SC1*_E_* in CDCl_3_. (c) Normalized UV-vis absorption spectra of semicarbazone photoswitches X-SC1*_E_* in chloroform. (d) Orbital interaction diagram for the π bond electron-pair bond in X-SC1*_E_*, including the orbital energies (in eV) and isosurfaces (at ± 0.03 au), computed at a BLYP-D3(BJ)/TZ2P level of theory.

The target compounds share a common framework and were synthesized using two complementary routes. In the first approach, aryl hydrazones were obtained by condensing hydrazine monohydrate with the corresponding aldehydes in ethanol, then converted to the final photoswitches by reaction with iso-, isothio-, or isoselenocyanates. The isoselenocyanates were prepared separately from elemental selenium and anilines under mild conditions.^48^ In the second, more direct route, commercially available semicarbazide derivatives were coupled with the aldehydes under reflux in ethanol in the presence of trifluoroacetic acid, providing the desired products. Both strategies afforded the O-, S-, and Se-containing analogues in high yields (74–99%). All compounds were purified by precipitation or column chromatography and fully characterized by NMR and FT-IR spectroscopy, as well as MALDI-TOF mass spectrometry (Fig. S1–S18). Photographs of the isolated compounds (Fig. 2a) already hint at the underlying electronic differences: the selenium analogues appear orange, indicating a red-shifted absorption into the visible range.

### Ground-state electronic structure and absorption of the X-SC1 series

Having established access to all analogues, we next examined how chalcogen substitution affects their ground-state electronic environment. Analysis of the ^1^H NMR spectra in CDCl_3_ of the X-SC1*_E_* series revealed a systematic downfield shift of the two urea N–H resonances (H^a^ and H^b^) from O → Se (Fig. 2b). This trend indicates increasing acidity of both protons when descending the chalcogen group, consistent with stronger polarization of the N–H bonds and the electron-accepting character of the C=X unit for heavier chalcogens.^49,50^

UV-vis spectra of X-SC1*_E_* in chloroform reveal all three compounds absorb strongly in the near-UV through a π–π* transition with a clear chalcogen dependent trend: absorption maximum shifts from 294 nm (O-SC1*_E_*) to 321 nm (S-SC1*_E_*) and 340 nm (Se-SC1*_E_*, Fig. 2c). To rationalize these experimental observations at the molecular level, we examined the electronic structure of the X-SC1 series using DFT calculations. Conformational analysis identified the global minimum structures of the X-SC1 monomers (Fig. S37–S40). The experimental crystal structure previously reported for the thiosemicarbazone analogue (S-SC1*_E_*) was used as a structural reference,^34^ from which the oxygen and selenium analogues were generated by single-atom substitution at the C=X position prior to optimization. In agreement with experimental observations, the *E*-isomer is thermodynamically favored, while the *Z*-isomer lies higher in energy by 2.7–2.8 kcal mol^−1^ depending on the chalcogen atom. The *E*-isomers adopt a nearly planar geometry, and enforcing *C*_*s*_ symmetry introduces no energetic penalty. This planar structure enables a clear separation of *σ* and π electronic contributions and provides a suitable reference for analyzing the electronic transitions responsible for the optical response. The calculated π_HOMO_–π_LUMO_ gap decreases from 3.1 eV for O-SC1*_E_* to 2.5 eV for S-SC1*_E_* and 2.2 eV for Se-SC1*_E_*, which is consistent with our experimental UV-vis results. The orbital analysis further indicates that the HOMO/LUMO pair in X-SC1*_E_* arises from bonding and antibonding combinations of the chalcogen npz orbital with a molecular π orbital on the semicarbazone framework. As the C=X bond lengthens from O to Se, the reduced orbital overlap leads to weaker bonding–antibonding splitting and hence a smaller π_HOMO_–π_LUMO_ gap (Fig. 2d). This phenomenon aligns well with the study on aldehyde derivatives^11^ by Fonseca Guerra and co-workers. Thus, both experiment and theory demonstrate that exchanging a single atom at the C=X bond provides a direct means to tune the optical response of semicarbazone photoswitches.

### Absorption and photoisomerization behavior of semicarbazones

After establishing the chalcogen-dependent red-shift, we next investigated the photoisomerization behavior of these semicarbazones. In our previous study on thiosemicarbazone photoswitches,^34^ a solvent screening revealed that strongly polar or hydrogen-bonding solvents (*e.g.*, DMF, DMSO, alcohols) largely suppress the photoresponse due to stabilization of the *E*-isomer through solvent–solute hydrogen bonding, which promotes rapid thermal back-isomerization of the photo-generated *Z*-isomer. In contrast, less polar and weakly coordinating solvents allow efficient photoswitching and longer *Z*-isomer lifetimes. Chloroform was therefore chosen as a weakly polar, weakly coordinating solvent in which all members of both series are highly soluble while preserving the intermolecular interactions responsible for aggregation. In neat chloroform, however, the response to light proved inconsistent: in some cases, irradiation caused little spectral change (Fig. S19). We attribute this to acid-catalyzed *Z* → *E* back-isomerization,^51^ as residual acidity in chloroform can accelerate thermal relaxation and prevent accumulation of the *Z*-isomer. Both water saturation and neutralization with basic aluminium oxide restored reproducible photoisomerization behaviour and increased the observed thermal half-lives, consistent with suppression of acid-mediated back-relaxation pathways (Scheme S1). Because water saturation provided a simple and practical method for obtaining reproducible results without introducing additional chemical species, all subsequent spectroscopic measurements were therefore performed in water-saturated chloroform. More broadly, this potential sensitivity to residual acidity may not be unique to semicarbazones and could also affect other C=N photoswitches such as hydrazones^43–45^ and imines,^52^ although this has not yet been systematically investigated. We deliberately avoided adding base, because deprotonation of the semicarbazone N–H groups would substantially alter both the absorption properties and the hydrogen-bonding interactions we aim to probe.

**Fig. 3:**
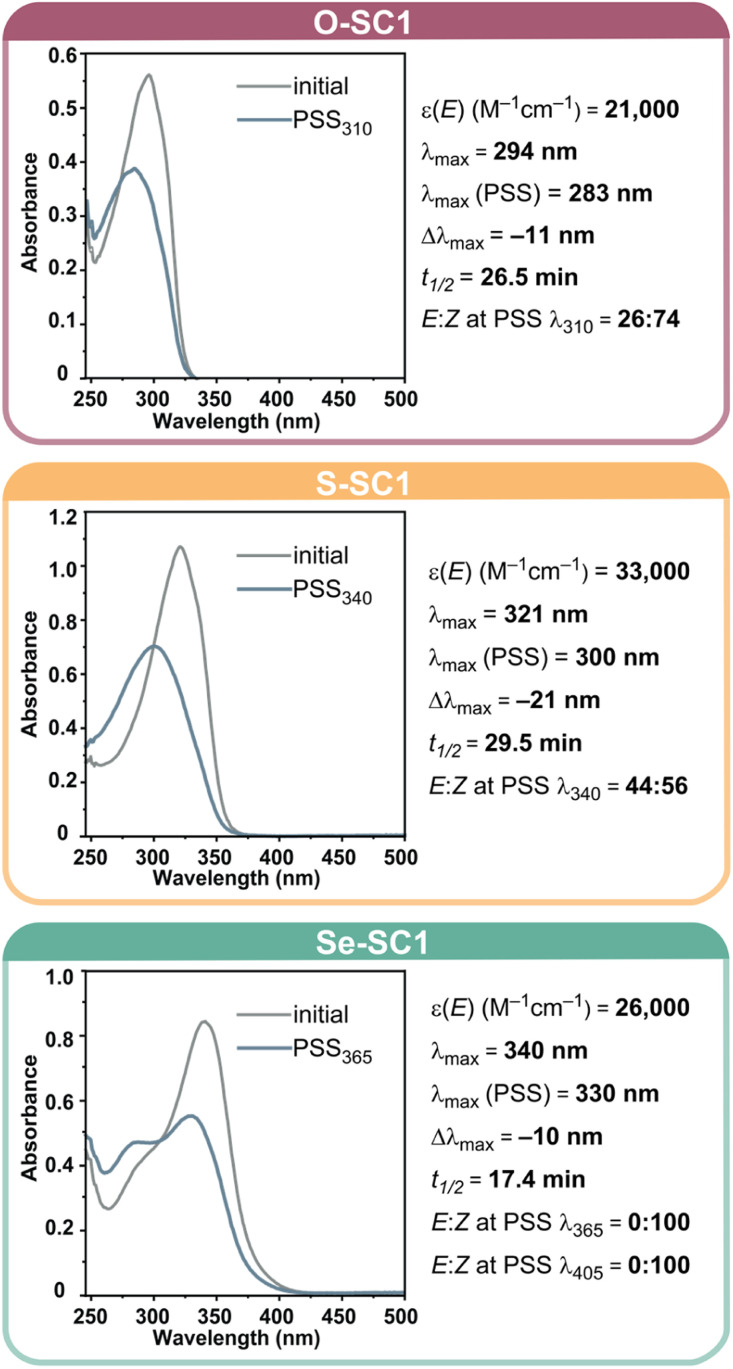
Photoisomerization of X-SC1*_E_* in water-saturated chloroform. Top: conversion of O-SC1*_E_* (gray line, *c* = 2.83 × 10^−5^ M) to O-SC1Z,PSS (blue line) with *λ*_irr_ = 310 nm. Middle: conversion of S-SC1*_E_* (gray line, *c* = 3.25 × 10^−5^ M) to S-SC1*_Z_*_,PSS_ (blue line) with *λ*_irr_ = 340 nm. Bottom: conversion of Se-SC1*_E_* (gray line, *c* = 3.41 × 10^−5^ M) to Se-SC1*_Z_*_,PSS_ (blue line) with *λ*_irr_ = 365 nm.

All three X-SC1 compounds exist exclusively as the thermodynamically favored *E*-isomer prior to irradiation (Fig. 3), as confirmed by ^1^H NMR spectroscopy in CDCl_3_ (Fig. S26–S28). Each derivative displays a high molar absorptivity (*ε*(*λ*_max_) = 21 000 for O-SC1; 33 000 for S-SC1; 26 000 M^−1^ cm^−1^ for Se-SC1, Table S2). For photoisomerization, the irradiation wavelength was chosen near the respective *λ*_max_ (310 nm for O-SC1, 340 nm for S-SC1, and 365 nm for Se-SC1). Excitation at these wavelengths induced efficient *E* → *Z* photoisomerization. Due to the pronounced red-shift of the selenium derivative, efficient photoisomerization of Se-SC1 could also be achieved upon irradiation at 405 nm (Fig. S22). O-SC1 and S-SC1 reached PSS distributions of 26 : 74 and 42 : 58 (*E* : *Z*), respectively, while Se-SC1 underwent quantitative conversion to the *Z*-isomer under both 365 and 405 nm irradiation (Fig. S26–S28). Additional concentration-dependent NMR experiments on O-SC1, S-SC1, and Se-SC1 showed that the PSS compositions remained unchanged over the investigated concentration range of ∼1–11 mM (Fig. S26–S27). The resulting metastable *Z*-isomers relaxed thermally on comparable timescales, with half-lives (*t*_1/2_) of 26.5 (O-SC1), 29.5 (S-SC1), and 17.4 min (Se-SC1, Fig. S20–S22). In all cases, the UV-vis traces displayed clean isosbestic points throughout isomerization, consistent with a two-state process. Overall, chalcogen substitution shifts *λ*_max_ and alters PSS composition, while leaving the thermal half-lives in the same range.

**Fig. 4:**
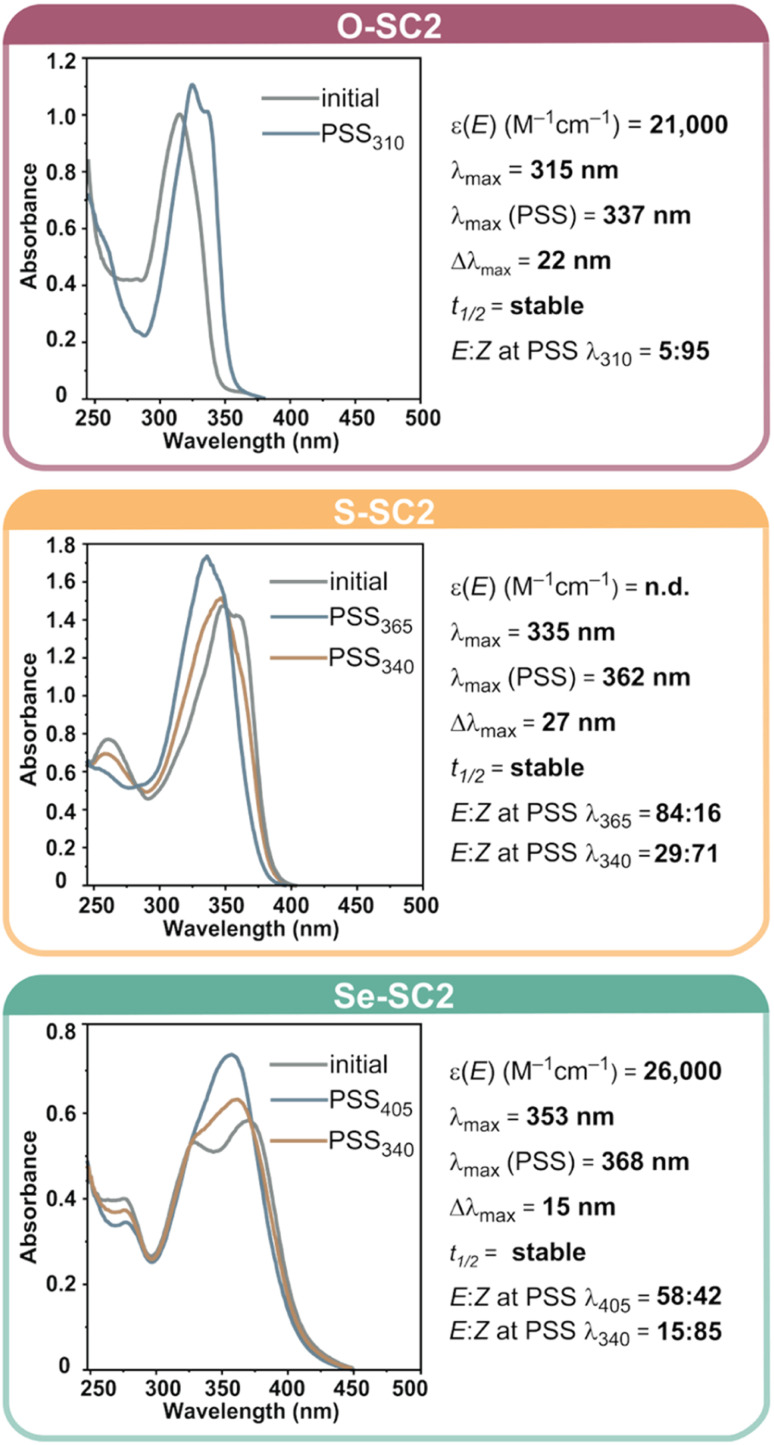
Photoisomerization of X-SC2 in water-saturated chloroform. Top: conversion of O-SC2*_E_* (gray line, *c* = 4.27 × 10^−5^ M) to O-SC2*_Z_*_,PSS_ (blue line) with *λ*_irr_ = 310 nm. Middle and bottom: for the P-type photoswitches S-SC2 (*c* = 4.31 × 10^−5^ M) and Se-SC2 (*c* = 3.61 × 10^−5^ M), irradiation at *λ*_irr_ = 365 nm and 405 nm, respectively, produced the first PSS (blue line), while subsequent irradiation at 340 nm afforded a second PSS (orange line). Photoswitches were labelled as thermally stable when no thermal *Z* → *E* isomerization was observed for at least 60 min.

Turning to the X-SC2 series (Fig. 4), a striking difference emerged when these compounds were dissolved in CDCl_3_. While O-SC2 exists exclusively as the *E*-isomer and displays a molar absorptivity of 21 000 M^−1^ cm^−1^ at *λ*_max_, S-SC2 adopts predominantly the *Z*-isomer (∼93%). Because S-SC2 could not be obtained as a single pure isomer either at thermal equilibrium or upon photoirradiation, its molar absorptivity could not be reliably determined. In contrast, Se-SC2 is observed quantitatively in the *Z*-form, as confirmed by ^1^H NMR spectroscopy in CDCl_3_, and exhibits a molar absorptivity of 26 000 M^−1^ cm^−1^ (Fig. S29–S31). The pronounced *Z*-preference of S-SC2 and Se-SC2 can be rationalized by the formation of an internal hydrogen bond (N–H^b^⋯N^pyr^). As the chalcogen becomes larger, the C=X bond lengthens and the π* orbital on the C=X fragment is stabilized, increasing its electron-accepting character in the π system. This enhances the interaction between the lone pair of the adjacent N–H^b^ group and the π* on C=X, rendering the N–H^b^ group more electron-deficient and hence a stronger hydrogen-bond donor. The resulting intramolecular hydrogen bonding therefore strengthens along the series.^11^ Upon irradiation at 310 nm, the O-analogue switched almost quantitatively to the *Z*-isomer (*E* : *Z* = 5 : 95, Fig. S23 and S29), but the process was irreversible, as the *E*-isomer could not be regenerated thermally or photochemically under these conditions. In contrast, both sulfur and selenium derivatives acted as P-type photoswitches. For S-SC2, irradiation at 365 nm enriched the *E*-isomer at PSS with *E* : *Z* = 84 : 16, while subsequent illumination with 340 nm regenerated the *Z*-isomer with *E* : *Z* = 29 : 71 (Fig. S24 and S30). Se-SC2 shows a similar behavior, reaching PSS values of *E* : *Z* = 58 : 42 upon 405 nm illumination and *E* : *Z* = 15 : 85 under 340 nm light (Fig. S25 and S31). Notably, no observable thermal back-relaxation was detected for either compound over 1 h at 20 °C. These results demonstrate P-type switching behaviour and show that chalcogen substitution controls not only absorption and PSS composition but also the reversibility of switching.

We next assessed fatigue resistance by subjecting the compounds to repeated switching cycles (Fig. S32 and S33). All three T-types photoswitches (O-SC1, S-SC1, and Se-SC1) exhibited reproducible changes in absorbance over multiple on–off cycles, indicating negligible photofatigue. Similarly, the P-type photoswitches S-SC2 and Se-SC2 could be cycled at their respective excitation wavelengths without significant loss of amplitude. To further quantify the influence of chalcogen substitution on the primary photochemical event, we determined the quantum yields of the X-SC1 and X-SC2 series (Table S3). For the X-SC1 series, the oxygen analogue showed a modest *E* → *Z* quantum yield of 0.19, which increased to 0.33 for the sulfur analogue and reached 0.44 for the selenium analogue. Similarly, O-SC2 (*E* → *Z*) and Se-SC2 (*Z* → *E*) displayed quantum yields of 0.20 and 0.45, respectively. Determination of the quantum yield for S-SC2 was not pursued because accurate isomer-specific molar absorptivity values could not be obtained. Overall, chalcogen substitution preserves robust photoswitching over multiple cycles while enhancing the intrinsic photochemical efficiency of the isomerization.

### Chalcogen-dependent aggregation in the X-SC1 series

**Fig. 5:**
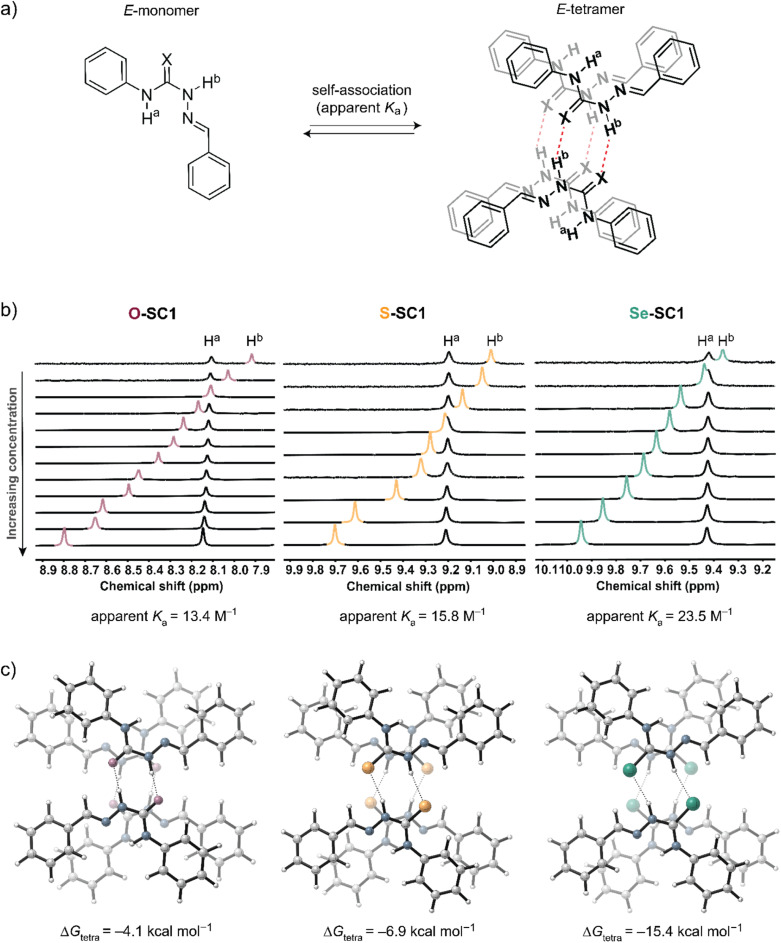
(a) Self-association scheme of semicarbazones. (b) ^1^H NMR spectra (400 MHz, 25 °C, CDCl_3_) of X-SC1*_E_* solutions with varying concentrations. (c) Gibbs free energies of the complexation affinity Δ*G*_tetra_ = *G*_tetramer_ − 4*G*_monomer_, computed at COSMO(chloroform)-ZORA-BLYP-D3(BJ)/TZ2P.

To assess how chalcogen substitution influences supramolecular behavior, we first monitored concentration-dependent changes in the ^1^H NMR spectra of the X-SC1 series. The urea N–H^b^ resonance shifted downfield with increasing concentration for all three derivatives, consistent with self-association *via* intermolecular hydrogen bonding (Fig. 5).^34,53^ Fitting the chemical-shift changes to a monomer–dimer equilibrium, used here as an effective description for self-association, provided apparent association constants *K*_a_ of 13.4 M^−1^ for O-SC1, 15.8 M^−1^ for S-SC1, and 23.5 M^−1^ for Se-SC1, indicating that the propensity for self-association increases with chalcogen size.

To clarify the nature of the associated species, we turned to DFT calculations (Fig. S40). Among the possible dimers, only a stacked motif was found to be thermodynamically accessible, whereas planar and chain-like hydrogen-bonded arrangements are disfavored. More importantly, the calculations reveal that a stacked tetramer [X-SC1*_E_*]_4_ is substantially more stable than the corresponding dimers, with Δ*G*_tetra_ values of −4.1, −6.9, and −15.4 kcal mol^−1^ for X = O, S, and Se, respectively. Thus, although the NMR data were fitted using a monomer–dimer model, the calculations indicate that the dominant aggregated species is a hydrogen-bonded, π–π stacked tetramer rather than a simple dimer, and its stability follows the same O < S < Se trend inferred from the NMR analysis.

Energy-decomposition analyses of the tetramers indicate that aggregation is cooperatively stabilized by N–H⋯X=C hydrogen bonding and π–π stacking between the aromatic rings. In [O-SC1*_E_*]_4_, hydrogen bonding and π–π interaction contribute with comparable weight, whereas for [S-SC1*_E_*]_4_ and [Se-SC1*_E_*]_4_ π–π stacking becomes increasingly dominant and the hydrogen-bonding contribution decreases slightly (Tables S5 and S6). This shift correlates with stronger dispersion interactions for the heavier chalcogens, which likely contribute to the overall increase in tetramer stability from O to S to Se. Together, experiment and theory show that exchanging a single chalcogen atom does not simply strengthen a single hydrogen bond, but reorganizes the balance between hydrogen bonding and π–π stacking, yielding progressively more stable π–π stacked tetramers from O to Se. We further computed ^1^H NMR chemical shifts for monomers, dimers, and tetramers (Fig. S41, Tables S9 and S10). The calculated shifts reproduce the experimental trend and show pronounced deshielding of the N–H^b^ proton upon aggregation consistent with hydrogen bonding between N–H^b^ and the adjacent C=X group.

### Wavelength-selective stepwise deactivation of aggregates in the X-SC1 series

**Fig. 6:**
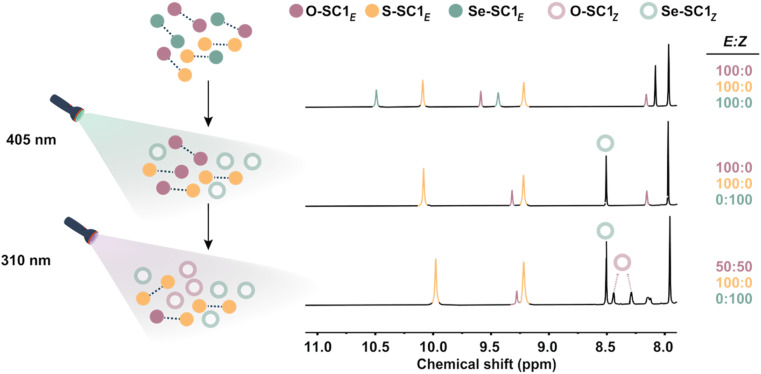
Wavelength-selective *E* → *Z* photoisomerization in a mixture of X-SC1 (7 mM O-SC1, 14 mM S-SC1, 10 mM Se-SC1). Cartoons (left) and ^1^H NMR spectra (right, N–H region, CDCl_3_, 25 °C) of the mixture before irradiation, after irradiation at 405 nm and after subsequent 310 nm irradiation, showing sequential conversion of Se-SC1 and O-SC1 from *E* to *Z* in the presence of S-SC1 (colours as indicated in the legend).

We next tested whether the chalcogen-tuned spectral separation can be harnessed for wavelength-programmed, stepwise shutdown of supramolecular association in solution. For this purpose, a mixture of O-SC1*_E_*, S-SC1*_E_*, and Se-SC1*_E_* in CDCl_3_ (7 mM, 14 mM, and 10 mM, respectively) was irradiated at wavelengths that address the individual photoswitches selectively (Fig. 6, and S36). The concentrations were adjusted so that the N–H signals of the three components appear with comparable yet distinct intensities in the ^1^H NMR spectra, facilitating the assignment of each resonance to a specific photoswitch. Although Se-SC1 was primarily characterized using 365 nm irradiation, its red-shifted absorption profile extends sufficiently into the visible region to enable selective photoisomerization at 405 nm within the multicomponent mixture. Irradiation at 405 nm therefore selectively and quantitatively converted Se-SC1*_E_* into Se-SC1*_Z_*. Because steric constraints prevent the *Z*-isomer from engaging in productive supramolecular interactions, it is shifted towards a more weakly associated or monomeric state.^34^ Subsequent irradiation at 310 nm then induced the *E* → *Z* isomerization of O-SC1, further decreasing the population of O-based aggregates, whereas S-SC1 remains in the *E*-form and continues to associate. Notably, the extent of O-SC1 photoisomerization observed in the mixture differs from the PSS value determined for the isolated photoswitch (Fig. 3), which may arise from intermolecular interactions and competition for photon absorption within the multicomponent system. Interestingly, after each illumination step, the N–H signals of the non-targeted photoswitches shifted slightly, pointing at weak co-aggregation that diminishes upon isomerization. These results demonstrate wavelength-selective, sequential deactivation of supramolecular association by targeted photoisomerization.

## Conclusions

In this study, we have shown that exchanging a single chalcogen atom in the C=X unit of semicarbazone photoswitches provides a simple way to tune both their photochemical response and their intra- and intermolecular noncovalent interactions. Across two closely related series, O → S → Se substitution red-shifts the lowest-energy absorption band from 283 to 340 nm (Δ*λ*_max_ = 57 nm) and increases the *E* → *Z* photoisomerization quantum yield from 19% to 44% (≈130% relative increase). In the phenyl series, the same substitution pattern strengthens supramolecular self-association, as reflected in increasing apparent association constants and progressively more stable π–π stacked, hydrogen-bonded tetramers from O to S to Se. In the pyridyl derivatives, it instead intensifies intramolecular hydrogen bonding and inverts the relative stability of the *E*- and *Z*-isomers, making it possible to select the desired starting isomer by design. Thus, a single atomic position governs both light responsiveness and the strength and pattern of hydrogen bonding. Combinations of O-, S-, and Se-based semicarbazones therefore display an intrinsic ordering in how strongly they aggregate and at which wavelengths they can be addressed. We exploited this hierarchy by using different colors of light to reduce the contribution of Se- and O-based aggregates in a stepwise manner, while S-based aggregates remained present in solution.

In principle, the same concept could be extended to more complex settings. For example, incorporating semicarbazones into polymer backbones, side chains, or cross-linkers could allow different segments to respond at different wavelengths and on different time scales, enabling controlled changes in association and, ultimately, material properties. Semicarbazones are also known as organocatalysts^53^ and biologically active reagents,^54^ suggesting that wavelength-selective photoisomerization could offer an additional handle to tune function in these contexts. More broadly, our findings indicate that single-atom chalcogen substitution can serve as a practical design parameter for multifunctional photochromes. Other photoswitch families that contain a carbonyl unit (*e.g.*, hydrazones, acylhydrazones, DASAs) may also be amenable to related tuning strategies. Such an approach could support the rational design of complex molecular assemblies, in which wavelength selectivity and interaction strength are tuned in tandem.

## Author contributions

B. S. conducted the syntheses, performed UV-vis and NMR spectroscopy experiments, as well as analyzed and interpreted the data. S. L. D. contributed to investigation and methodology and performed formal analysis and validation. W. H. contributed to investigation and validation. B. S. and F. E. conceived the idea, designed the study, and wrote the manuscript. C. F. G. and F. E. supervised the project and acquired funding. All authors discussed the results, edited the manuscript, and approved the final version of the manuscript.

## Conflicts of interest

There are no conflicts to declare.

## Supplementary Material

SC-017-D6SC03055F-s001

## Data Availability

All other data supporting the findings of this study, including experimental procedures and compound characterization, NMR spectroscopy, UV-vis spectroscopy and computational investigations are available within the article and its supplementary information (SI). All data are available from the corresponding author upon request. Supplementary information is available. See DOI: https://doi.org/10.1039/d6sc03055f.
