# Supplementary material for: Chalcogen substitution co-tunes photochromism and hydrogen bonding in semicarbazone photoswitches
Source: Chem Sci. 2026 Jun 9;17(29):14185–93. doi: 10.1039/d6sc03055f (PMC13271062; doi:10.1039/d6sc03055f)
Supplement: SC-017-D6SC03055F-s001 [file SC-017-D6SC03055F-s001.pdf]

Supplementary Information (SI) for Chemical Science.
This journal is © The Royal Society of Chemistry 2026

Supporting Information

Chalcogen Substitution Co-Tunes Photochromism and Hydrogen Bonding in Semicarbazone Photoswitches

Bengi Sentürk,[a] Siebe Lekanne Deprez,[b] Westley Hennesen,[a] Célia Fonseca Guerra,[b] and Fabian Eisenreich*[a]

[a] Department of Chemical Engineering and Chemistry, Institute for Complex Molecular Systems, Eindhoven University of Technology, 5600 MB Eindhoven, the Netherlands

[b] Department of Chemistry and Pharmaceutical Sciences, Amsterdam Institute for Molecular and Life Sciences (AIMMS), Vrije Universiteit Amsterdam, De Boelelaan 1108, Amsterdam 1081 HZ, the Netherlands.

Correspondence should be addressed to F. E. (E-mail: f.r.eisenreich@tue.nl)

Table of Contents

1. Methods and materials 3
2. Synthesis 5
2.1 ^{1}H and ^{13}C NMR Spectra 10
3. Photophysical and Photochemical Properties 19
3.1. Influence of residual acidity on photoisomerization behaviour 19
3.2. Photoswitching Studies 22
3.2.1. O-SC1 22
3.2.2. S-SC1 23
3.2.3. Se-SC1 24
3.2.4. O-SC2 25
3.2.5. S-SC2 25
3.2.6. Se-SC2 25
3.3. Photostationary States Determination 26
3.4. Fatigue Resistance 31
3.5. Quantum Yields 32
3.6. Self-association studies of the X-SC1 series 36
3.7. Stepwise Aggregate Deactivation Studies on X-SC1 39
4. Computational Investigations 40
4.1 Conformer computations 40
4.2 Excitations and time-dependent density functional theory (TD-DFT) computations 41
4.3 Bond energy analysis 44
4.4 Tetramer bonding analysis 46
4.5 Semicarbazone fragmentation 49
4.6 ^{1}H NMR trends for X-SC1 50
5.1 References 54
Cartesian Coordinates 58

1. Methods and materials

All reagents and solvents were purchased from commercial suppliers and used without further purification, unless specified. All The compound was synthesized, dried under high-vacuum, and then stored at room temperature.

NMR Spectroscopy. NMR spectra were recorded at 25 °C using a Bruker UltraShield 400 MHz spectrometer automatically tuned and matched to the correct operating frequencies. ^{1}H and ^{13}C NMR spectra were referenced to the residual solvent peak. Signals are reported in terms of chemical shift (ppm) and coupling constants (Hz). Abbreviations for multiplicity are as follows: s, singlet; d, doublet; t, triplet; m, multiplet; br, broad; hept, heptet.

MALDI-TOF Mass spectroscopy. Matrix assisted laser absorption/ionization mass time of flight (MALDI-TOF) measurements were performed on a Bruker Autoflex Speed using α-cyano-4-hydroxycinnamic acid (CHCA) and *trans*-2-[3-(4-*tert*-butylphenyl)-2-methyl-2-propenylidene]malononitrile (DCBT) as matrices. The compounds were dissolved in DCM in a concentration of 1 mg mL^{-1}.

Fourier transform infrared spectroscopy (FT-IR). FT-IR spectra were recorded on NICOLET iS20 (thermo scientific) as an average of 8 scans over an energy range of 450–4000 cm^{-1}.

UV-vis absorption spectroscopy. Spectra were recorded on a Agilent Cary 60 spectrophotometer, equipped with a Peltier temperature controller, which also controls the stirring. The irradiation beam was aligned orthogonally to the measurement beam of the spectrophotometer to enable simultaneous irradiation and probing of a 3.5 mL quartz cuvette (10 x 10 mm) in a thermostated cell holder with a magnetic stir bar at 20 °C. LEDs of various wavelengths (see **Table S1**) were employed to induce photoisomerization. These fiber-coupled LEDs were connected to the instrument with fiber optic cables supplied by ThorLabs (solarization resistant, SMA, 400 µm, 0.22 NA). The LEDs were operated at a constant current mode controlled by a ThorLabs DC4100 LED driver.

Table S1. Listing of the models of ThorLabs fiber-coupled LEDs utilized in this study, along with nominal wavelength and bandwidth measured as full width at half maximum (FWHM).

LED model number (ThorLabs)	Nominal Wavelength (nm)	Bandwidth/FWHM (nm)
M310F1	308	30
M340F4	340	10
M365FP1	365	9
M405FP1	405	12

2. Synthesis

Phenyl Isoselenocyanate (Se-1)

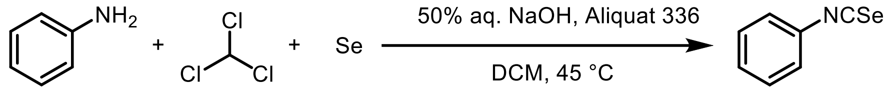

Following the procedure of Zakrzewski *et al.*,[1] a mixture of aniline (1.40 mL, 15.33 mmol, 1.0 equiv), chloroform (1,6 mL, 19,93 mmol, 1.3 equiv), DCM (10 mL), Aliquat 336 (0.2 g, 0.5 mmol), and 50% aqueous NaOH (4.5 mL ,86 mmol, 5.7 equiv) was stirred vigorously. An exotherm was observed. After the exotherm subsided, the biphasic mixture was heated at gentle reflux (~40 °C) until complete consumption of aniline as indicated by TLC (cyclohexane/ethyl acetate 9:1). The mixture was cooled to room temperature and selenium powder (1.5 g, 19 mmol, 1.2 equiv) was added. The reaction was maintained at ~40 °C for 1.5 h, with progress monitored by TLC. The mixture was cooled, diluted with water (15 mL) and DCM (15 mL), and filtered under reduced pressure through a Büchner funnel to remove unreacted selenium. The organic phase was dried over anhydrous $MgSO_4$, filtered, and concentrated under reduced pressure. The residue was adsorbed onto silica gel (10 g) and dry-loaded for column chromatography. Purification (cyclohexane/ethyl acetate gradient) afforded the product as a brown liquid (1.55 g, 8.51 mmol, 56%). ^1H NMR (400 MHz, $CDCl_3$, 25 °C) δ = 7.39 – 7.22 (m, 5H) ppm. ^{13}C NMR (100 MHz, $CDCl_3$, 25 °C) δ = 129.30, 127.84, 125.82 ppm (two carbon resonances are not clearly detected in the ^{13}C NMR, presumably because of weak intensity and/or overlap).

Benzaldehyde Hydrazone (H-1)[2]

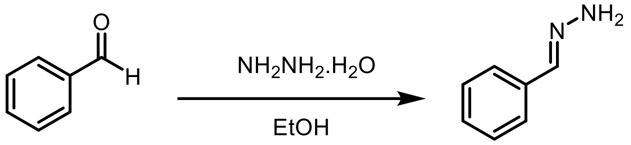

Hydrazine monohydrate (15.0 mL, 312 mmol, 3.1 equiv) was diluted with ethanol (50 mL) in a round-bottom flask. A solution of benzaldehyde (10.2 mL, 100.0 mmol, 1.0 equiv) in ethanol (30 mL) was added dropwise at room temperature via an addition funnel. After the addition was complete, the mixture was stirred for additional 2 h at room temperature. The reaction was diluted with water (20 mL) and DCM (20 mL), and the layers were separated. The aqueous phase was extracted with DCM (3 × 20 mL). The combined organic extracts were dried over anhydrous Na_2SO_4, filtered, and concentrated under reduced pressure to afford benzaldehyde hydrazone as a yellow liquid (11.86 g, 98.68 mmol, 99%). ^1H NMR (400 MHz, DMSO-d_6,

25 °C) δ = 7.72 (s, 1H), 7.51 – 7.45 (m, 2H), 7.34 – 7.28 (m, 2H), 7.24 – 7.20 (m, 1H), 6.80 (s, 2H) ppm.^{13}C NMR (100 MHz, DMSO-d_6, 25 °C) δ =138.41, 136.42, 128.48, 127.38, 125.17 ppm.

IR: υ_{max} (cm^{-1}) = 3336 (N–H), 1590 (C=N).

6-Methoxy-2-pyridinecarboxaldehyde hydrazone (H-2)[2]

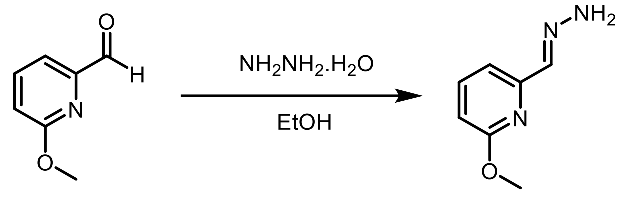

Hydrazine monohydrate (10.9 mL, 224 mmol, 3.1 equiv) was diluted with ethanol (50 mL) in a round-bottom flask. A solution of 6-methoxy-2-pyridinecarboxaldehyde (8.7 mL, 72.3 mmol, 1.0 equiv) in ethanol (30 mL) was added dropwise at room temperature via an addition funnel. After the addition was complete, the mixture was stirred for additional 2 h at room temperature. The reaction was diluted with water (20 mL) and DCM (20 mL), and the layers were separated. The aqueous phase was extracted with DCM (3 × 20 mL). The combined organic extracts were dried over anhydrous Na_2SO_4, filtered, and concentrated under reduced pressure to afford 6-methoxy-2-pyridinecarboxaldehyde hydrazone as a yellow liquid (10.52 g, 69.2 mmol mmol, 96%). ^{1}H NMR (400 MHz, $CDCl_3$, 25 °C) δ = 7.76 (s, 1H), 7.47 (t, J = 7.9, 1H), 7.26 (dd, J = 7.4, 1.9 Hz, 1H), 6.58 (dd, J = 8.2, 1.9 Hz, 1H), 5.84 (s, 2H), 3.88 (s, 3H) ppm. ^{13}C NMR (100 MHz, $CDCl_3$, 25 °C) δ =163.73, 151.89, 142.56, 138.71, 112.62, 109.64, 53.24 ppm.

(2*E*)-*N*-phenyl-2-(phenylmethylene)hydrazinecarboxamide (O-SC1)

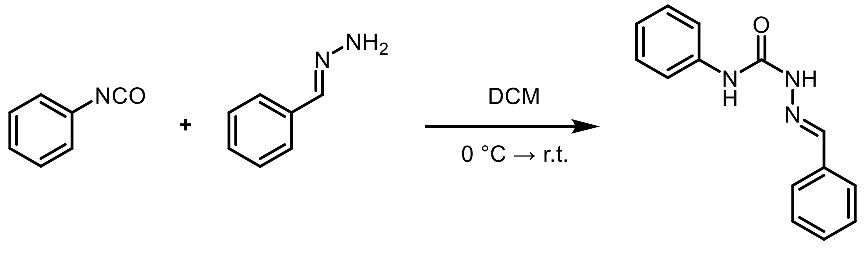

Benzaldehyde hydrazone (0.50 mL, 4.58 mmol, 1.0 equiv) was dissolved in anhydrous DCM (10 mL) and cooled to 0 °C in an ice bath. Phenyl isocyanate (555.0 mg, 4.60 mmol, 1.0 equiv) was added dropwise to the stirred solution. After the addition was complete, the mixture was stirred at room temperature for 1.5 h. The resulting precipitate was collected by vacuum filtration, washed with cold DCM, and dried under reduced pressure at 50 °C to give **O-SC1** as

a white powder (1.08 g, 4.52 mmol, 99%). ^{1}H NMR (400 MHz, DMSO-d_6, 25 °C) δ = 10.74 (s, 1H), 8.88 (s, 1H), 7.95 (s, 1H), 7.86 – 7.80 (m, 2H), 7.69 – 7.62 (m, 2H), 7.47 – 7.35 (m, 3H), 7.29 (dd, *J* = 8.5, 7.3 Hz, 2H), 7.02 (t, *J* = 7.4 Hz, 1H) ppm. ^{13}C NMR (100 MHz, DMSO-d_6, 25 °C) δ = 154.06, 141.25, 139.53, 134.27, 129.88, 129.07, 128.90, 127.12, 122.95, 120.37 ppm. IR: υ_{max} (cm^{-1}) = 3368, 3082 (N–H), 1679 (C=O), 1593 (C=N). MALDI-TOF MS: m/z calculated: 239.09 ([M+H]$^+$); found: 239.09.

(2*E*)-*N*-phenyl-2-(phenylmethylene)hydrazinecarbothioamide (S-SC1)

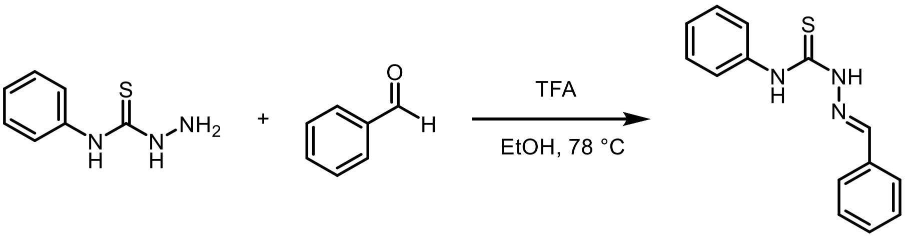

Compound was prepared according to our previous report.[3] A suspension of benzaldehyde (131.8 mg, 1.24 mmol, 1.0 equiv) and 4-phenylthiosemicarbazide (202.1 mg, 1.21 mmol, 1.0 equiv) in ethanol (10 mL) containing two drops of TFA was heated to reflux until a clear solution formed. The mixture was then cooled to room temperature and stirred for 2 h. The resulting solid was collected by filtration, washed with ethanol, and dried under reduced pressure at 50 °C to afford **S-SC1** as a white powder (262.9 mg, 1.03 mmol, 85%). ^{1}H NMR (400 MHz, DMSO-d_6, 25 ºC) δ = 11.83 (s, 1H), 10.11 (s, 1H), 8.17 (s, 1H), 7.97–7.84 (m, 2H), 7.61–7.54 (m, 2H), 7.46–7.41 (m, 3H), 7.41–7.34 (m, 2H), 7.25–7.18 (m, 1H) ppm. ^{13}C NMR (100 MHz, DMSO-d_6, 25 ºC) δ = 176.04, 142.89, 139.08, 134.02, 130.04, 128.64, 128.05, 127.63, 125.90, 125.33 ppm. IR**:** υ_{max} (cm^{-1}) = 3301, 3157 (N–H), 1505 (C=N), 1197 (C=S). MALDI-TOF MS: m/z calculated: 256.07 ([M+H]$^+$); found: 256.07.

(2*E*)-*N*-phenyl-2-(phenylmethylene)hydrazinecarboselenoamide (Se-SC1)

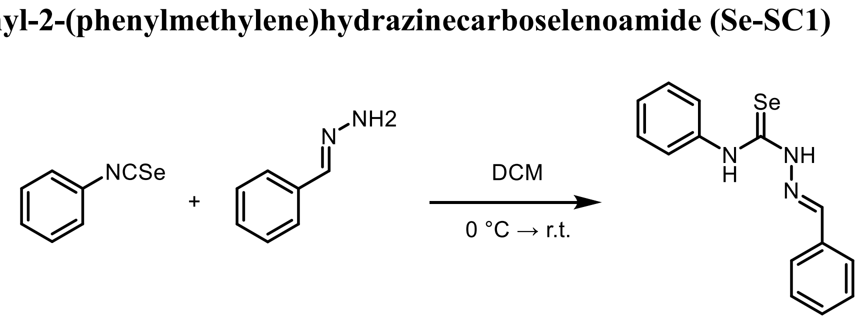

A solution of benzaldehyde hydrazone (224.8 mg, 1.87 mmol, 1.0 equiv) in anhydrous DCM (20 mL) was cooled to 0 °C in an ice bath. Compound **1.Se** (340.3 mg, 1.87 mmol, 1.0 equiv) was added dropwise. After the addition was complete, the mixture was stirred at room

temperature for 1.5 h. The resulting solid was collected by vacuum filtration, washed with cold DCM, and dried under reduced pressure at 50 °C to afford as orange crystals (429.7 mg, 1.42 mmol, 76%). ^{1}H NMR (400 MHz, DMSO-d_6, 25 °C) δ = 12.13 (s, 1H), 10.48 (s, 1H), 8.29 (s, 1H), 7.98 – 7.89 (m, 2H), 7.55 – 7.49 (m, 2H), 7.45 – 7.41 (m, 3H), 7.41 – 7.34 (m, 2H), 7.34 – 7.22 (m, 1H) ppm. ^{13}C NMR (100 MHz, DMSO, 25 °C) δ = 174.32, 144.23, 139.83, 133.89, 130.24, 128.65, 128.01, 127.79, 126.77, 125.88 ppm. IR: υ_{max} (cm^{-1}) = 3275, 3129 (N–H), 1500 (C=N), 753 (C=Se). MALDI-TOF MS: m/z calculated: 304.02 ([M+H]$^+$); found: 304.02.

(*E*)-2-((6-methoxypyridin-2-yl)methylene)-*N*-phenylhydrazine-1-carboxamide (O-SC2)

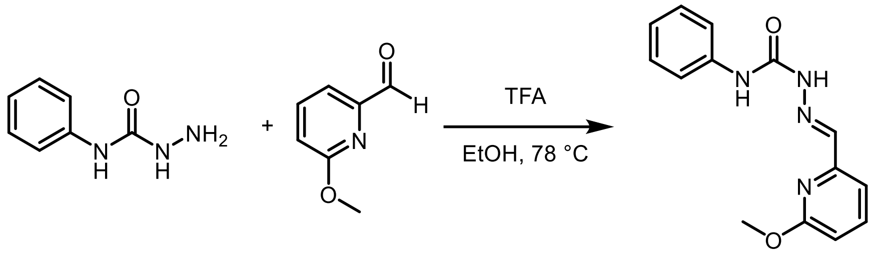

Compound was prepared according to our previous report.[3] A suspension of 6-methoxypicolinaldehyde (0.20 mL, 1.66 mmol, 1.1 equiv) and compound 4-phenylcarbazide (219.6 mg, 1.45 mmol, 1.0 equiv) in ethanol (10 mL) was treated with two drops of trifluoroacetic acid (TFA). The mixture was heated to reflux (heat-gun assisted) until complete dissolution of the starting materials, then stirred at room temperature for 2 h and stored at 4 °C overnight. The resulting precipitate was collected by filtration, washed with cold ethanol, and dried under reduced pressure at 50 °C to afford **O-SC2** as white crystals (338.0 mg, 1.25 mmol, 75%). ^{1}H NMR (400 MHz, $CDCl_3$, 25 °C) δ = 8.99 (s, 1H), 8.16 (s, 1H), 7.81 (s, 1H), 7.67 – 7.60 (m, 1H), 7.60 – 7.54 (m, 2H), 7.47 (d, *J* = 7.4 Hz, 1H), 7.40 – 7.29 (m, 2H), 7.15 – 6.98 (m, 1H), 6.76 (d, *J* = 8.2 Hz, 1H), 3.98 (s, 3H) ppm. ^{13}C NMR (100 MHz, $CDCl_3$, 25 °C) δ = 162.91, 152.73, 149.55, 139.63, 138.24, 132.77, 128.98, 123.15, 119.68, 118.49, 112.24, 54.31 ppm. IR: υ_{max} (cm^{-1}) = 3193, 3076 (N–H), 1677 (C=O), 1532 (C=N). MALDI-TOF MS: m/z calculated: 271.12 ([M+H]$^+$); found: 271.12.

(*Z*)-2-((6-methoxypyridin-2-yl)methylene)-N-phenylhydrazine-1-carbothioamide (S-SC2)

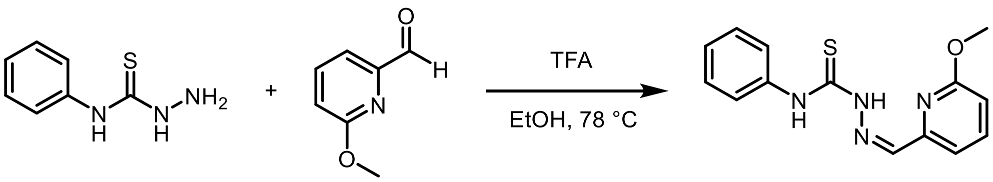

Compound was prepared according to our previous report.[3] 6-Methoxypicolinaldehyde (0.17 mL, 1.41 mmol, 1.2 equiv) and 4-phenylthiosemicarbazide (200.2 mg, 1.20 mmol, 1.0 equiv) were suspended in ethanol (10 mL) and treated with two drops of TFA. The suspension was heated to reflux until complete dissolution, then stirred at room temperature for 2 h. The resulting solid was collected by filtration, washed with ethanol, and dried under reduced pressure at 50 °C to afford **S-SC2** as a yellow powder (292.0 mg, 1.02 mmol, 85%). ^{1}H NMR (400 MHz, $CDCl_3$, 25 °C) δ = 14.33 (s, 1H), 9.45 (s, 1H), 7.78 – 7.69 (m, 3H), 7.44 – 7.35 (m, 2H), 7.25 – 7.18 (m, 1H), 7.11 (s, 1H), 7.06 (d, J = 7.3 Hz, 1H), 6.86 (d, J = 7.8 Hz, 1H), 4.16 (s, 3H) ppm. ^{13}C NMR (100 MHz, $CDCl_3$, 25 °C) δ = 176.08, 163.05, 148.97, 139.61, 138.11, 133.57, 128.75, 125.75, 123.71, 119.03, 113.04, 55.33 ppm. IR: υ_{max} (cm^{-1}) = 3289, 3134 (N–H), 1526 (C=N), 1152 (C=S). MALDI-TOF MS: m/z calculated: 287.12 ($[M+H]^+$); found: 287.12

(Z)-2-((6-methoxypyridin-2-yl)methylene)-N-phenylhydrazine-1-carboselenoamide (Se-SC2)

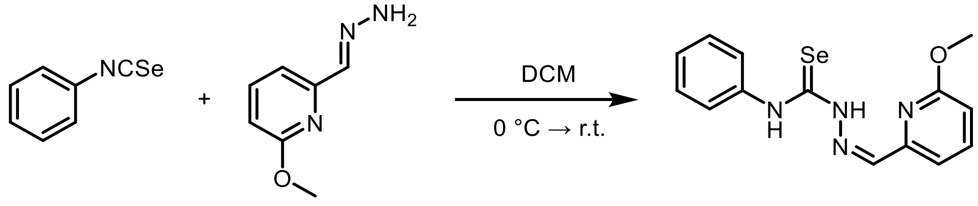

A solution of 6-methoxy-2-pyridinecarboxaldehyde hydrazone (415 mg, 2.75 mmol, 1.0 equiv) in anhydrous DCM (20 mL) was cooled to 0 °C in an ice bath. Compound **1.Se** (500 mg, 2.75 mmol, 1.0 equiv) was added dropwise. After the addition was complete, the mixture was stirred at room temperature for 1.5 h. The resulting solid was collected by vacuum filtration, washed with cold DCM, and dried under reduced pressure at 50 °C to afford **Se-SC2** as orange crystals (673 mg, 2.02 mmol, 74%). ^{1}H NMR (400 MHz, $CDCl_3$, 25 °C) δ = 14.61 (s, 1H), 9.70 (s, 1H), 7.77 – 7.70 (m, 3H), 7.45 – 7.36 (m, 2H), 7.31 – 7.27 (m, 1H), 7.18 (s, 1H), 7.08 (d, J = 7.3 Hz, 1H), 6.94 – 6.87 (m, 1H), 4.21 (s, 3H) ppm. ^{13}C NMR (100 MHz, $CDCl_3$, 25 °C) δ = 175.35, 163.19, 148.81, 139.66, 138.53, 134.69, 128.78, 126.51, 124.68, 119.33, 113.50, 54.04 ppm. IR: υ_{max} (cm^{-1}) = 3286, 3140 (N–H), 1477 (C=N), 795 (C=Se). MALDI-TOF MS: m/z calculated: 335.04 ($[M+H]^+$); found: 335.04.

2.1 ^{1}H and ^{13}C NMR Spectra

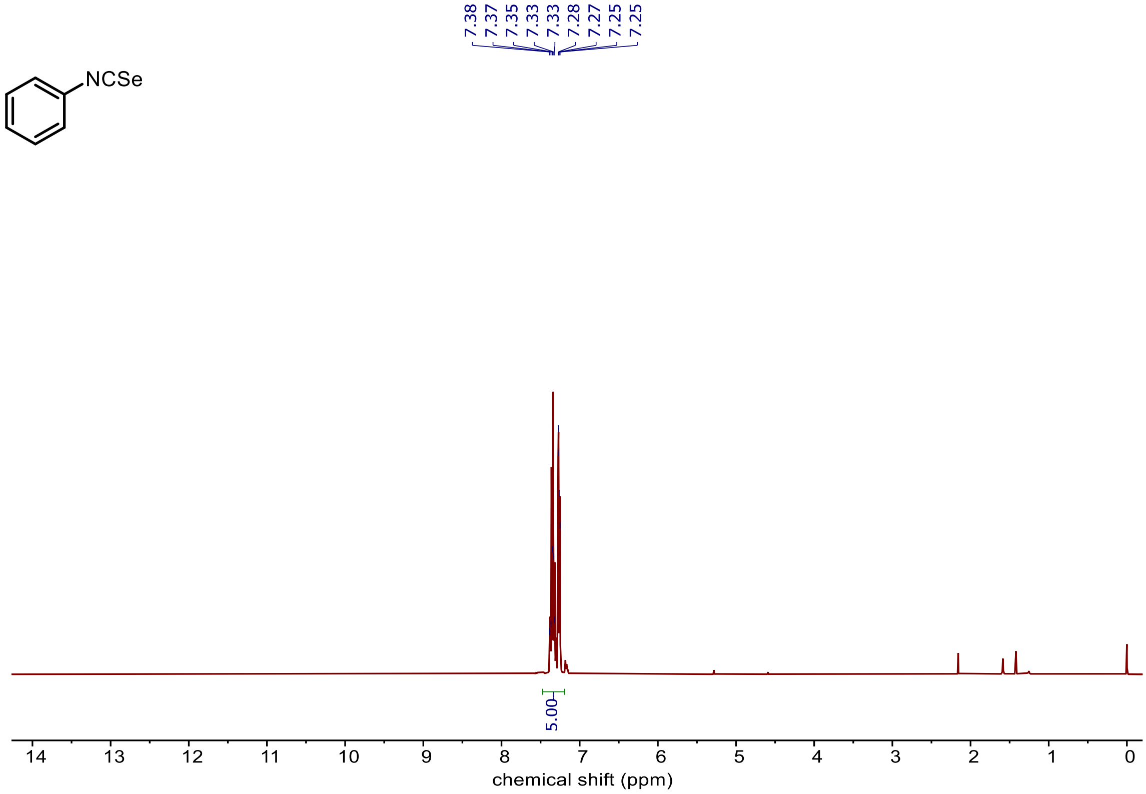

Figure S1. ^{1}H NMR (400 MHz, $CDCl_3$, 25 °C) spectrum of **Se-1**.

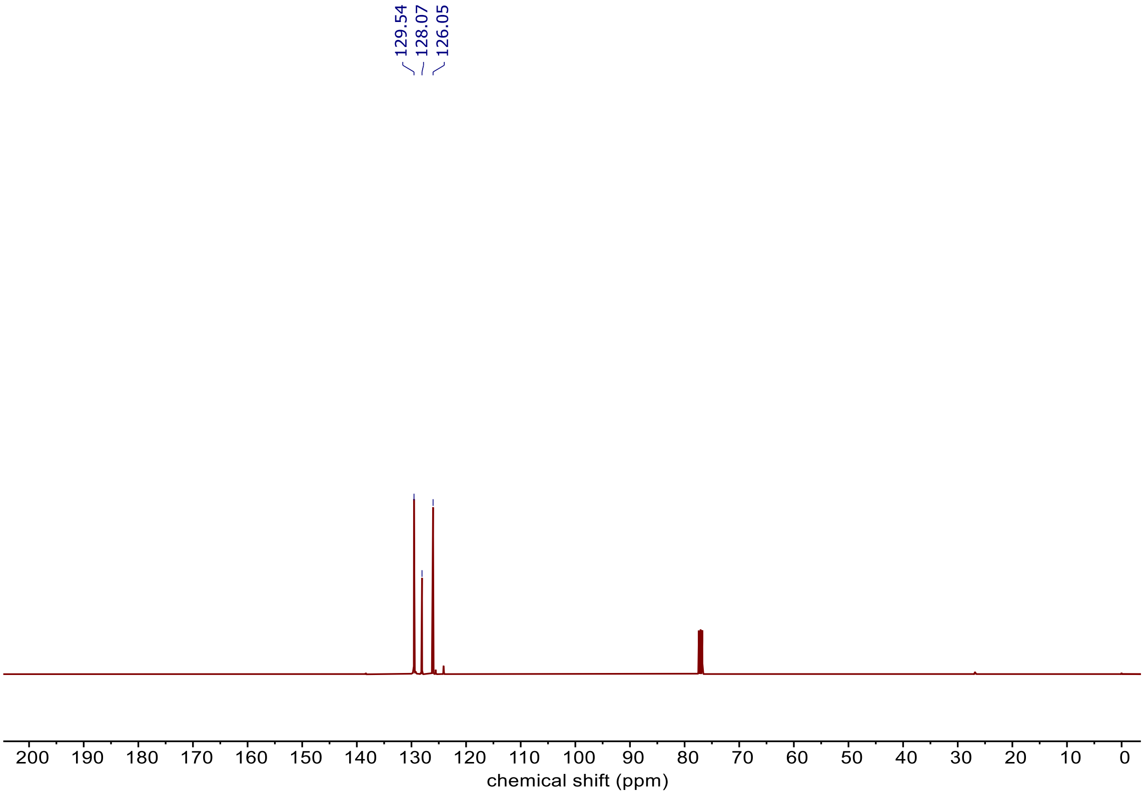

Figure S2. ^{13}C NMR (100 MHz, $CDCl_3$, 25 °C) spectrum of **Se-1**.

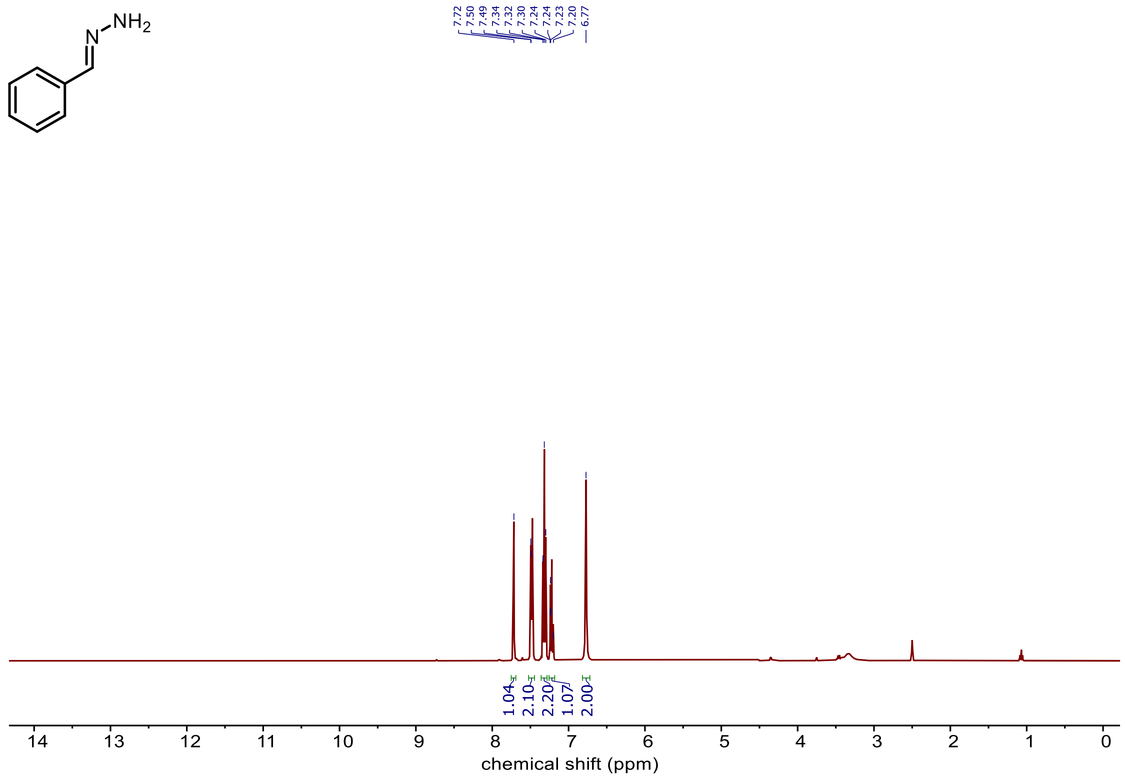

Figure S3. ^{1}H NMR (400 MHz, DMSO-d_6, 25 °C) spectrum of **H-1**.

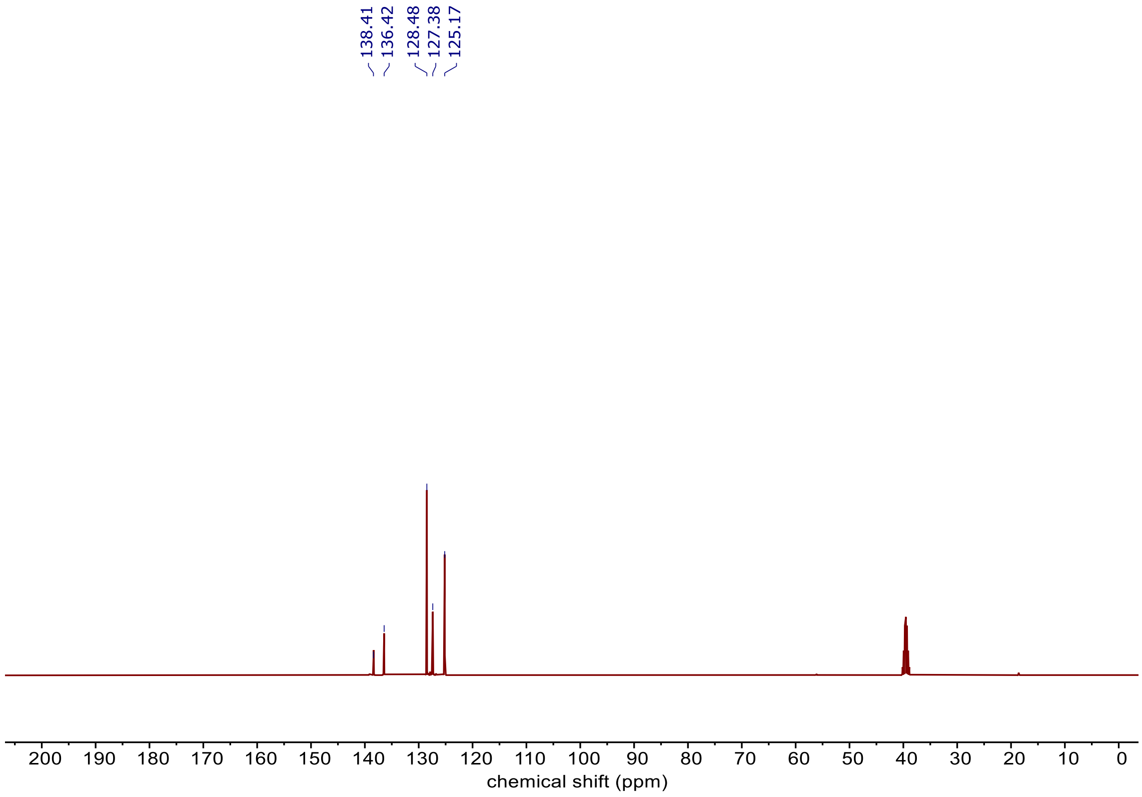

Figure S4. ^{13}C NMR (100 MHz, DMSO-d_6, 25 °C) spectrum of **H-1**.

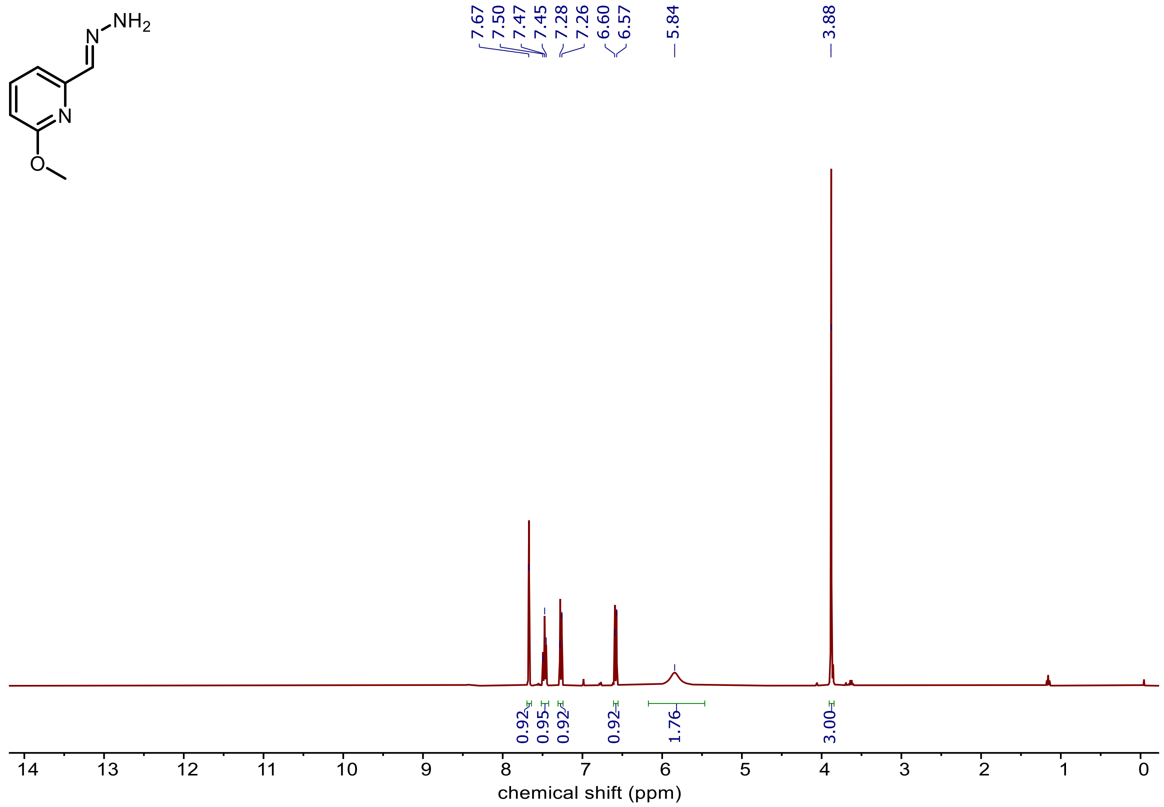

Figure S5. ^{1}H NMR (400 MHz, $CDCl_3$, 25 °C) spectrum of **H-2**.

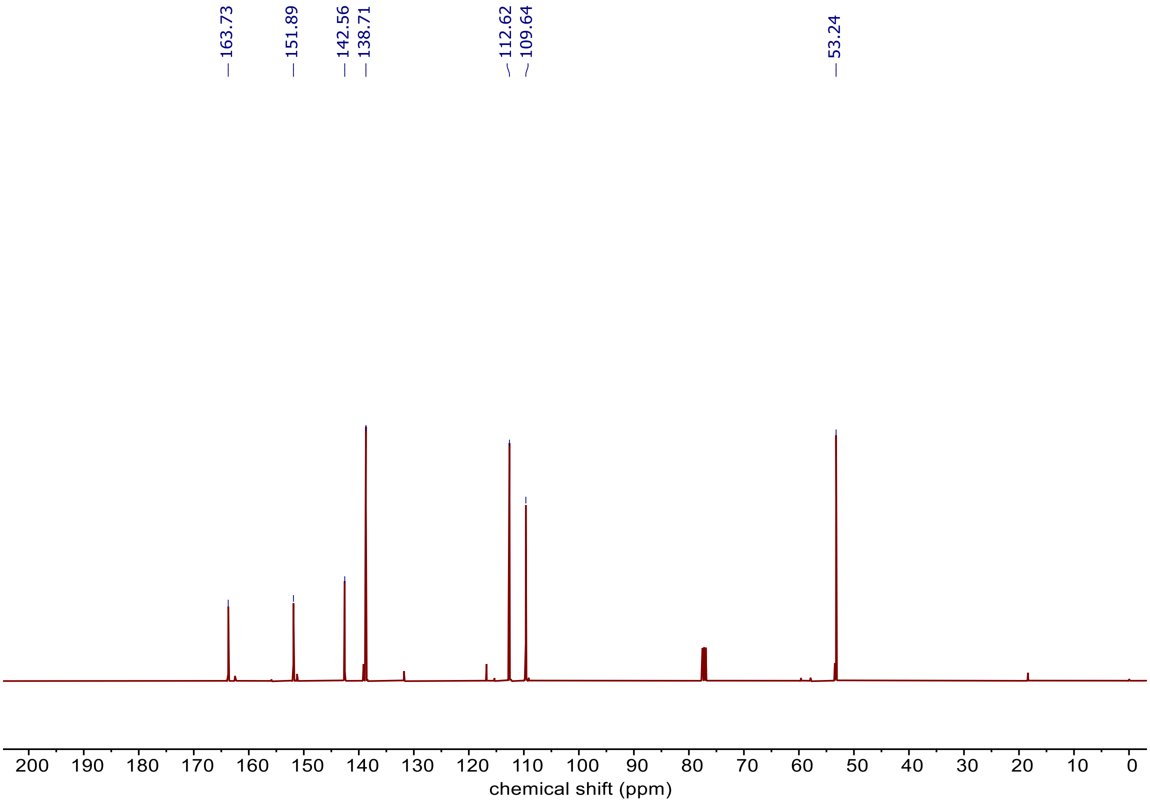

Figure S6. ^{13}C NMR (100 MHz, $CDCl_3$, 25 °C) spectrum of **H-2**.

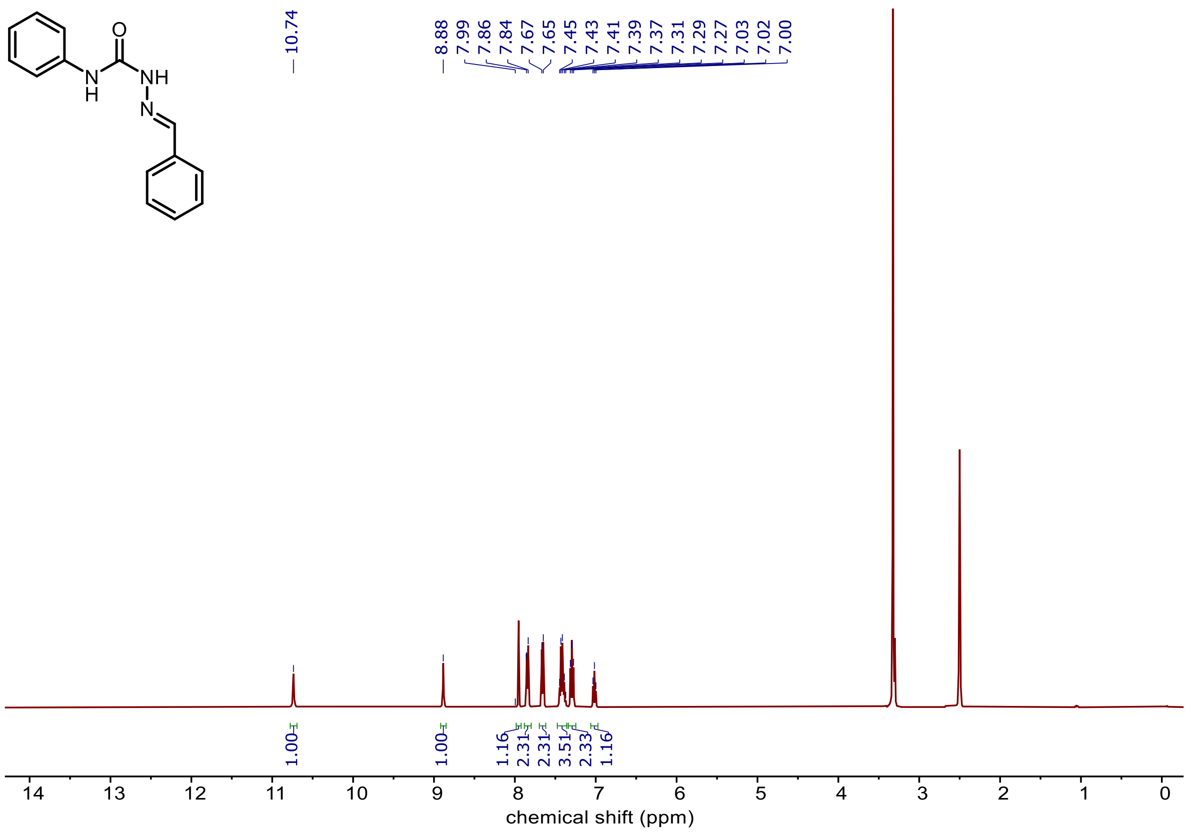

Figure S7. ^{1}H NMR (400 MHz, DMSO-d_6, 25 °C) spectrum of **O-SC1**.

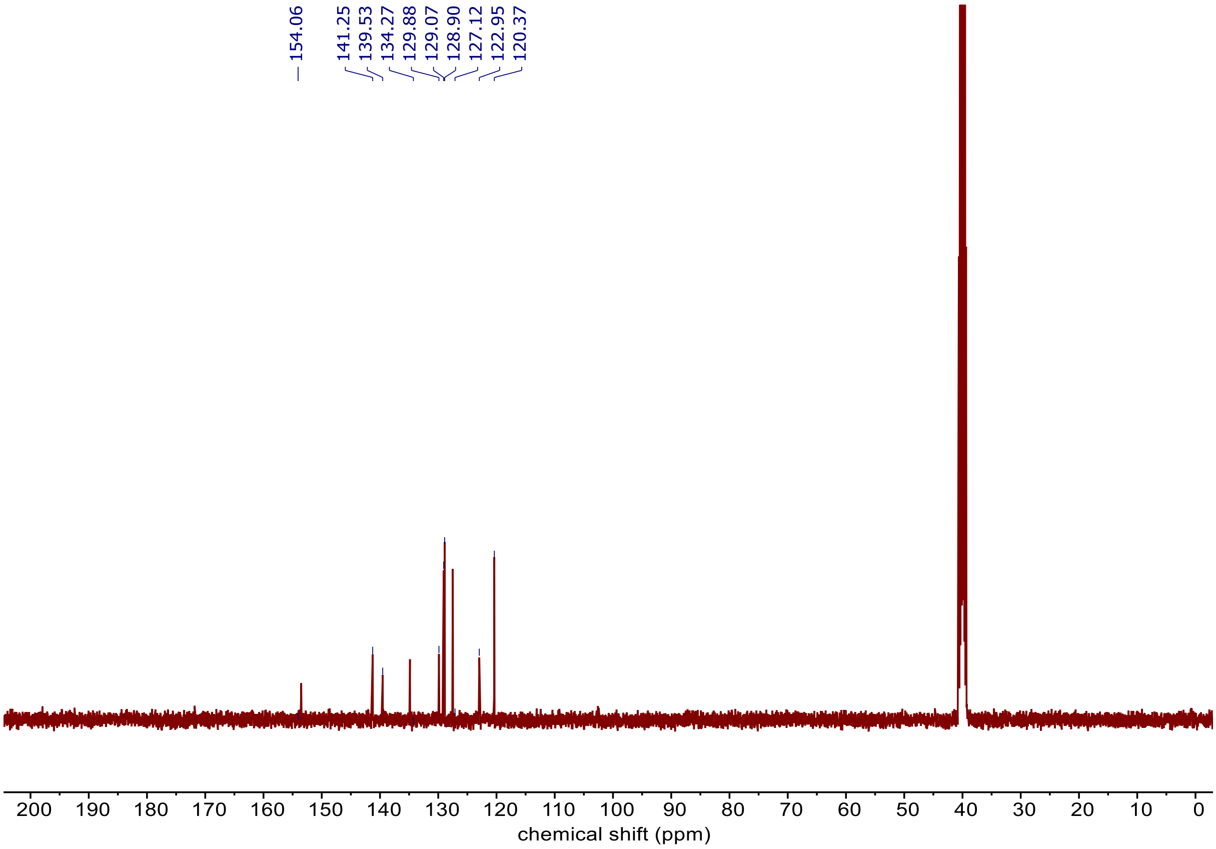

Figure S8. ^{13}C NMR (100 MHz, DMSO-d_6, 25 °C) spectrum of **O-SC1**.

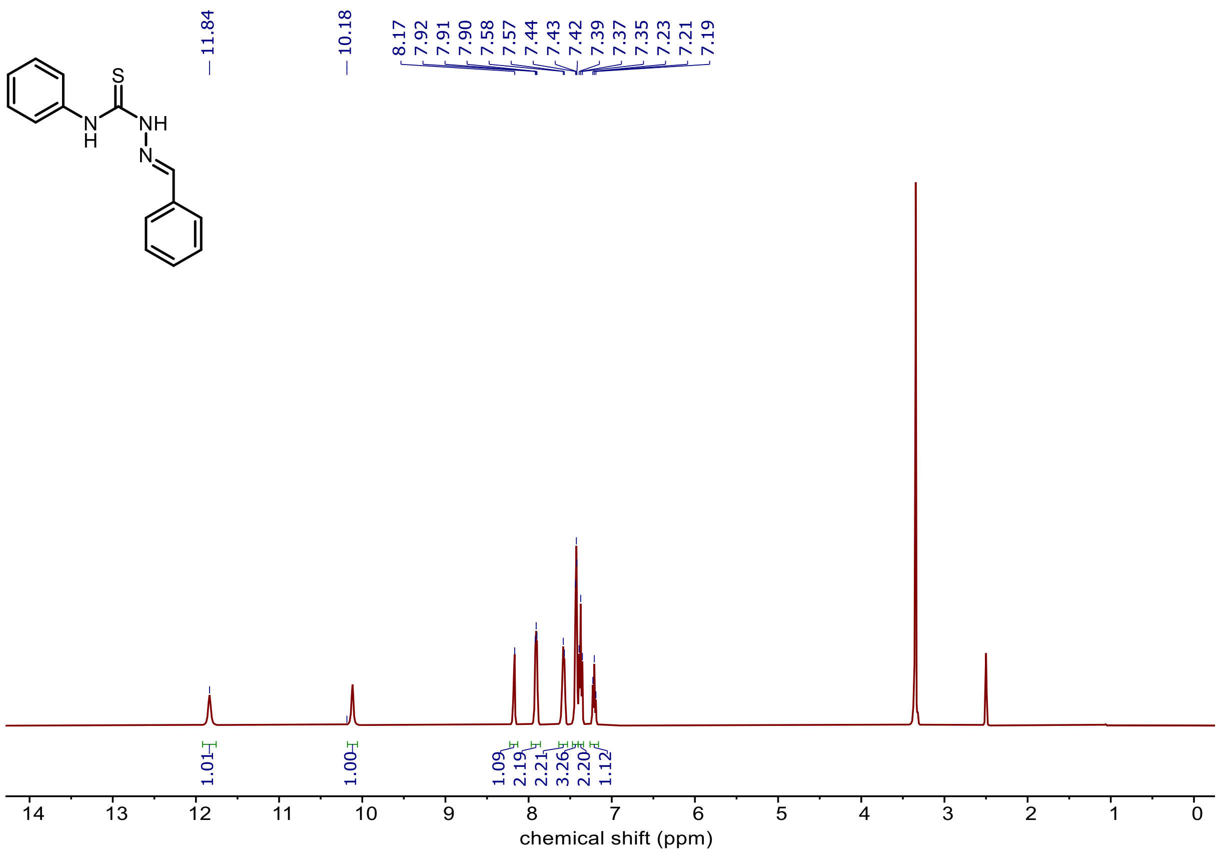

Figure S9. ^{1}H NMR (400 MHz, DMSO-d_6, 25 °C) spectrum of **S-SC1**.

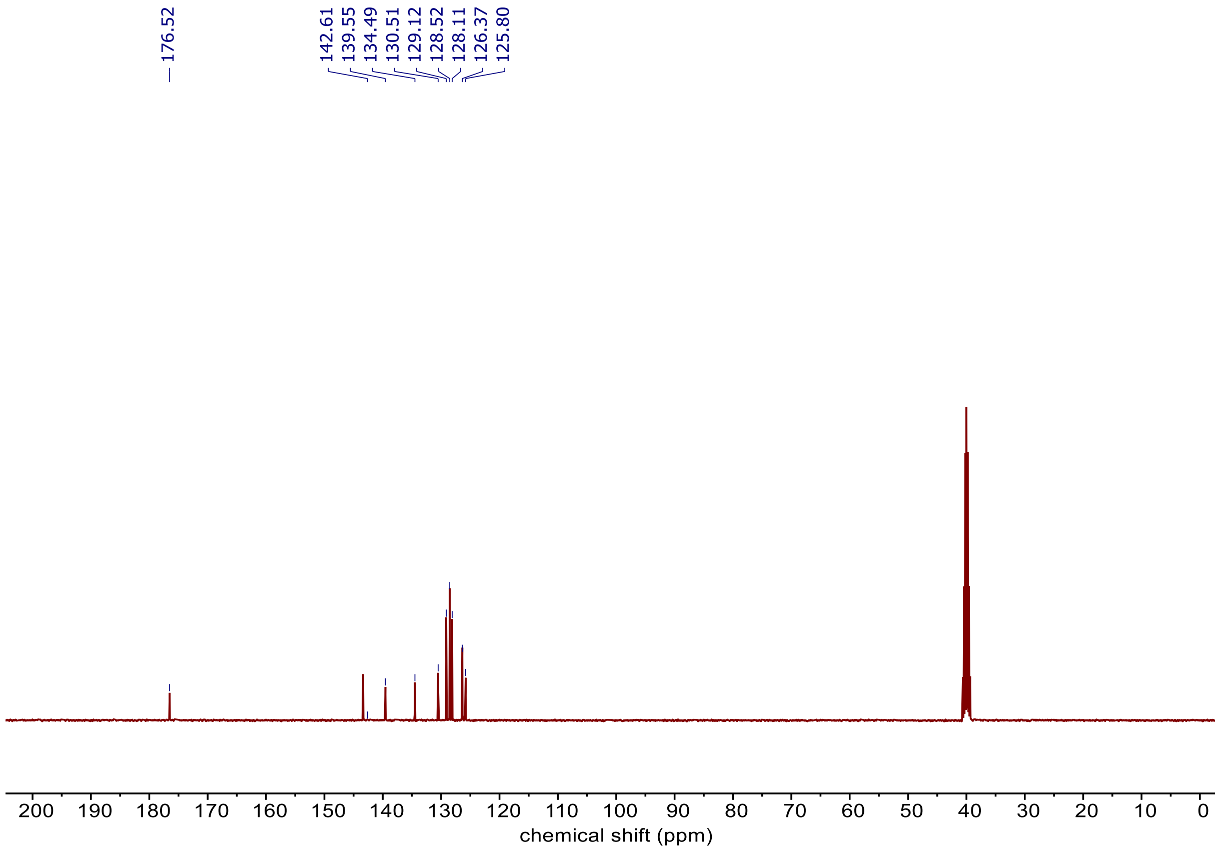

Figure S10. ^{13}C NMR (100 MHz, DMSO-d_6, 25 °C) spectrum of **S-SC1**.

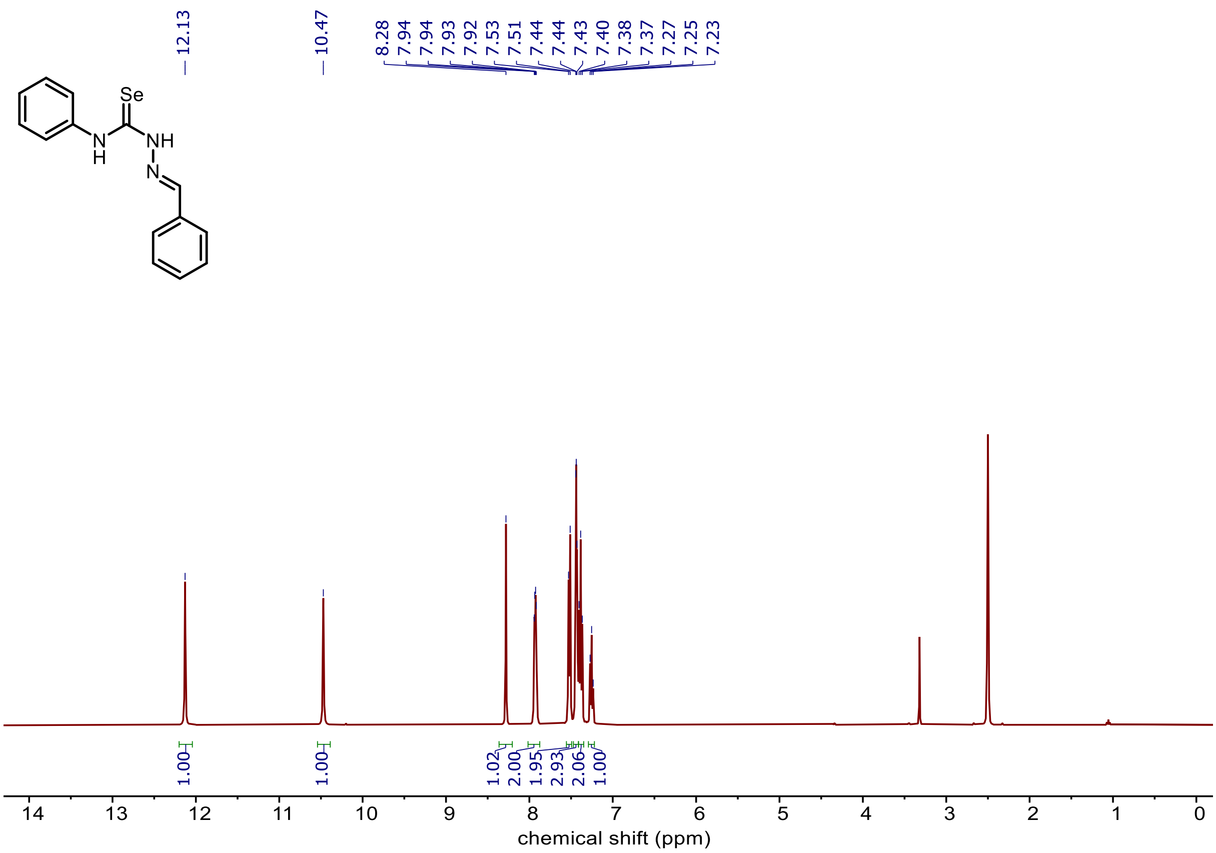

Figure S11. ^{1}H NMR (400 MHz, DMSO-d_6, 25 °C) spectrum of **Se-SC1**.

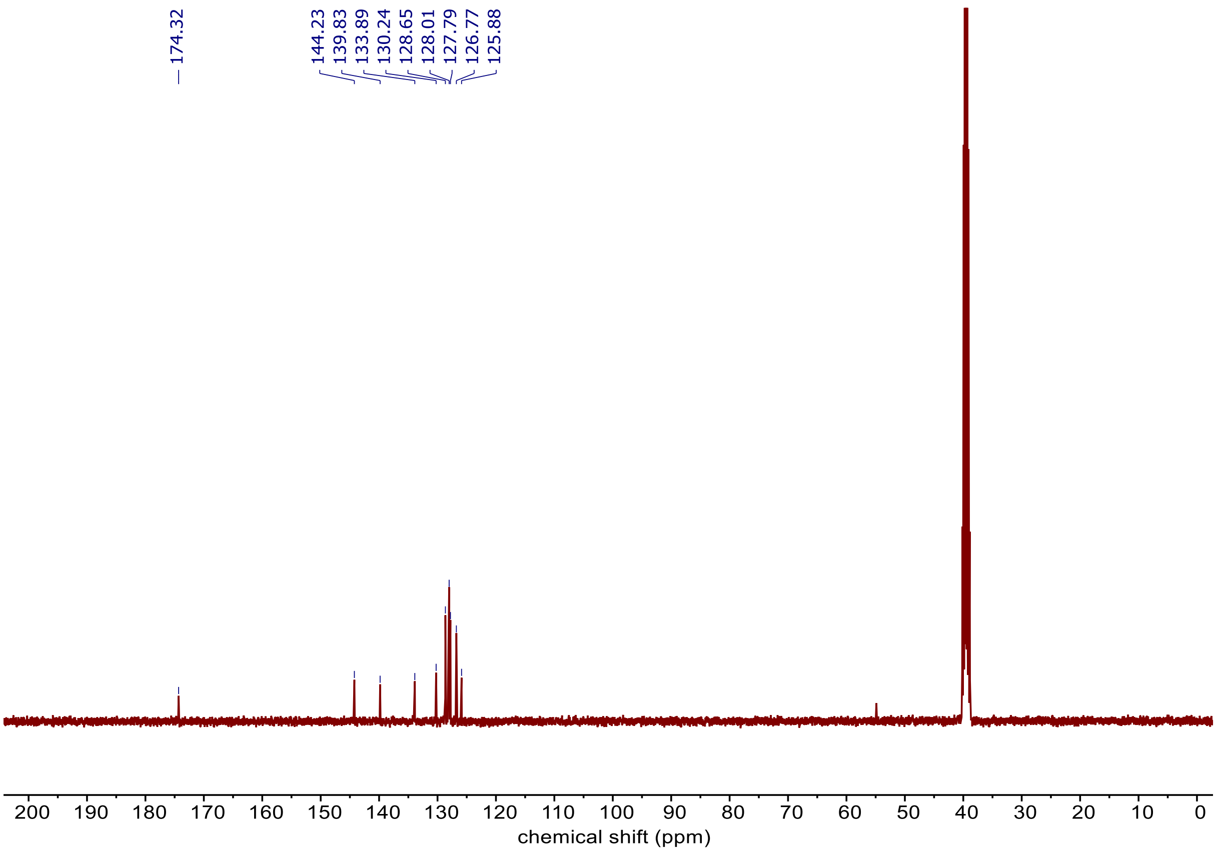

Figure S12. ^{13}C NMR (100 MHz, DMSO-d_6, 25 °C) spectrum of **Se-SC1**.

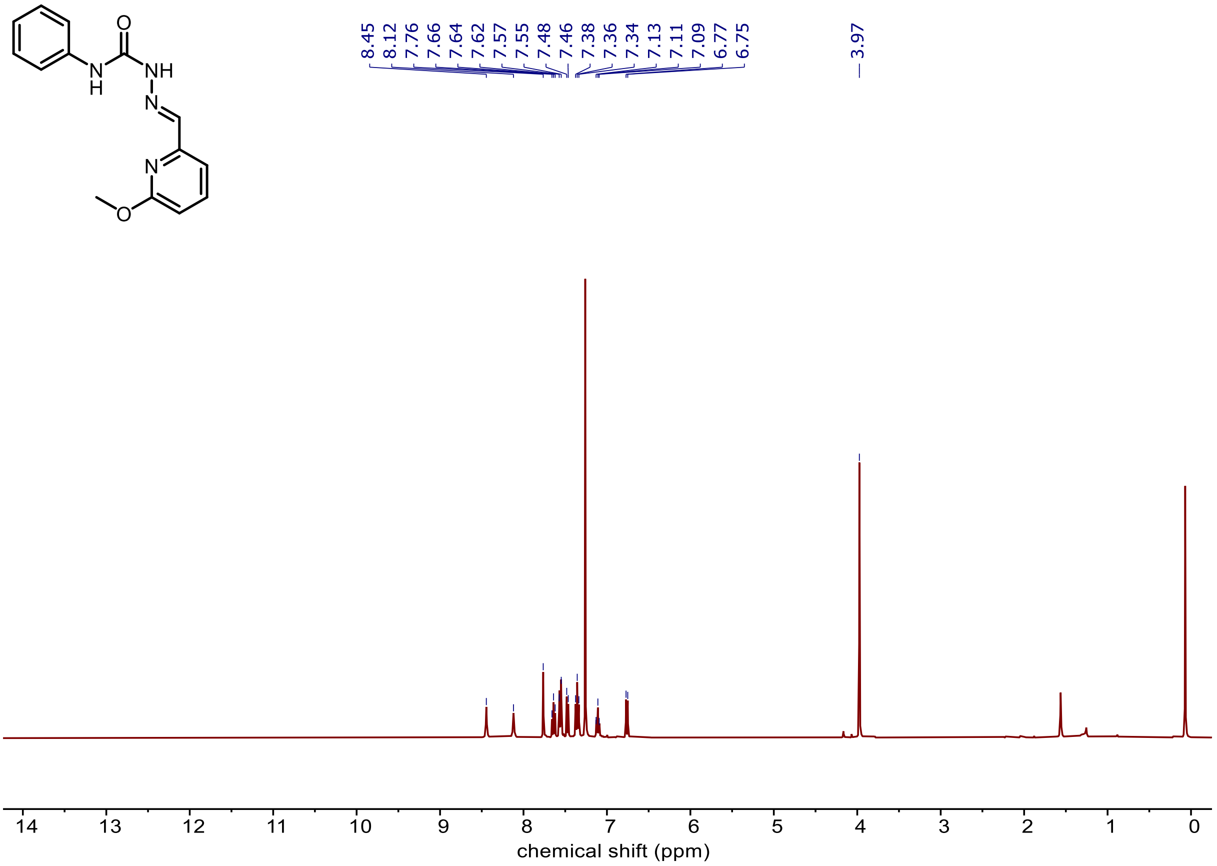

Figure S13. ^{1}H NMR (400 MHz, $CDCl_3$, 25 °C) spectrum of **O-SC2**.

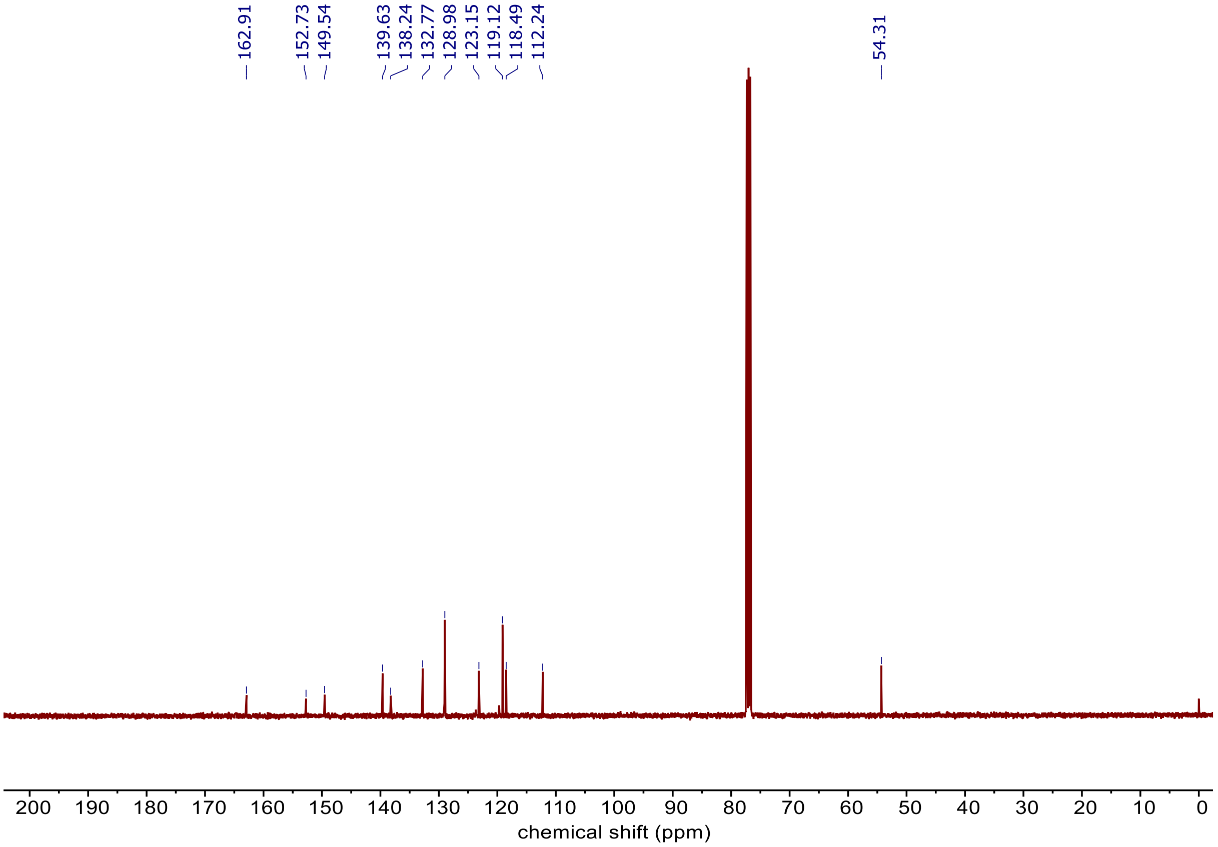

Figure S14. ^{13}C NMR (100 MHz, $CDCl_3$, 25 °C) spectrum of **O-SC2**

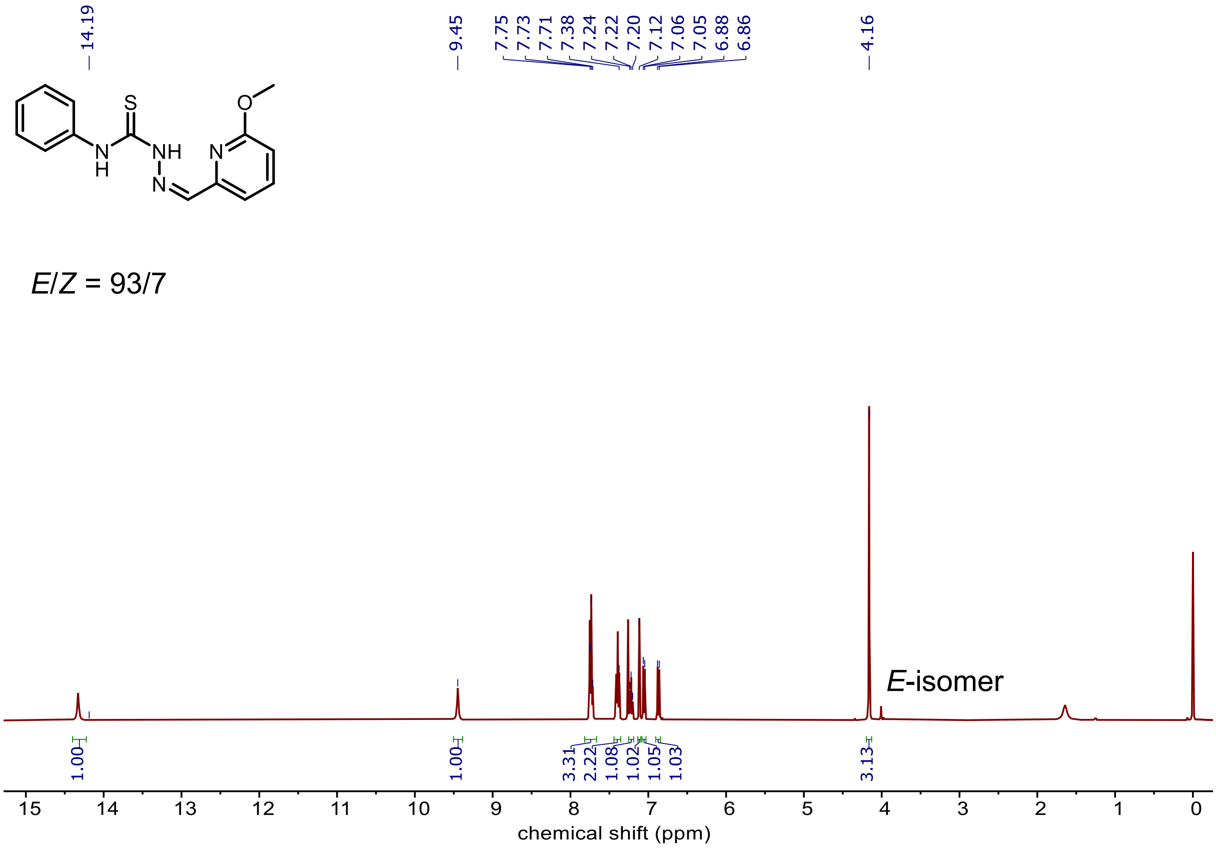

Figure S15. ^{1}H NMR (400 MHz, $CDCl_3$, 25 °C) spectrum of **S-SC2**.

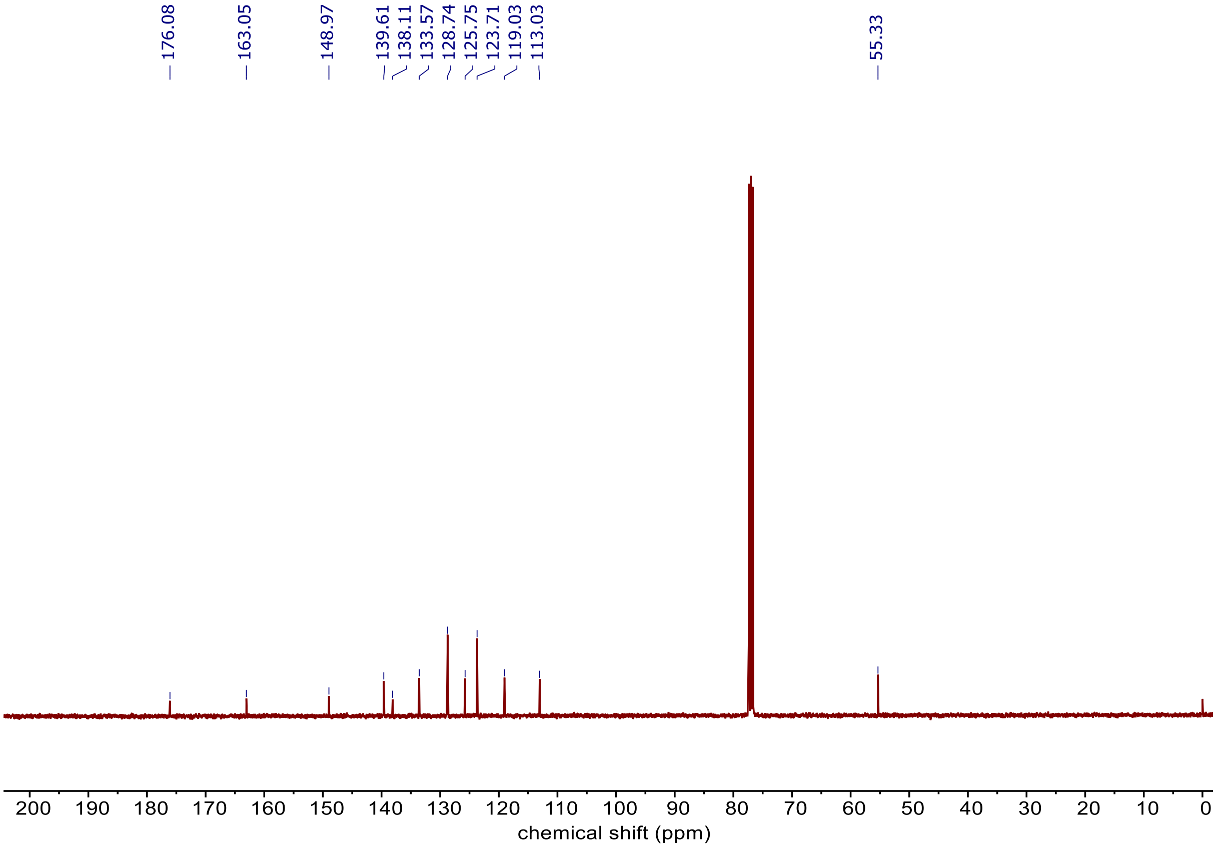

Figure S16. ^{13}C NMR (100 MHz, $CDCl_3$, 25 °C) spectrum of **S-SC2**.

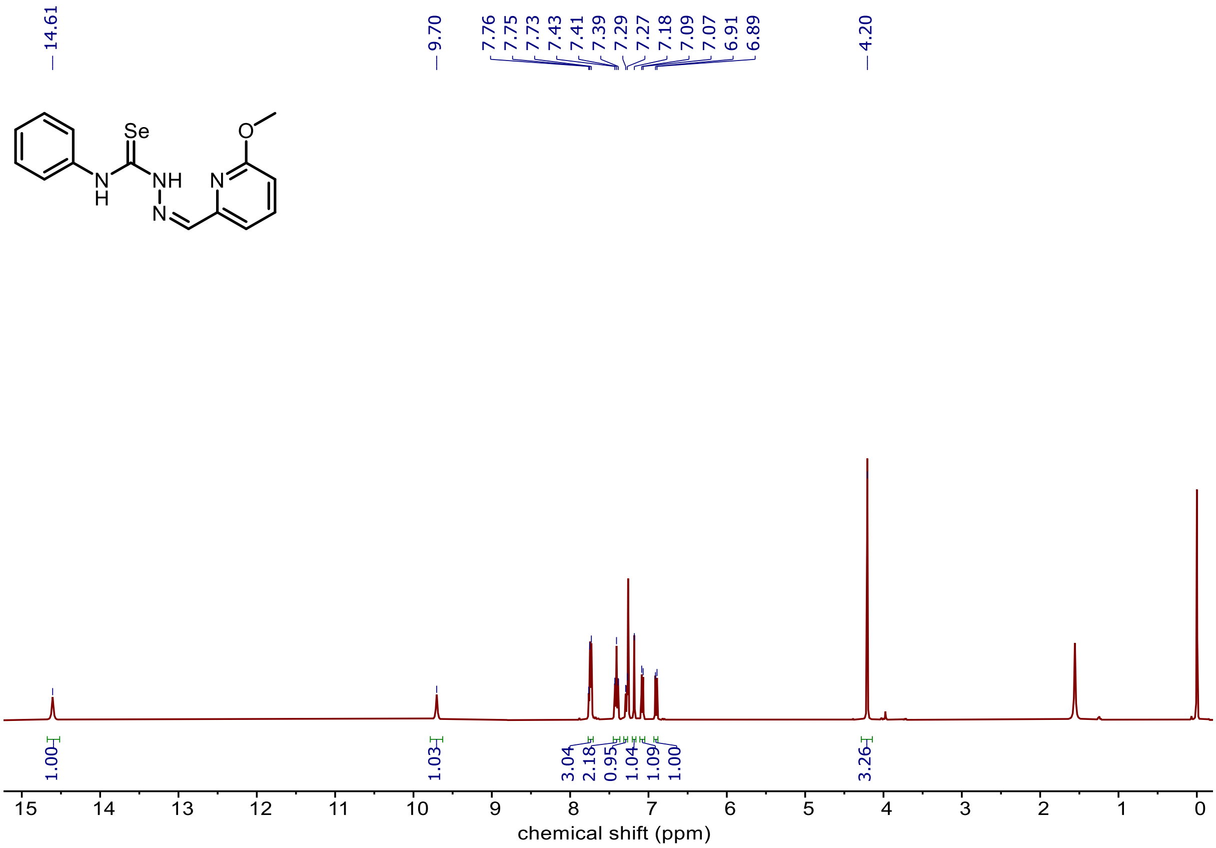

Figure S17. ^{1}H NMR (400 MHz, $CDCl_3$, 25 °C) spectrum of **Se-SC2**.

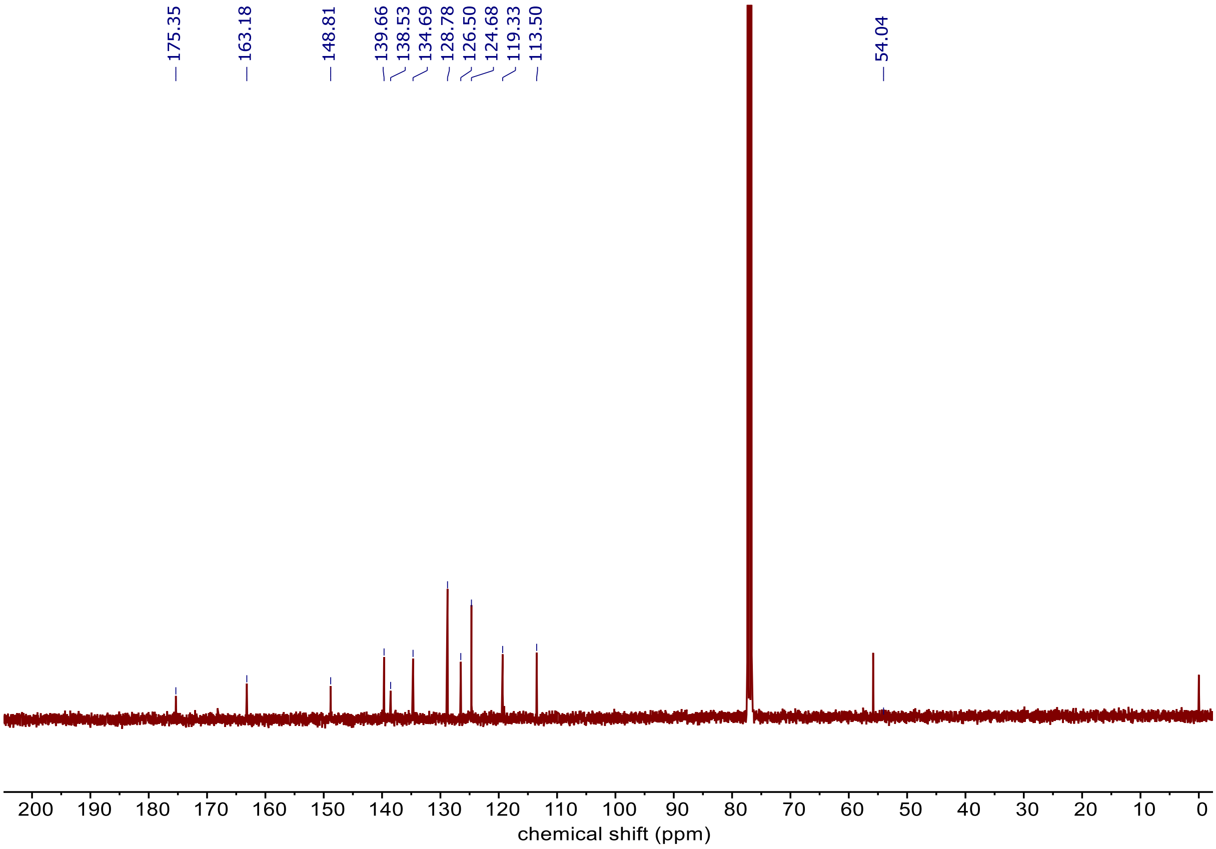

Figure S18. ^{13}C NMR (100 MHz, $CDCl_3$, 25 °C) spectrum of **Se-SC2**.

3. Photophysical and Photochemical Properties

After synthesis, the compounds were isolated from ethanol, with the assumption that they primarily adopt the *E*-isomer configuration. To allow equilibration, each compound was dissolved in 2 mL chloroform and heated to 50 °C for 2 h. After cooling to room temperature, the solution was transferred into a 100 mL volumetric flask and diluted to the mark with chloroform. Subsequently, 0.2 mL deionized water was added, and the mixture was sonicated for several minutes before UV–vis measurements. Unless stated otherwise, all UV–vis and photochemical experiments were carried out with solutions prepared in this way.

For each semicarbazone, three independent solutions were prepared. Thermal back-relaxation was monitored in the dark by UV–vis spectroscopy, tracking the absorbance at the compound's λ_{max} until it returned to the initial pre-irradiation state. Kinetic traces were fitted to a mono-exponential function. Lifetimes of the metastable state were obtained by non-linear regression using the Levenberg–Marquardt algorithm and converted to half-lives by multiplying with ln2. Reported values represent the mean from three independent experiments.

3.1. Influence of residual acidity on photoisomerization behaviour

Photoisomerization experiments in chloroform were not always reproducible. For example, duplicate solutions of **Se-SC1** prepared by identical procedures gave markedly different photostationary state (PSS) values. In one case, irradiation produced almost no spectral change. To test whether variable water content in the solvent was responsible, deionized water was deliberately added to the solutions to saturate the system, leading to visible phase separation. Absorption spectra were collected before and after water addition (**Figure S19a and b**). In the solution that was initially photoswitchable, no substantial changes were observed after water addition. In contrast, solutions that initially showed poor switching behaviour displayed significantly improved photoisomerization after water saturation. Additional experiments using chloroform neutralized with basic aluminum oxide followed by storage over 3 Å molecular sieves likewise resulted in efficient and reproducible photoisomerization behaviour. Comparison of the thermal back-relaxation kinetics revealed that alumina-treated chloroform produced substantially longer and more reproducible thermal half-lives than untreated chloroform, comparable to those observed in water-saturated chloroform (**Figure S19c-d**). Together, these observations indicate that controlling residual acidity in chloroform is important for achieving reproducible photoisomerization behavior and consistent thermal relaxation kinetics.

a) Solution-1

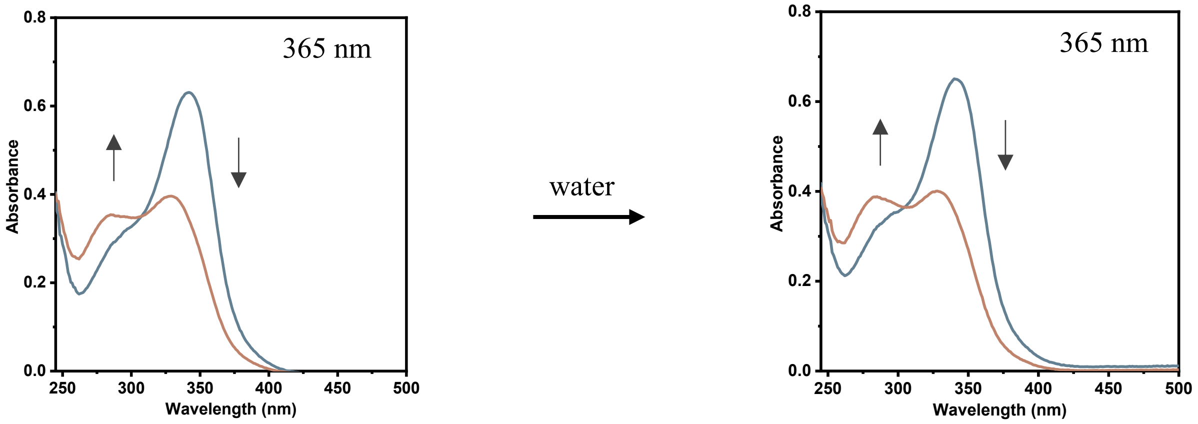

b) Solution-2

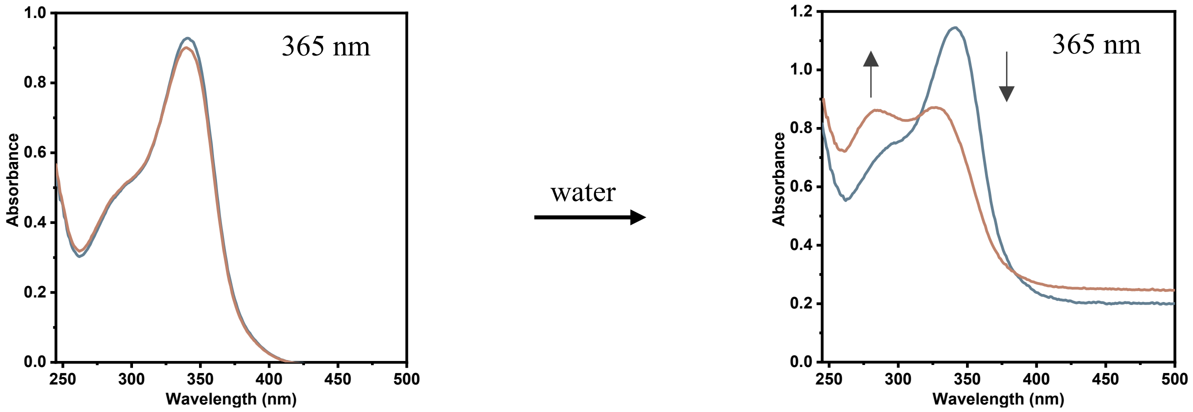

c) Solution-3

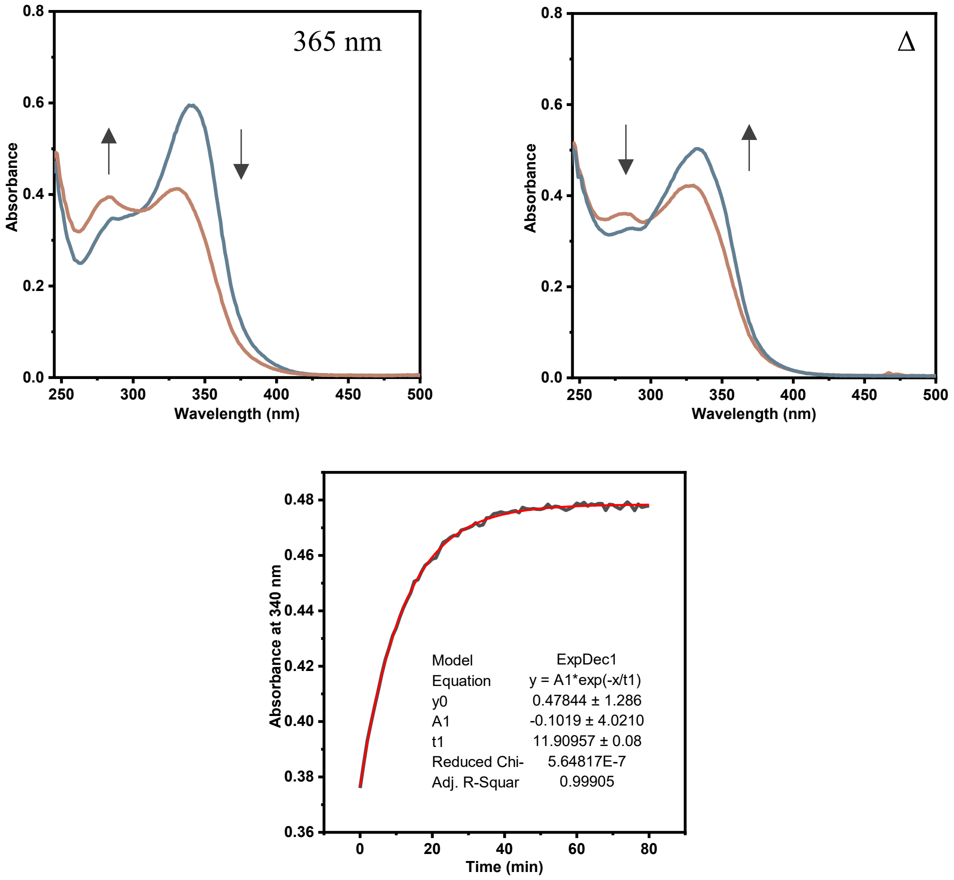

d) Solution-4

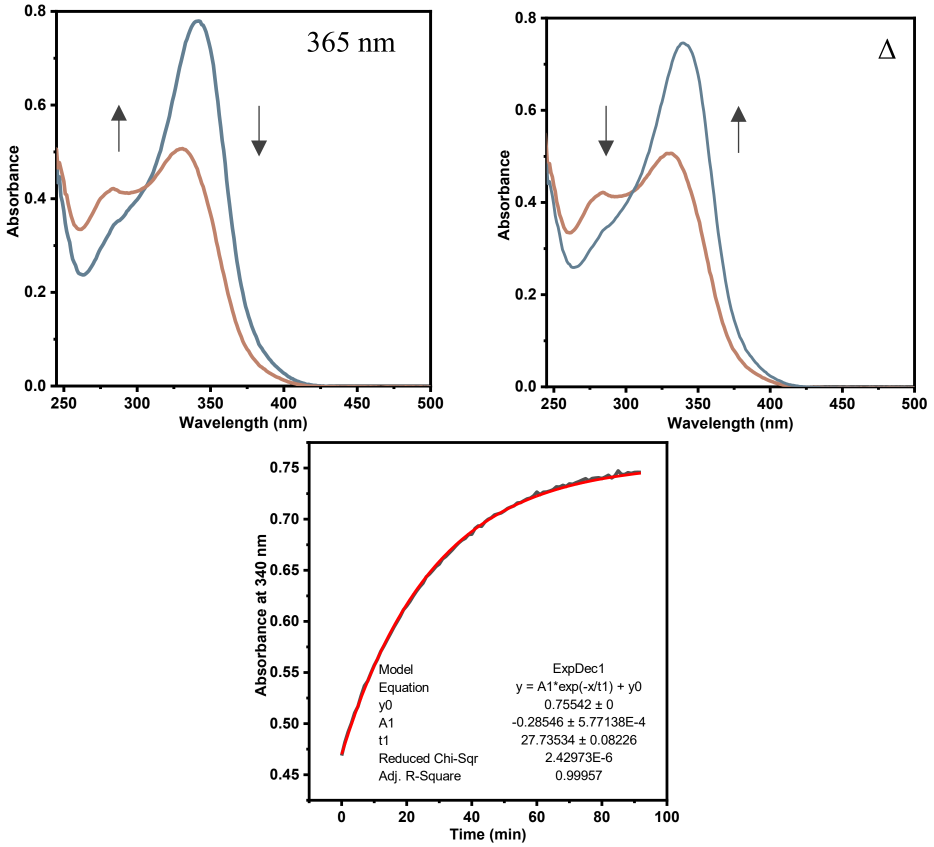

Figure S19. a) Conversion of **Se-SC1*E*** (blue line) to **Se-SC1*Z,PSS*** (orange line) in solution-1 (c = $4.3\cdot10^{-5}$ M, λ_{irr} = 365 nm); chloroform without added water (left) and in water-saturated chloroform (right). **b)** Conversion of **Se-SC1*E*** (blue line) to **Se-SC1*Z,PSS*** (orange line) in solution-2 (c = $3.8\cdot10^{-5}$ M, λ_{irr} = 365 nm); chloroform without added water (left) and in water-saturated chloroform (right). The baseline shifted due to addition of water to the existing solution. **c)** Conversion of **Se-SC1*E*** (blue line) to **Se-SC1*Z,PSS*** (orange line) in solution-3 (c = $2.4\cdot10^{-5}$ M, λ_{irr} = 365 nm); chloroform without added water (upper left), thermal back-relaxation of that solution at 20 °C (upper right), and regression of the thermal-back relaxation data (bottom). **d)** Conversion of **Se-SC1*E*** (blue line) to **Se-SC1*Z,PSS*** (orange line) in solution-4 (c = $3.1\cdot10^{-5}$ M, λ_{irr} = 365 nm); chloroform neutralized with basic aluminum oxide (upper left) followed by storage over 3 Å molecular sieves, thermal back-relaxation of that solution at 20 °C (upper right), and regression of the thermal-back relaxation data (bottom).

While the precise mechanism remains uncertain, several factors may contribute. First, water may disrupt intermolecular interactions between *E*-isomers, thereby lowering the barrier for isomerization. Alternatively, water could alter the local electronic environment and excited-state dynamics, shifting the PSS toward higher *Z*-isomer content. This interpretation is consistent with earlier studies showing that trace amounts of water in alkane-based solvents disrupt supramolecular interactions and strongly influence the stability of hydrogen-bonded structures.[4,5] Moreover, semicarbazones are known to undergo acid-catalyzed *Z*→*E* back-isomerization.[6] Protonation of the imine nitrogen by trace acidic sites (e.g., HCl in chloroform)

would lower the C=N rotational barrier, allowing rapid thermal reversion to the *E*-isomer. Addition of water can suppress this pathway by hydrogen bonding to acidic sites, thereby reducing their effective acidity and stabilizing the photogenerated *Z*-isomer (**Scheme S1**).

Based on these findings, all subsequent photoisomerization experiments in chloroform were performed using water-saturated solvent to ensure reproducibility.

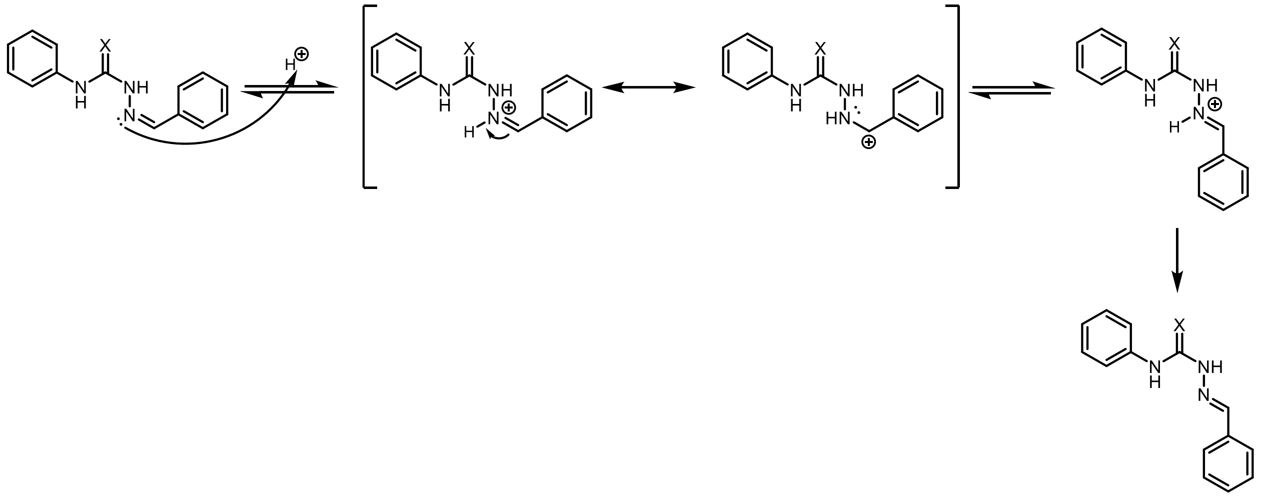

Scheme S1. Proposed mechanism for acid-catalyzed *Z*→*E* back-isomerization of semicarbazones.

3.2. Photoswitching Studies

3.2.1. O-SC1

a)

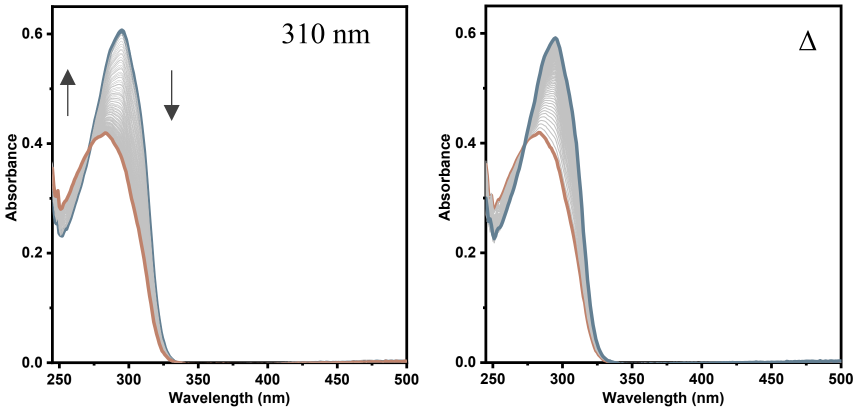

b)

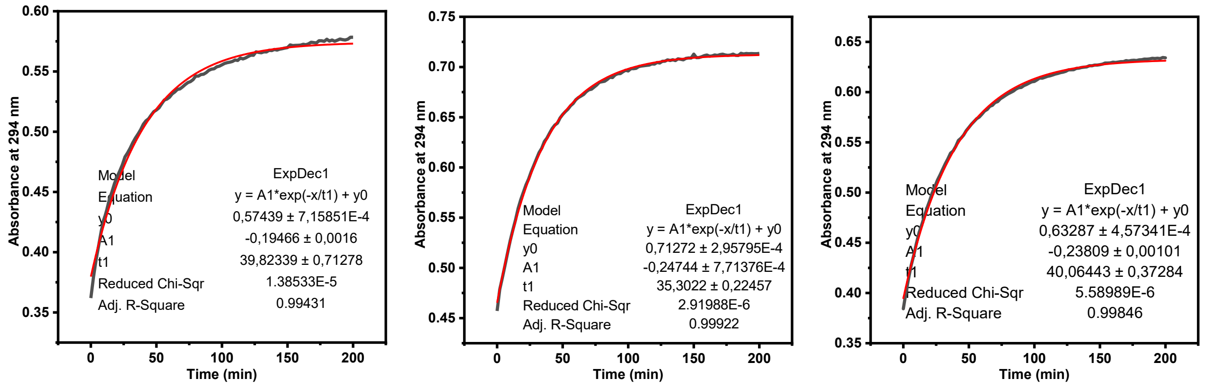

Figure S20. a) Conversion of **O-SC1*E*** (blue line) in water-saturated chloroform ($2.83 \cdot 10^{-5}$ M) to **O-SC1*Z-PSS*** (orange line) followed over time under constant light irradiation (λ_{irr} = 310 nm) (left). Thermal back-relaxation of **O-SC1*Z-PSS*** at 20 °C (right). **b)** Regression of the thermal-back relaxation data, along with data from two additional solutions undergoing thermal back-relaxation.

3.2.2. S-SC1

a)

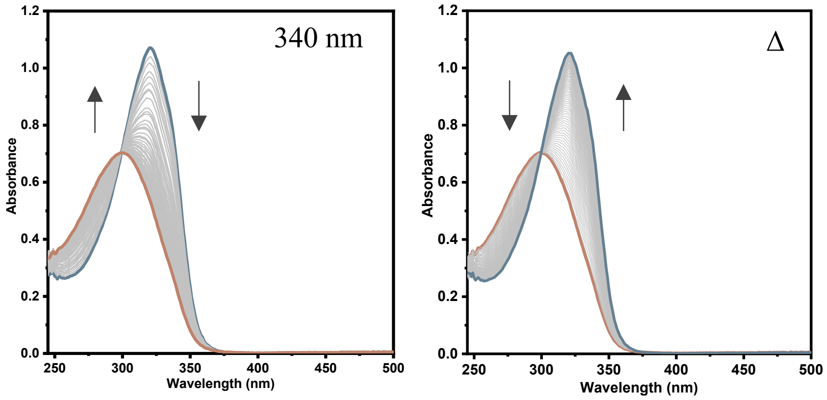

b)

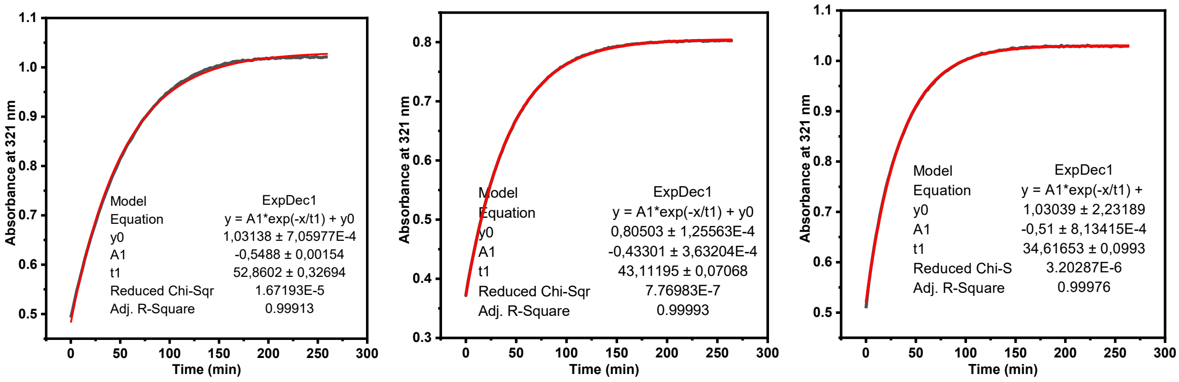

Figure S21. a) Conversion of **S-SC1*E*** (blue line) in water-saturated chloroform ($3.25 \cdot 10^{-5}$ M) to **S-SC1*Z-PSS*** (orange line) followed over time under constant light irradiation (λ_{irr} = 340 nm) (left). Thermal back-relaxation of **S-SC1*Z-PSS*** at 20 °C (right). **b)** Regression of the thermal-back relaxation data, along with data from two additional solutions undergoing thermal back-relaxation.

3.2.3. Se-SC1

a)

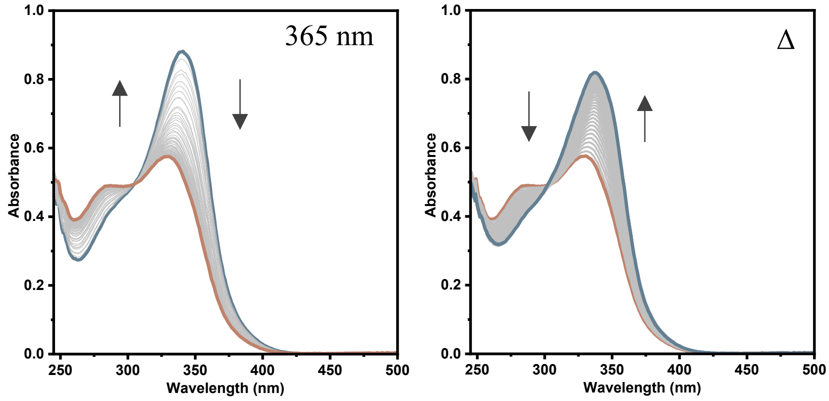

b)

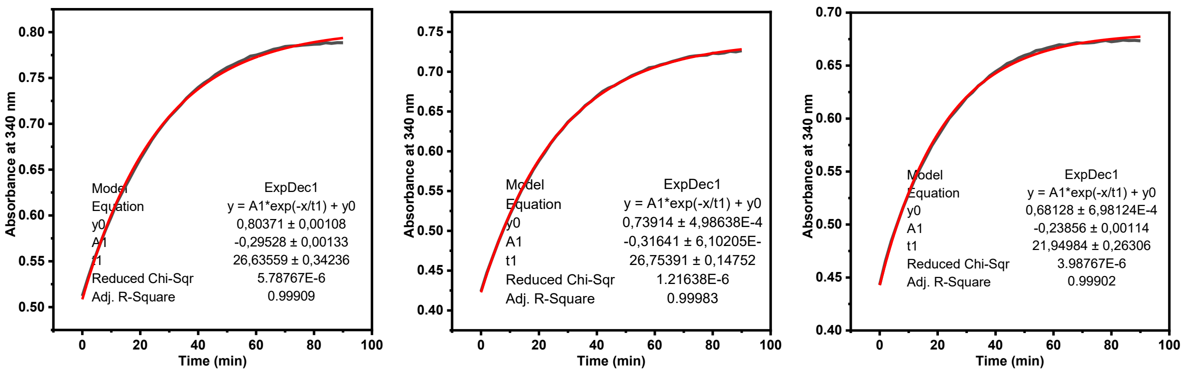

c)

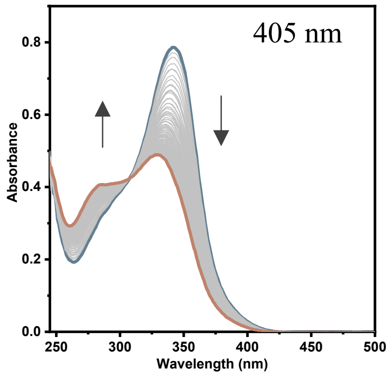

Figure S22. a) Conversion of **Se-SC1$_{\boldsymbol{E}}$** (blue line) in water-saturated chloroform ($3.41\cdot10^{-5}$ M) to **Se-SC1$_{\boldsymbol{Z\text{-}PSS}}$** (orange line) followed over time under constant light irradiation (λ_{irr} = 365 nm) (left). Thermal back-relaxation of **Se-SC1$_{\boldsymbol{Z\text{-}PSS}}$** at 20 °C (right). **b)** Regression of the thermal-back relaxation data, along with data from two additional solutions undergoing thermal back-relaxation.**c)** Conversion of **Se-SC1$_{\boldsymbol{E}}$** (blue line) in water-saturated chloroform ($2.96\cdot10^{-5}$ M) to **Se-SC1$_{\boldsymbol{Z\text{-}PSS}}$** (orange line) followed over time under constant light irradiation (λ_{irr} = 405 nm).

3.2.4. O-SC2

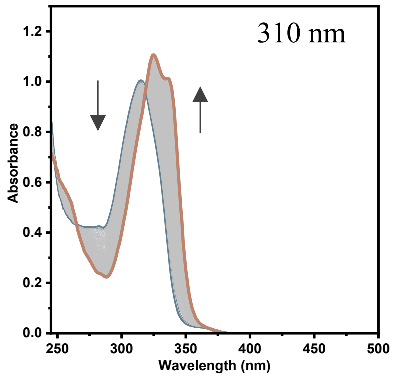

Figure S23. Conversion of **O-SC2**$_{\boldsymbol{E}}$ (blue line) in water-saturated chloroform ($4.27 \cdot 10^{-5}$ M) to **O-SC2**$_{\boldsymbol{Z\text{-}PSS}}$ (orange line) followed over time under constant light irradiation (λ_{irr} = 310 nm).

3.2.5. S-SC2

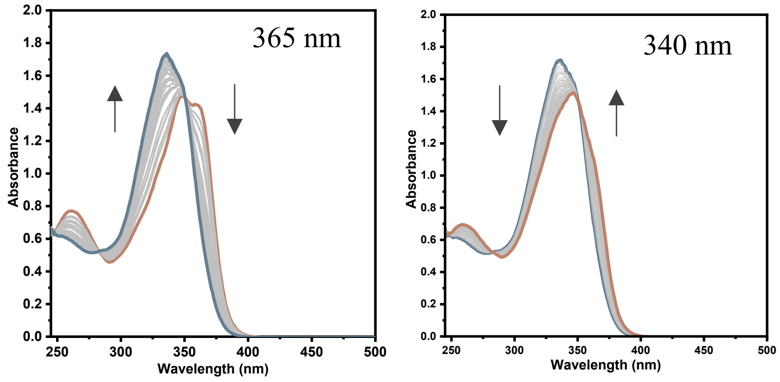

Figure S24. Conversion of **S-SC2**$_{\boldsymbol{Z}}$ (orange line) in water-saturated chloroform ($4.31 \cdot 10^{-5}$ M) to **S-SC2**$_{\boldsymbol{E\text{-}PSS}}$ (blue line) followed over time under constant light irradiation (λ_{irr} = 365 nm) (left). Conversion of **S-SC2**$_{\boldsymbol{E,PSS}}$ (blue line) in water-saturated chloroform ($4.31 \cdot 10^{-5}$ M) to **S-SC2**$_{\boldsymbol{Z\text{-}PSS}}$ (orange line) followed over time under constant light irradiation (λ_{irr} = 340 nm) (right).

3.2.6. Se-SC2

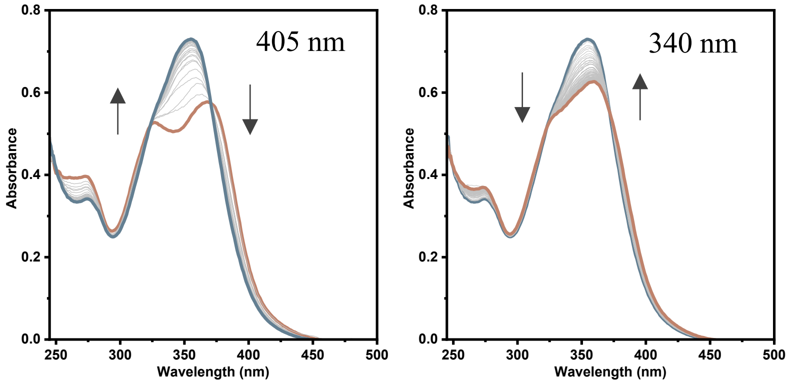

Figure S25. Conversion of **Se-SC2**$_{\boldsymbol{Z}}$ (orange line) in water-saturated chloroform ($3.61 \cdot 10^{-5}$ M) to **Se-SC2**$_{\boldsymbol{E\text{-}PSS}}$ (blue line) followed over time under constant light irradiation (λ_{irr} = 405 nm) (left). Conversion of **Se-SC2**$_{\boldsymbol{E,PSS}}$ (blue line) in water-saturated chloroform ($3.61 \cdot 10^{-5}$ M) to **Se-**

SC2*Z-PSS* (orange line) followed over time under constant light irradiation (λ_{irr} = 340 nm) (right).

3.3. Photostationary States Determination

$CDCl_3$ was neutralized by stirring with basic aluminum oxide for 2 h, followed by filtration, and then stored over 3 Å molecular sieves to remove residual water. Photostationary state (PSS) distribution for the compounds was determined by ^1H NMR spectroscopy. Solutions in neutralized $CDCl_3$ were degassed with argon for 5 min and subsequently irradiated at room temperature with an LEDs corresponding to the λ_{max} of each photoswitch. Aliquots were taken at defined time intervals and analyzed by ^1H NMR until a constant isomeric ratio was established.

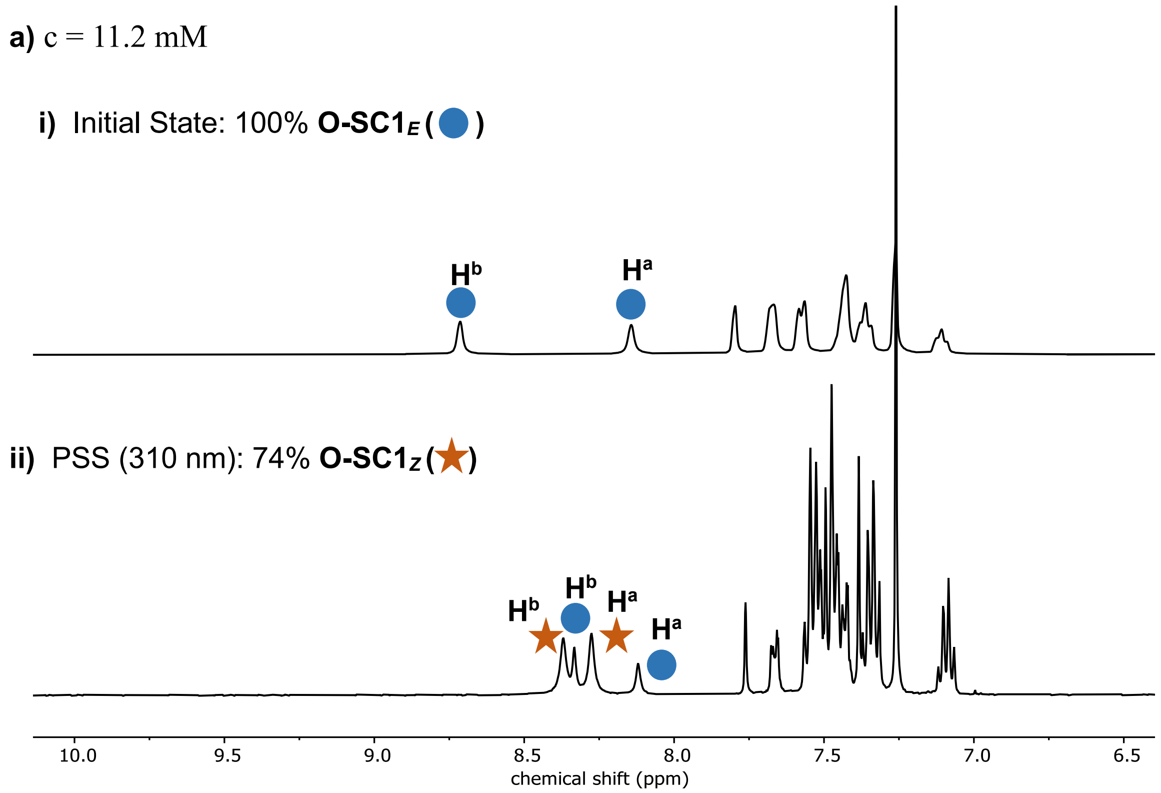

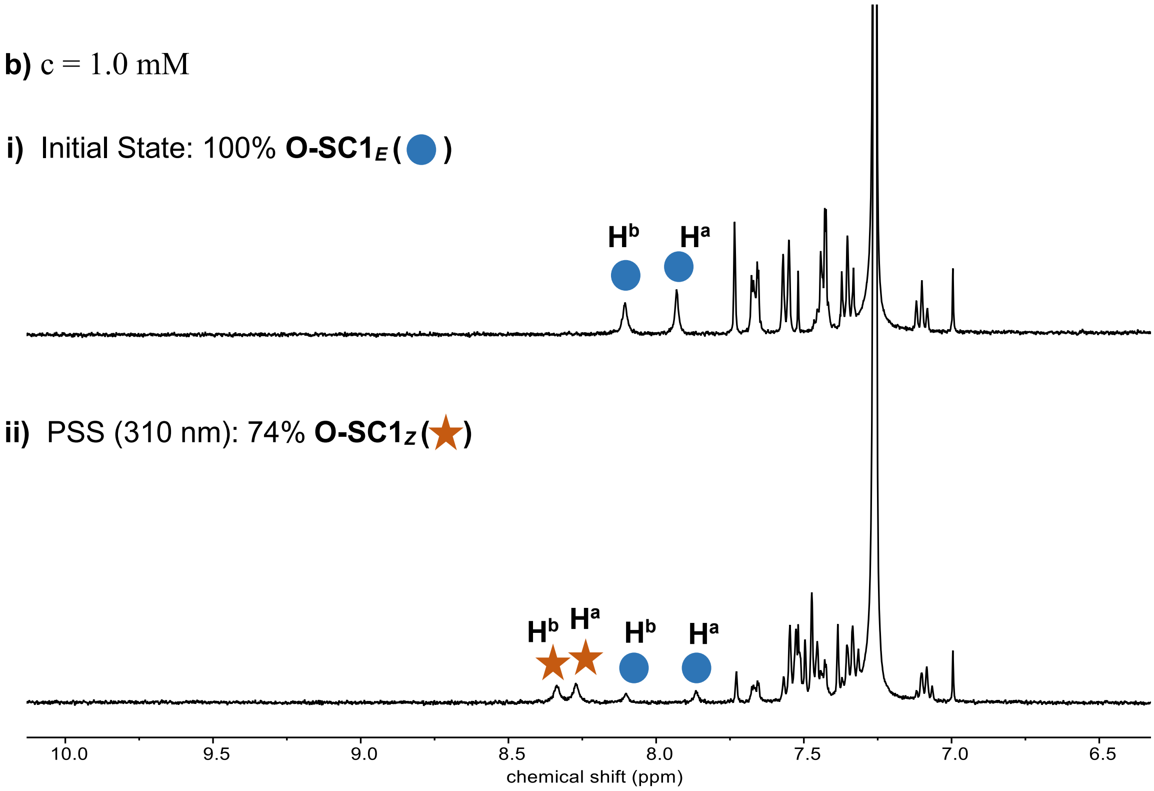

Figure S26. ^{1}H NMR spectra (400 MHz, $CDCl_3$, 25 °C) displaying the photoisomerization of **a) O-SC1** (c = 11.2 mM) **i)** before, and **ii)** after 310 nm irradiation to reach PSS. **b) O-SC1** (c = 1.0 mM) **i)** before, and **ii)** after 310 nm irradiation to reach PSS .

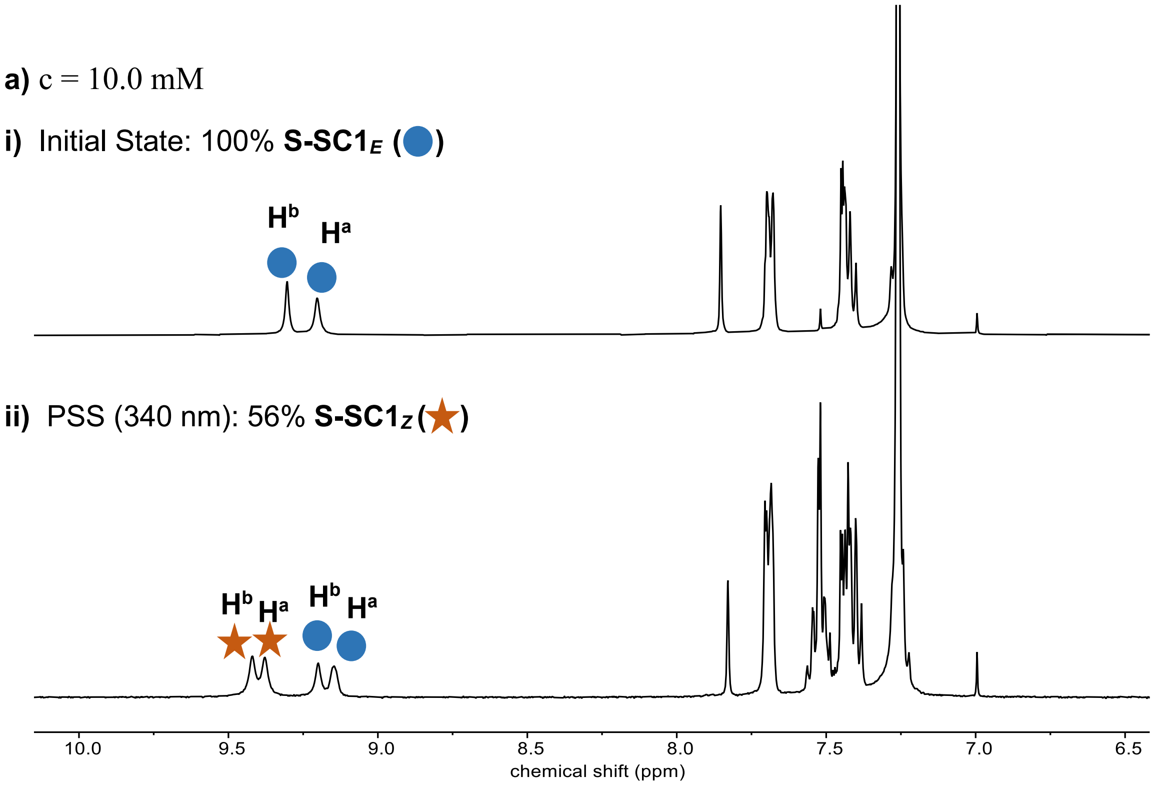

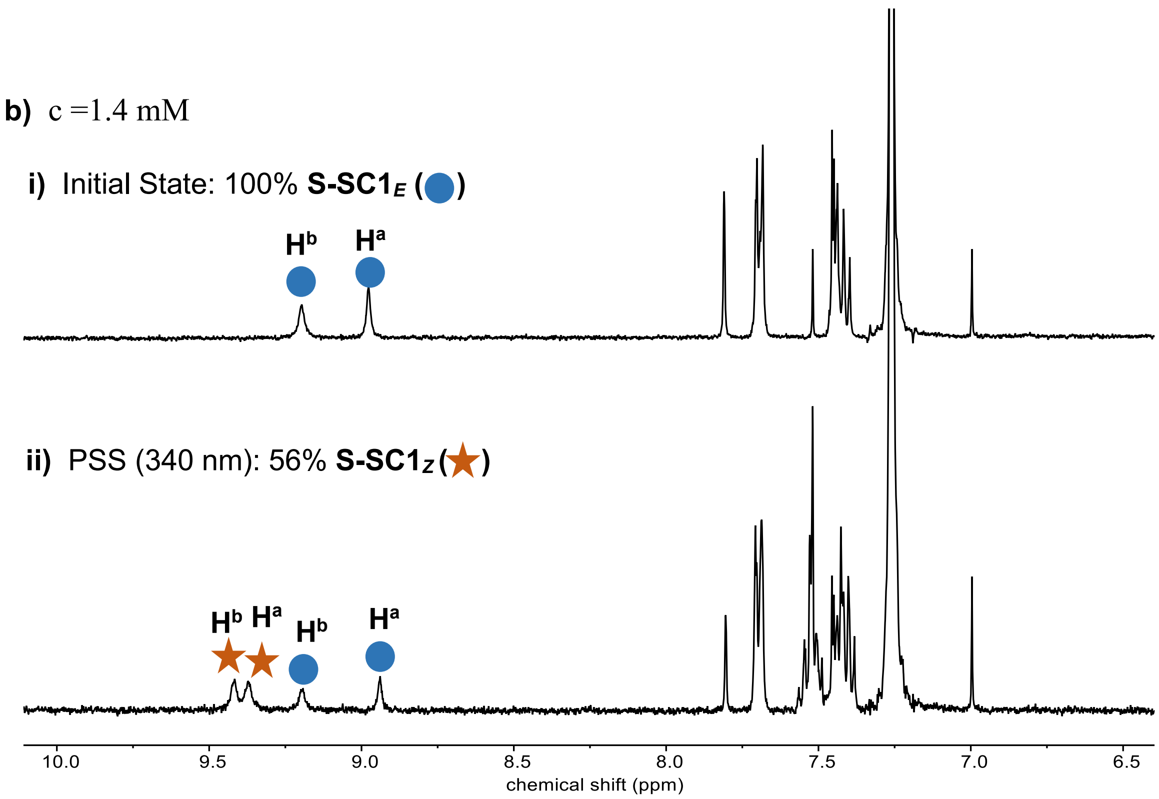

Figure S27. ^{1}H NMR spectra (400 MHz, $CDCl_3$, 25 °C) displaying the photoisomerization of **a) S-SC1** (c = 10.0 mM) **i)** before, and **ii)** after 340 nm irradiation to reach PSS. **b) S-SC1** (c =1.4 mM) **i)** before, and **ii)** after 340 nm irradiation to reach PSS.

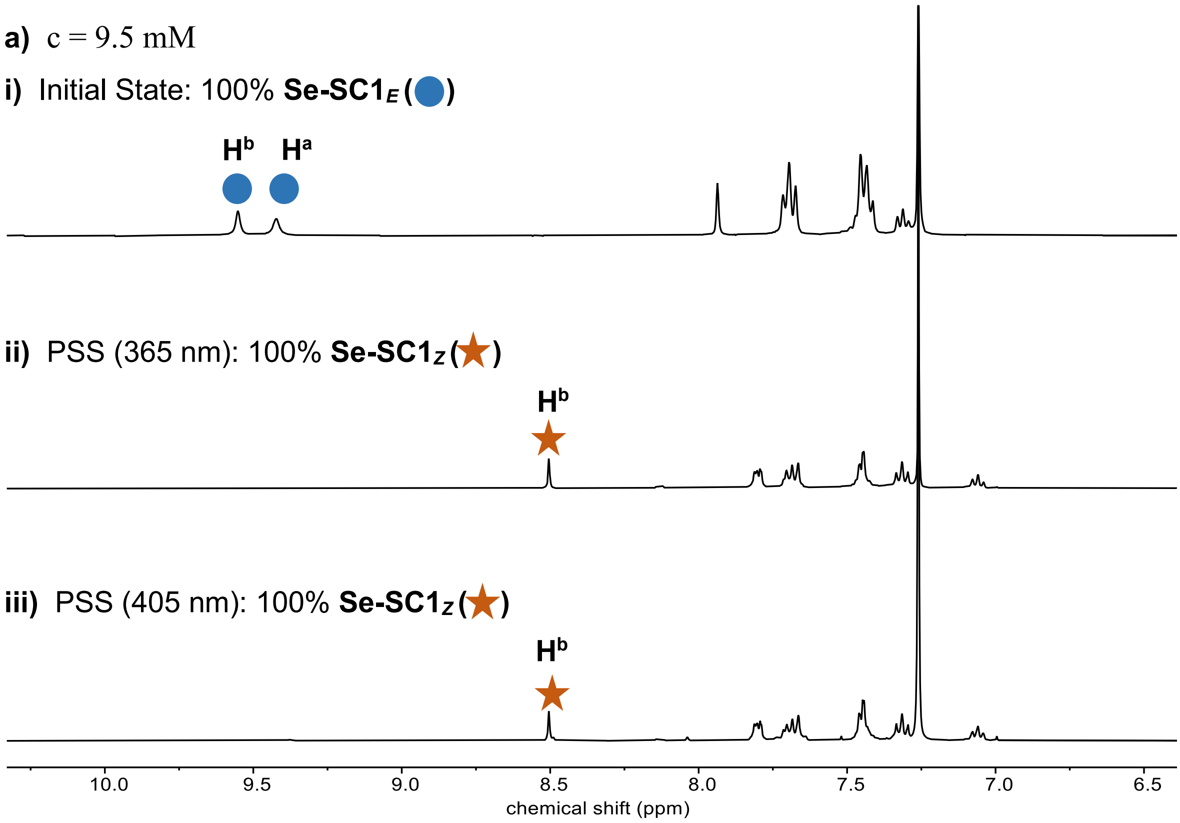

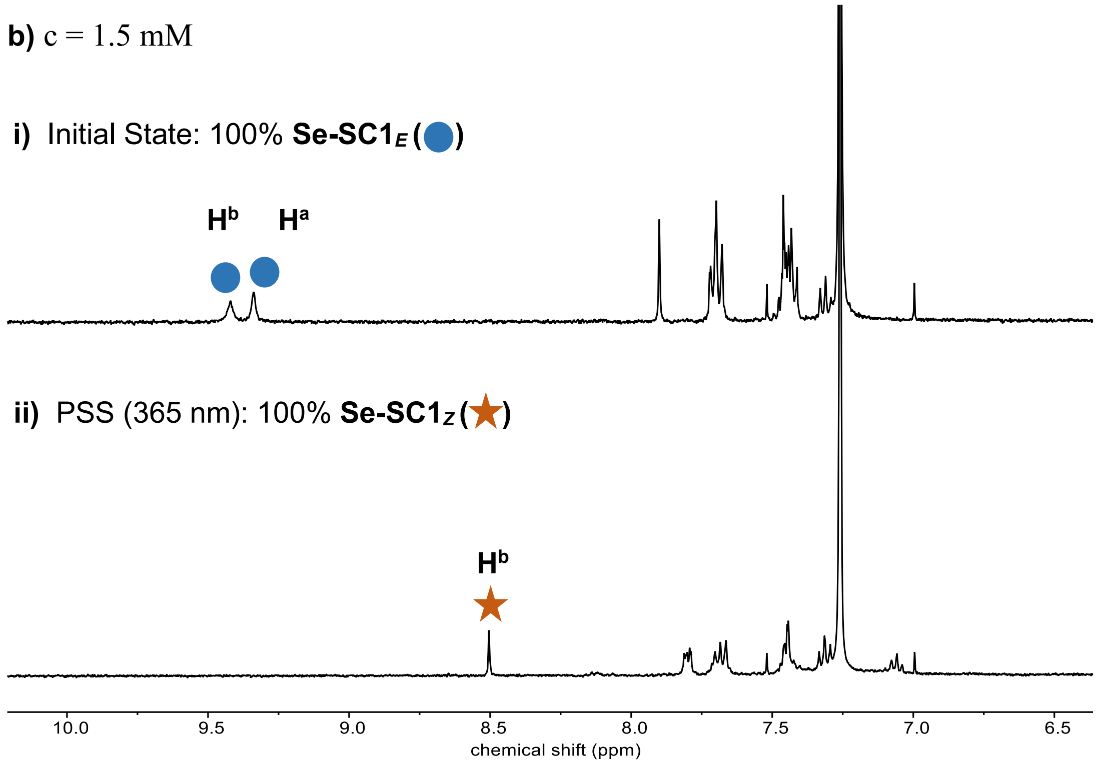

Figure S28. ^{1}H NMR spectra (400 MHz, $CDCl_3$, 25 °C) displaying the photoisomerization of **a) Se-SC1** (c = 9.5 mM) **i)** before, **ii)** after 365 nm, and **iii)** after 405 nm irradiation to reach PSS. **b) Se-SC1** (c = 1.5 mM) **i)** before, **ii)** after 365 nm irradiation to reach PSS.

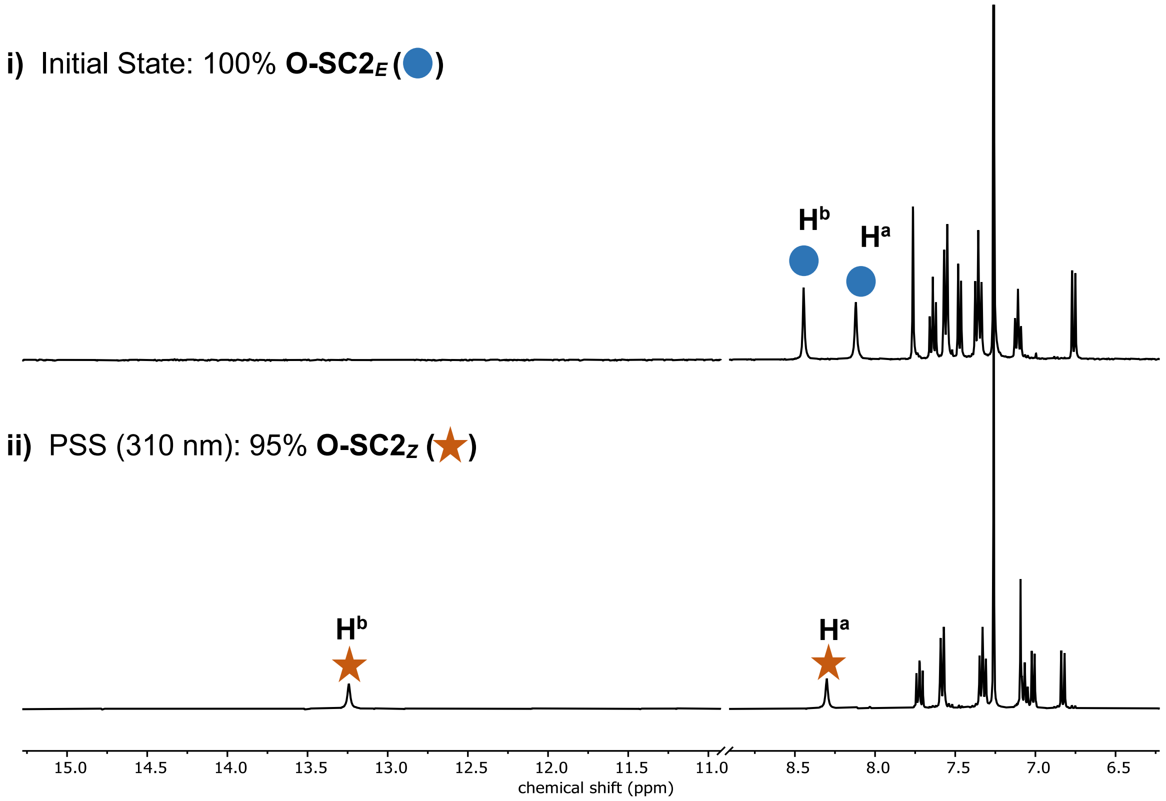

Figure S29. ^{1}H NMR spectra (400 MHz, $CDCl_3$, 25 °C) displaying the photoisomerization of **O-SC2** (c = 10.0 mM) **i)** before, and **ii)** after 310 nm irradiation to reach PSS.

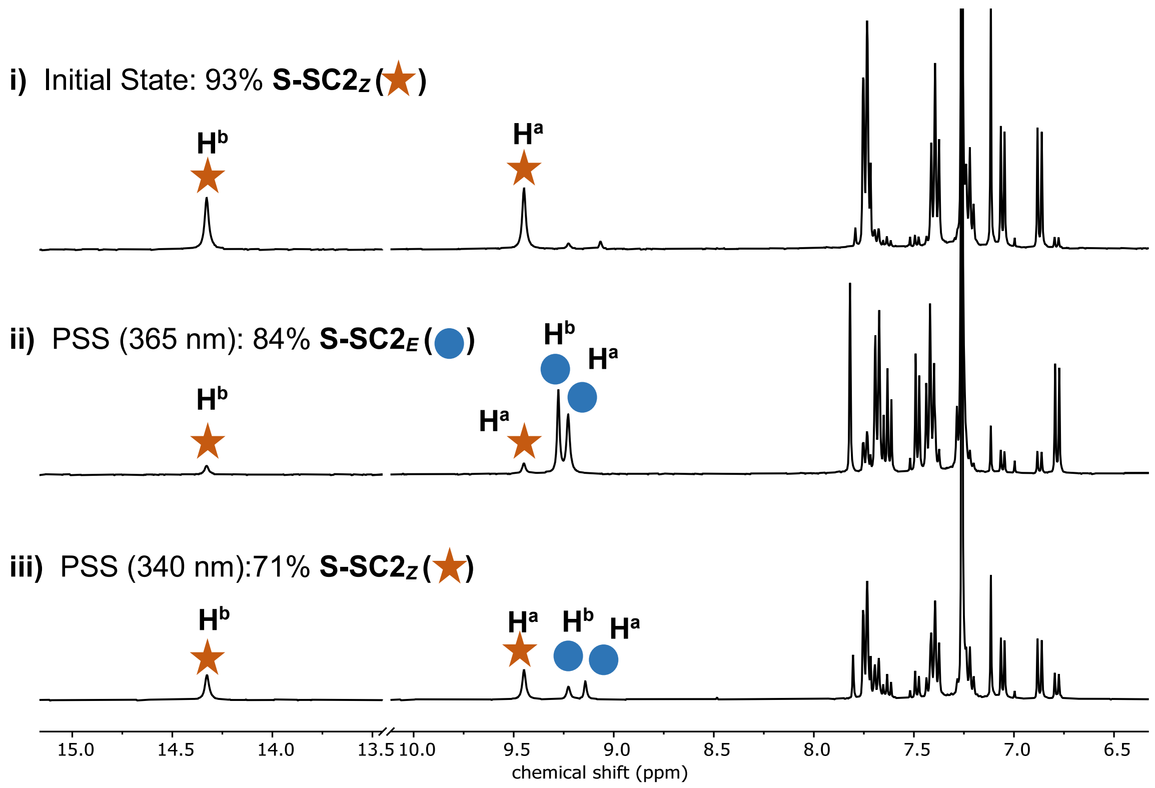

Figure S30. ^{1}H NMR spectra (400 MHz, $CDCl_3$, 25 °C) displaying the photoisomerization of **S-SC2** (c = 10.1 mM) **i)** before irradiation, **ii)** after 365 nm irradiation and **iii)** 340 nm irradiation to reach PSS.

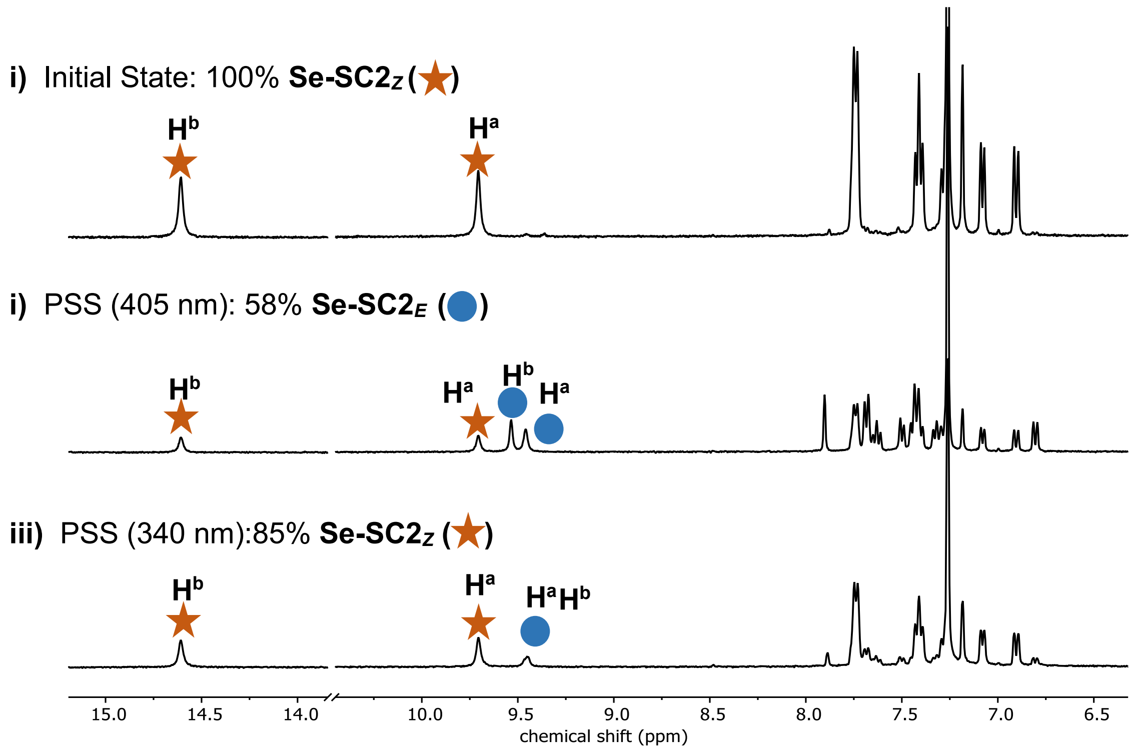

Figure S31. ^{1}H NMR spectra (400 MHz, $CDCl_3$, 25 °C) displaying the photoisomerization of **Se-SC2** (c = 8.2 mM) **i)** before irradiation, **ii)** after 405 nm irradiation and **iii)** 340 nm irradiation to reach PSS.

Table S2. Quantitative comparison of semicarbazone photoswitches.

Photoswitch	ε(*E*) [$M^{-1}cm^{-1}$]	$λ_{max}$(*E*) [nm]	$λ_{max}$(PSS) [nm][a]	$Δλ_{max}$ [nm][b]	$t_{1/2}$	*E*:*Z* ratio at PSS ($λ_{irr}$)
**O-SC1**	21,000	294	283	−11	26.5 min	26:74 (310 nm)
**S-SC1**	33,000	321	300	−21	29.5 min	44:56 (340 nm)
**Se-SC1**	26,000	340	330	−10	17.4 min	0:100 (365 nm)<br>0:100 (405 nm)
**O-SC2**	21,000	315	337	22	stable	5:95 (310 nm)
**S-SC2**	n.d.	335	312	27	stable	84:16 (365 nm)<br>29:71 (340 nm)
**Se-SC2**	26,000	353	318	15	stable	58:42 (405 nm)<br>15:85 (340 nm)

3.4. Fatigue Resistance

The fatigue resistance of the photoswitches was evaluated by repeated photoswitching cycles monitored by UV–vis spectroscopy in water-saturated chloroform.

For Series 1, solutions were irradiated at the wavelength corresponding to their respective $λ_{max}$ until the photostationary state was reached. After recording the UV–vis spectrum, the samples were kept in the dark at 20 °C to allow thermal back-relaxation to the initial isomeric state. This irradiation–relaxation sequence was repeated for several cycles, and the changes in absorbance at the characteristic band of the switched isomer were used to follow the switching amplitude.

For Series-2, solutions of corresponding photoswitches in water-saturated chloroform solution were irradiated to reach photostationary state, followed by irradiation at a second wavelength to induce photoisomerization in the reverse direction. UV–vis spectra were recorded after each irradiation step, and the absorbance changes at the diagnostic wavelength were monitored over multiple cycles to assess fatigue resistance.

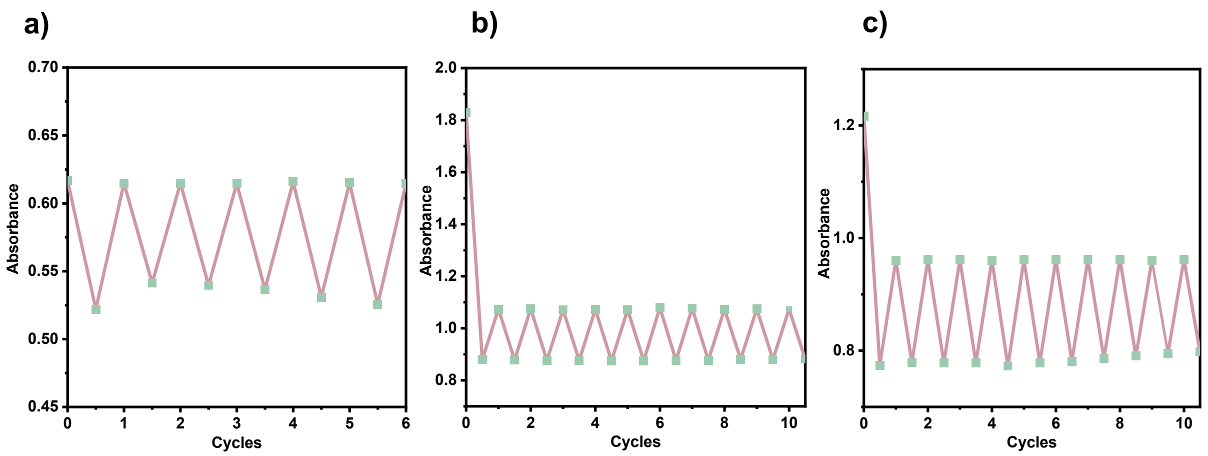

Figure S32. Change in absorbance at λ_{max} (*E*) during photoswitching cycles of **a) O-SC1** (c = 2.99·10^{-5} M), **b) S-SC1** (c = 5.49 ·10^{-5} M), and **c) Se-SC1** (c = 4.65 ·10^{-5} M) based on alternating irradiation with 310 nm, 340 nm LEDs (respectively) until reaching the certain point during thermal back-relaxation.

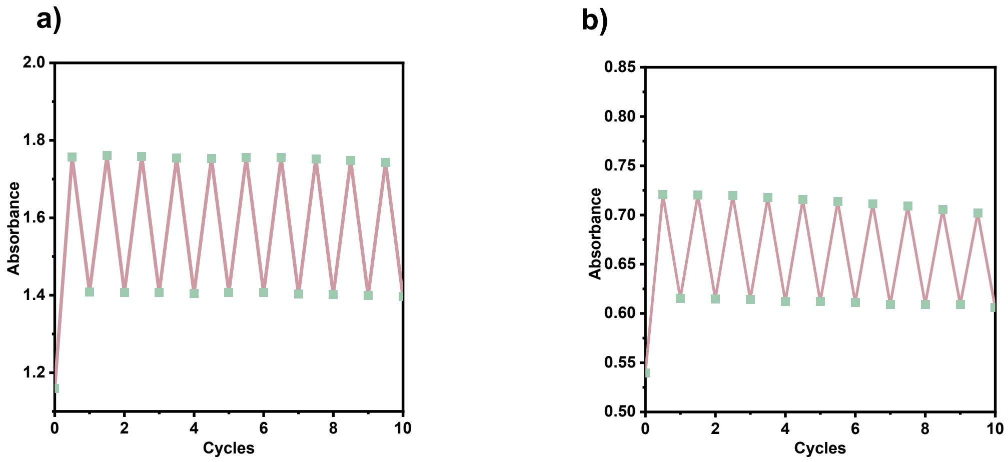

Figure S33. Change in absorbance at λ_{max} (*Z*) during photoswitching cycles of **a) S-SC2** (c = 4.31·10^{-5} M) based on alternating irradiation with 365 nm and 340 nm LEDs until reaching the certain points in both directions **b) Se-SC2** (c = 3.61 ·10^{-5} M) based on alternating irradiation with 405 nm and 340 nm LEDs until reaching the certain points in both directions.

3.5. Quantum Yields

4,4′-Dimethylazobenzene (DMAB) was utilized as a chemical actinometer to measure the incident photon flux.[7] To this end, 3 mL of DMAB solution in chloroform (c = 5.42·10^{-4} M) was placed in a 1 cm pathlength cuvette with a magnetic stirrer. The absorption spectrum was recorded before the irradiation with 310 nm (400 mA), 340 nm (500 mA) and 365 nm (100 mA) LEDs. The sample was irradiated under continuous stirring, and absorbance change at 440 nm over time was monitored, which was then plotted and fitted to a linear equation

$$y = mx$$

Which physically corresponds to

$$A = A_0 - \left(\frac{q_0 \cdot \Phi_{EZ} \cdot \Delta\varepsilon_{\lambda_x}}{V}\right) t$$

q_0 was determined according to the equation below:

$$q_0 = \frac{m \cdot V \cdot N_A}{\Phi_{EZ} \cdot \Delta\varepsilon_{\lambda_x}}$$

For a further simplification of the data treatment, the constant values V, $\Phi_{E\to Z}$ and $\Delta\varepsilon$ at 440 nm (ε_E = 798 M^{-1} cm^{-1}, ε_Z = 1747 M^{-1} cm^{-1}) can be gathered in a parameter X (310 nm = $1.86{\cdot}10^{-5}$, 340 nm = $1.58{\cdot}10^{-5}$ mol, and 340 nm = $1.76{\cdot}10^{-5}$), thus:

$$q_0 = m \cdot X \cdot N_A$$

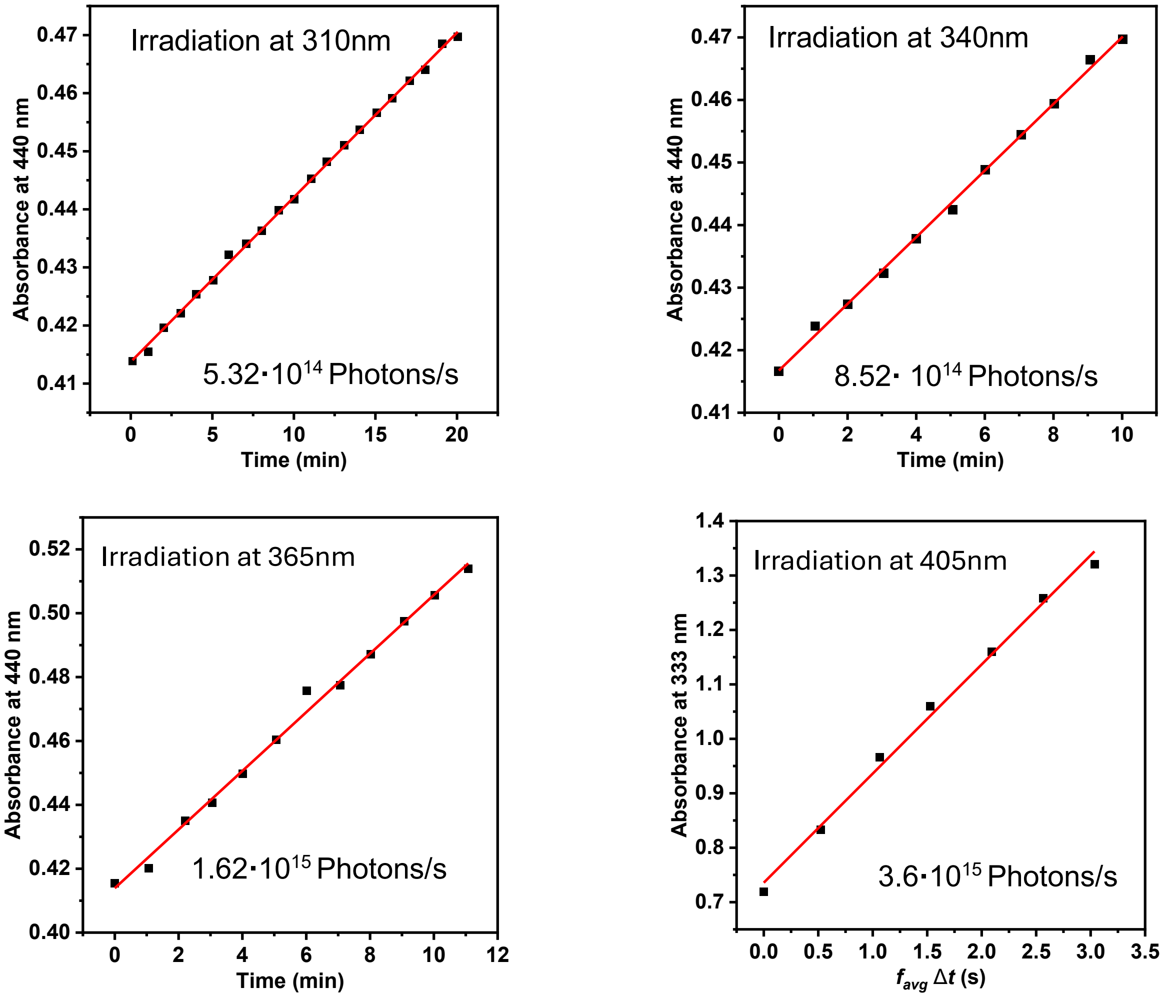

Figure S34. Determination of photon flux from 310 nm (400 nm), 340 nm (500 mA) nm, 365 nm (100 mA), 405 nm (300 mA) LEDs.

Quantum yields were determined by fitting the time-dependent absorbance data using the software provided by Stranius and Börjesson.[8] The absorbance change over time was fitted to the following equation;

$$\frac{d[A]}{dt} = -\frac{\Phi_A \cdot q_0 \cdot \beta_A(t)}{N_A \cdot V} + \frac{\Phi_B \cdot q_0 \cdot \beta_B(t)}{N_A \cdot V} + k_{t,B\rightarrow A}[B]$$

Where Φ is the quantum yield of photoisomerization of a given species, q_0 is the photon flux, β is the fraction of photons absorbed by a given species, V is the volume of sample, N_A is Avogadro's numbers, k_t is the rate of the spontaneous back conversion.

The extinction coefficient of the pure *E*-isomer of the corresponding semicarbazone was determined through a combination of ^{1}H NMR and UV-vis spectroscopy. The sample in $CDCl_3$ was irradiated with the aforementioned LEDs. Simultaneous NMR and UV-vis studies were then performed to determine both the composition and the absorbance of the isomers at a given time.

a)

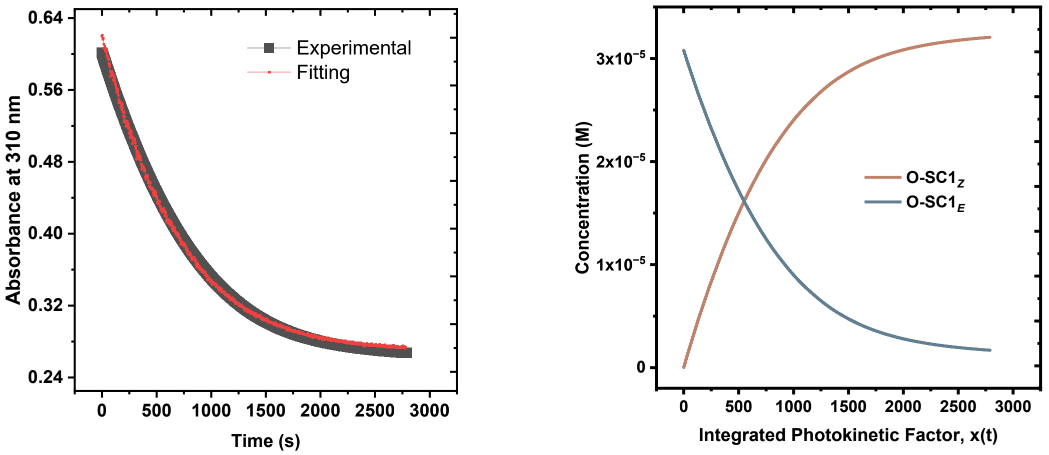

b)

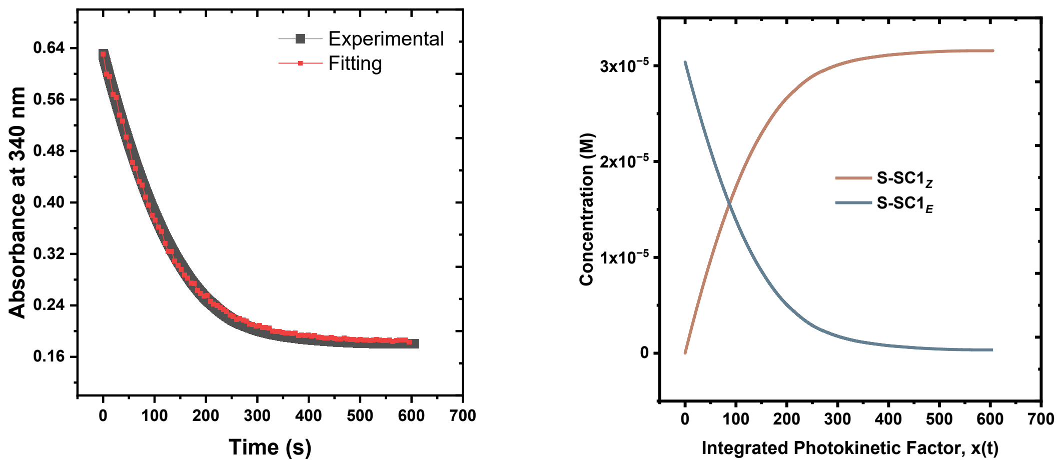

c)

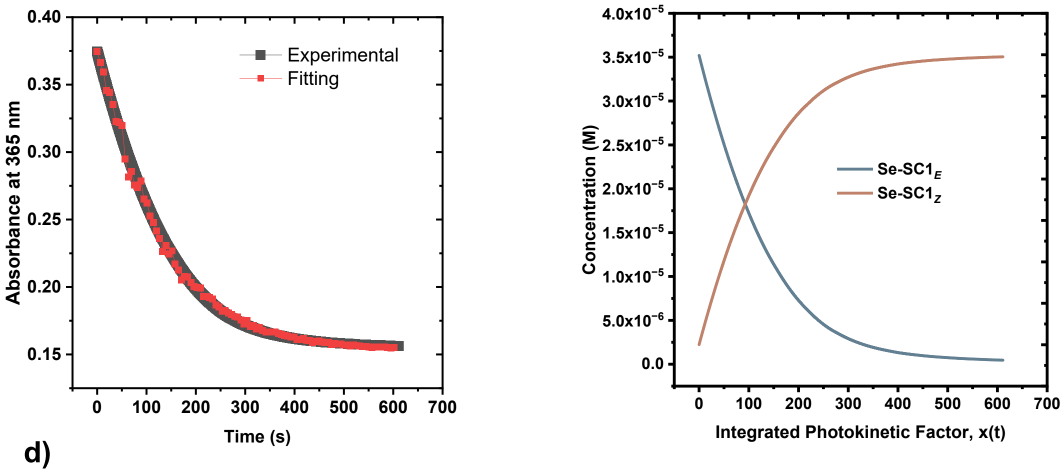

d)

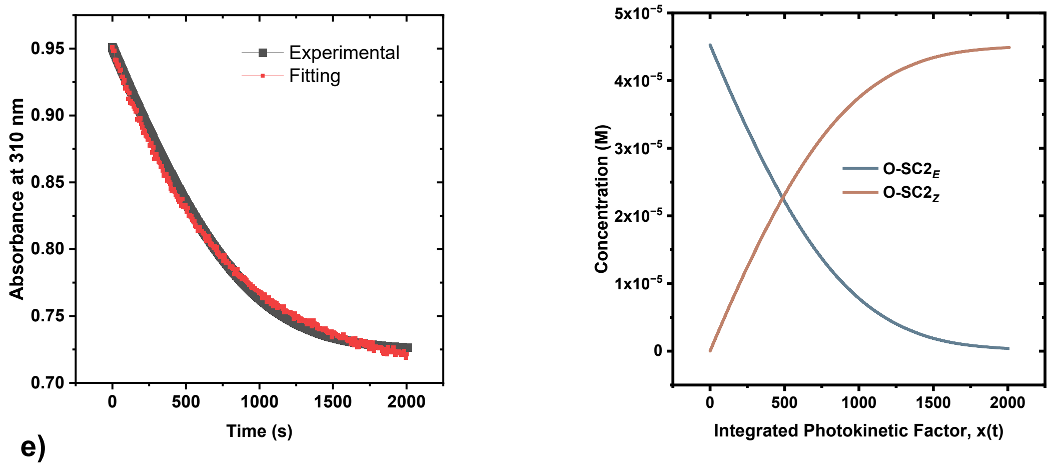

e)

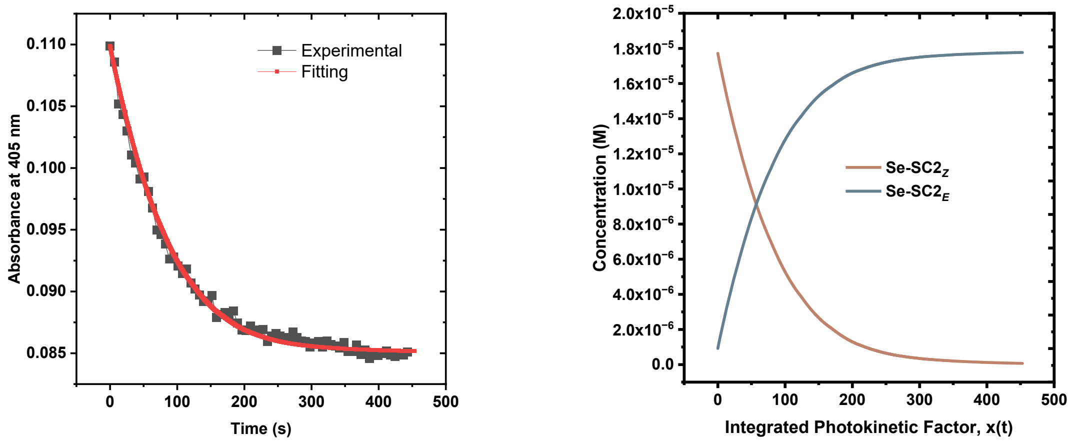

Figure S32. The plot of the change in absorbance at irradiation wavelength as a function of irradiation time with 310 nm, 340 nm, and 365 nm LEDs (right) and the integrated photokinetic factor, (x)t plotted against the concentration of *E*- and *Z*-isomers (left) of **a) O-SC1**, **b) S-SC1**, **c) Se-SC1**, **d) O-SC2**, and **e) Se-SC2.** The quantum yield calculations were performed using a previously published method.[8]

Table S2. The overview of extinction coefficients of *E* and *Z*-isomers and the quantum yield of photoisomerization at 310 nm, 340 nm, 365 nm and 405 nm. The data are obtained from UV-vis measurements performed in chloroform at 20 °C.

Photoswitch	$\mathcal{E}_E$ [$M^{-1}cm^{-1}$]	$\mathcal{E}_Z$ [$M^{-1}cm^{-1}$]	k_t [s^{-1}]	$\boldsymbol{\Phi}$ (%)
**O-SC1**	14,131	3,861	$4.3\cdot10^{-4}$	19 (*E*→*Z* @ 310 nm)
**S-SC1**	23,944	5,095	$3.8\cdot10^{-4}$	33 (*E*→*Z* @ 340 nm)
**Se-SC1**	10,642	4,359	$6.6\cdot10^{-4}$	44 (*E*→*Z* @ 365 nm)
**O-SC2**	21,000	16,000	0	20 (*E*→*Z* @ 310 nm)
**Se-SC2**	6,200	4,800	0	45 (*Z*→*E* @ 405 nm)

3.6. Self-association studies of the X-SC1 series

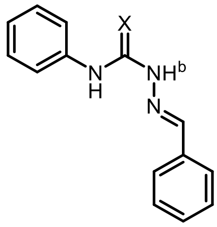

Self-association studies on the Series-1 photoswitches **O-SC1**, **S-SC1**, and **Se-SC1** were performed in $CDCl_3$ from sealed ampoules at room temperature. Solutions of each compound were prepared at different concentrations, and the corresponding ^{1}H NMR spectra were recorded. Upon dilution, pronounced concentration-dependent shifts of the H^b signal were observed for all three photoswitches (**Figures S36–S38**). These changes are indicative of supramolecular self-association in solution and, by analogy to previously reported thiosemicarbazone systems, are consistent with aggregation driven by intermolecular N–H···X=C hydrogen bonding.[9] To obtain a quantitative measure of the relative self-association tendency, the concentration-dependent chemical shifts were fitted with a simple monomer–dimer equilibrium model using the equation:

$$\boldsymbol{\delta_{obs}} = \delta_m + (\delta_d - \delta_m)\frac{\sqrt{\mathbf{1+8}\boldsymbol{k_{dim}}[\mathbf{Conc}]}-\mathbf{1}}{\sqrt{\mathbf{1+8}\boldsymbol{k_{dim}}[\mathbf{Conc}]}+\mathbf{1}}$$

where δ_m and δ_d represent the ”true” chemical shifts of the monomer and the dimer, respectively. K_{dim} is the dimerization constant.

We note that this NMR analysis does not establish the aggregation stoichiometry: DFT calculations (Section 4.3 and 4.4) indicate that the most stable aggregate is in fact a π-π-stacked,

hydrogen-bonded tetramer. Thus, K_{dim} should be regarded as an effective self-association constant that captures the overall strength of aggregation over the concentration range studied.

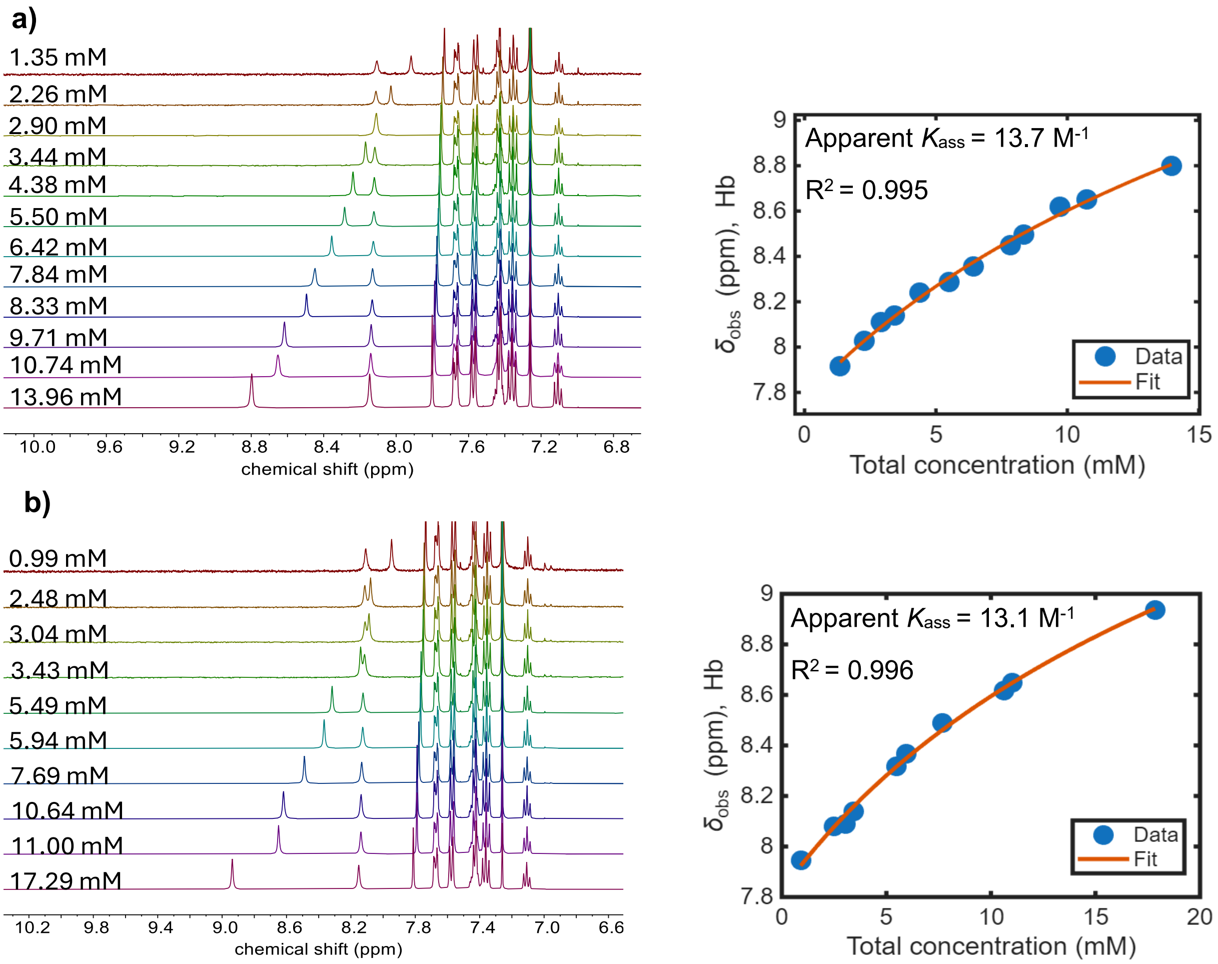

Figure S33. ^{1}H NMR (400 MHz, 25 ºC) spectra of **O-SC1** in $CDCl_3$ at different concentrations (left) and graph of chemical shift of H^b vs. concentration (right). **a)** experiment-1 **b)** experiment-2.

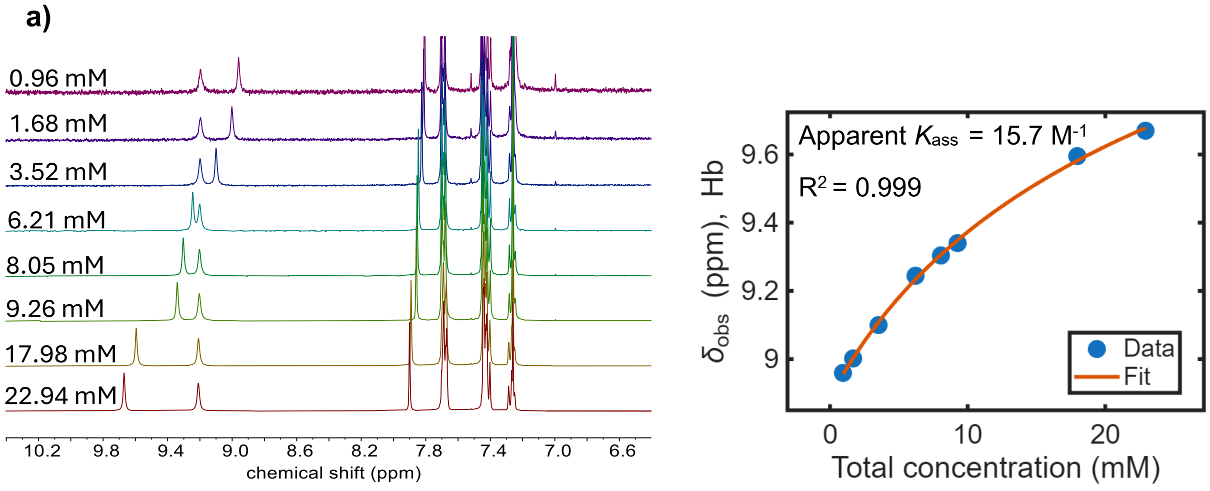

b)

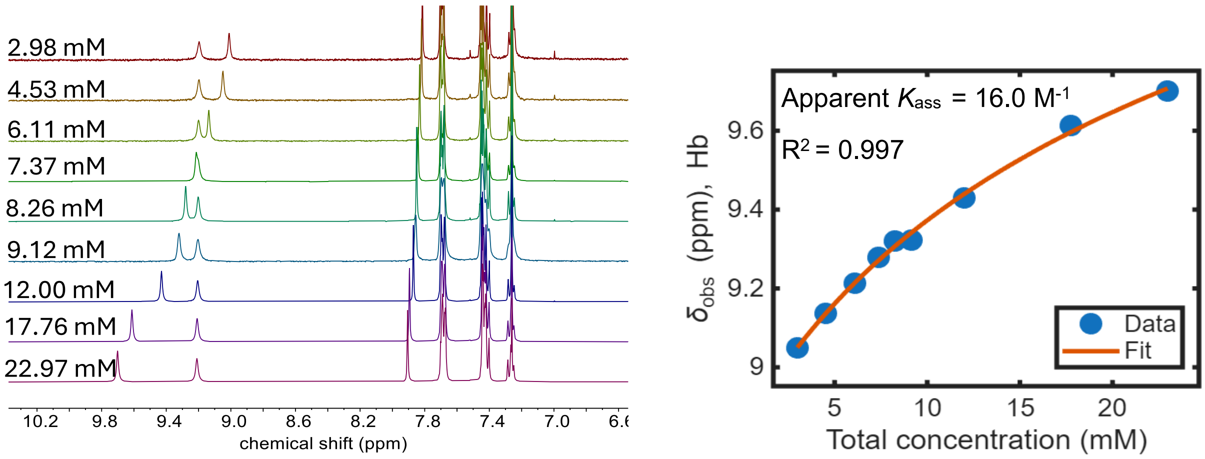

Figure S34. ^{1}H NMR (400 MHz, 25 ºC) spectra of **S-SC1** in CDCl₃ at different concentrations (left) and graph of chemical shift of H[b] vs. concentration (right). **a)** experiment-1 **b)** experiment-2.

a)

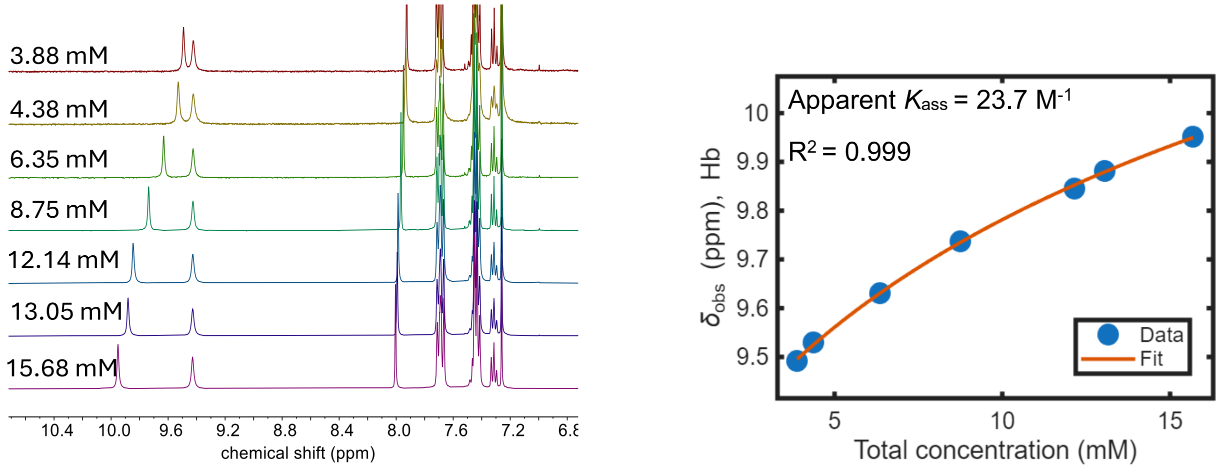

b)

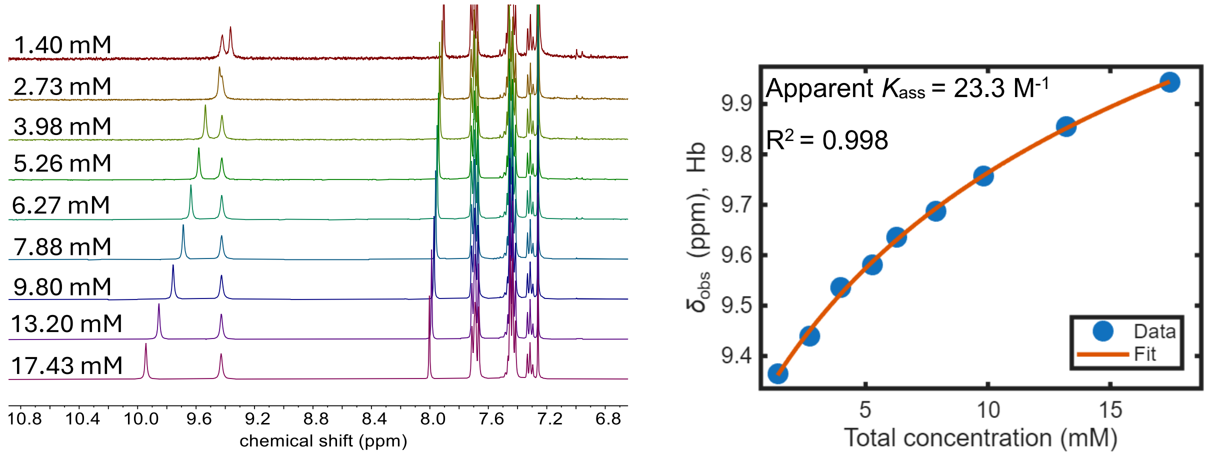

Figure S35. ^{1}H NMR (400 MHz, 25 ºC) spectra of **Se-SC1** in CDCl₃ at different concentrations (left) and graph of chemical shift of H[b] vs. concentration (right). **a)** experiment-1 **b)** experiment-2.

3.7. Stepwise Aggregate Deactivation Studies on X-SC1

$CDCl_3$ was passed through basic aluminum oxide to remove acidic stabilizers and used immediately after treatment. A total volume of 3.0 mL of a three-component solution of **O-SC1*E***, **S-SC1*E***, and **Se-SC1*E*** was prepared in the pretreated $CDCl_3$ at final concentrations of 7 mM, 14 mM, and 10 mM, respectively, chosen such that the N–H resonances of the three components appear with comparable but distinct intensities in the ^{1}H NMR spectrum to facilitate assignment. The solution was degassed by briefly sparging with an argon flow (1–2 min) and then subjected to illumination. Aliquots were taken at defined time intervals and analyzed by ^{1}H NMR to monitor the spectral changes during irradiation. Upon reaching the PSS at the first wavelength, irradiation was continued at the next wavelength (as indicated in **Figure S36**) and the same sampling and ^{1}H NMR analysis procedure was repeated.

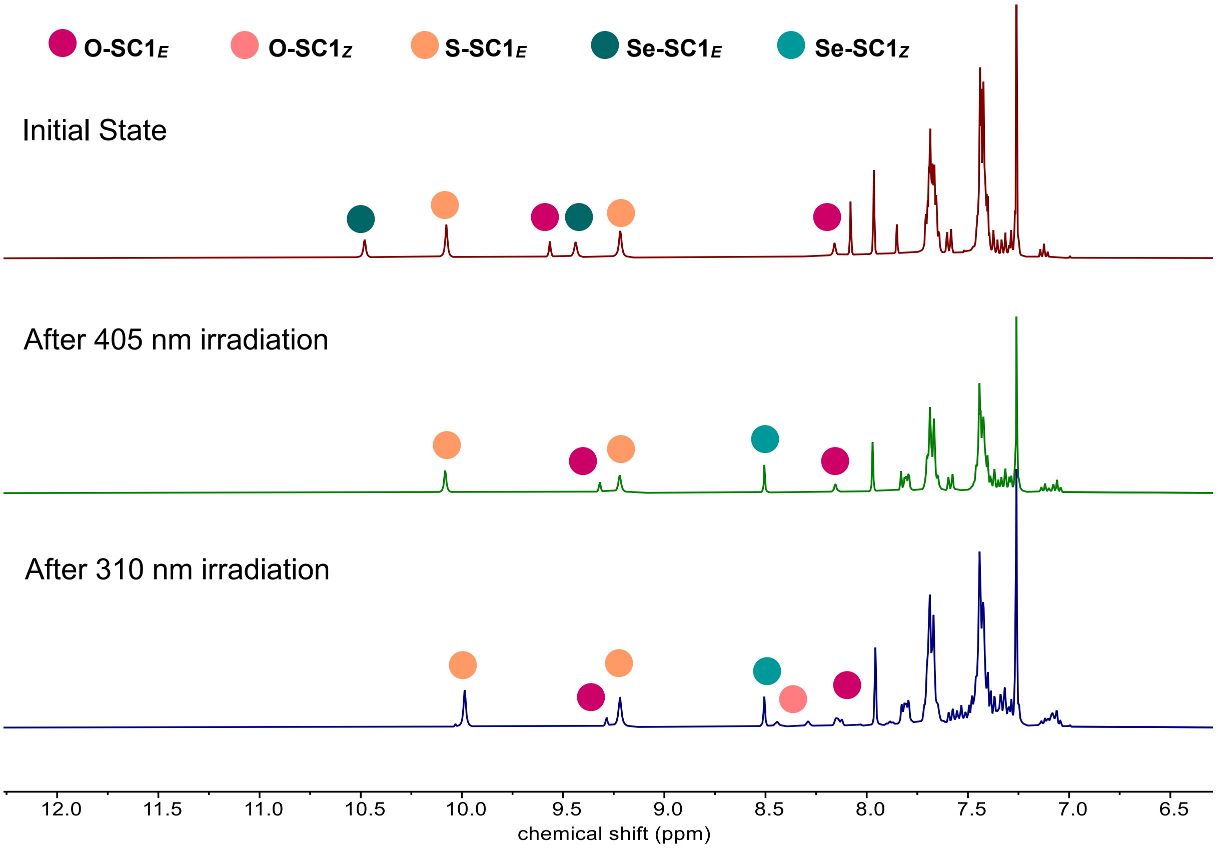

Figure S36. Sequential irradiation experiment of a three-component mixture of **O-SC1*E***, **S-SC1*E***, and **Se-SC1*E*** in $CDCl_3$ monitored by ^{1}H NMR spectroscopy. The sample was irradiated at 405 nm for 4 hours until **Se-SC1*E*** was quantitatively converted to **Se-SC1*z*** , followed by irradiation at 310 nm for 8 hours until the PSS for the photoisomerization of **O-*SC1E*** (PSS = 50%) was reached.

4. Computational Investigations

All computations were performed using the 2024.102 release of the Amsterdam Density Functional (ADF) program within the Amsterdam Modeling Suite.[10,11] Geometry optimizations and energy evaluations were carried out at the BLYP level of theory, which combines the generalized gradient approximation (GGA) exchange functional by Becke[12] with the correlation functional of Lee, Yang, and Parr (LYP).[13] To account for dispersion interactions, the DFT-D3(BJ) correction developed by Grimme and co-workers[14] was employed, incorporating the Becke–Johnson damping scheme.[15] Scalar relativistic effects were included using the zeroth-order regular approximation (ZORA).[16] This computational setup, referred to as ZORA-BLYP-D3(BJ)/TZ2P, has been shown to reliably capture weak intermolecular interactions.[17,18]

A triple-ζ quality TZ2P basis set consisting of uncontracted, relativistically optimized Slater-type orbitals (STOs) was used without applying a frozen-core approximation. The basis set was augmented with polarization functions: p and d functions for hydrogen, and d and f functions for nitrogen, carbon, oxygen, sulfur, and selenium.[19] The BLYP-D3(BJ)/TZ2P level of theory has been demonstrated to yield a basis set superposition error (BSSE) of only a few tenths of a kcal mol^{-1} in hydrogen-bonded systems, which is negligible for comparative purposes.[20] Electron densities were fitted using the Zlm fitting method, and numerical integrations were conducted using a Becke-type grid, both specified with the "VeryGood" quality setting. [21,22] Molecular structures were visualized with CYLview2.0.[23]

4.1 Conformer computations

X-SC1$_{\boldsymbol{E/Z}}$ monomers were obtained by isolating a single monomer from the crystal structure of **S-SC1**. The sulfur atoms were consequentially replaced by oxygen and selenium, yielding **O-SC1** and **Se-SC1** monomers. From here, conformational searches of **X-SC1** compounds were performed using CREST[24] at a temperature of 25 °C (298 Kelvin; standard conditions). Conformers with an energy of +2.0 kcal mol^{-1} relative to the lowest one were neglected and duplicates were removed based on their RSMD (<0.05). The filtered conformers were optimized at the above DFT level. The conformation searches identified the *E*-isomer as a global minimum and the *Z*-isomer as a local minimum for **X-SC1** (see **Table S10** for coordinates and energies).

To confirm that the conformers are in their lowest energy conformation, frequency analyses were carried out with zero imaginary frequencies for equilibrium geometries.[25,26] This also allowed for calculating thermostatistical corrections for determining the enthalpy H, entropy S,

and Gibbs free energy of the stationary points for which the default implementation in ADF was considered.

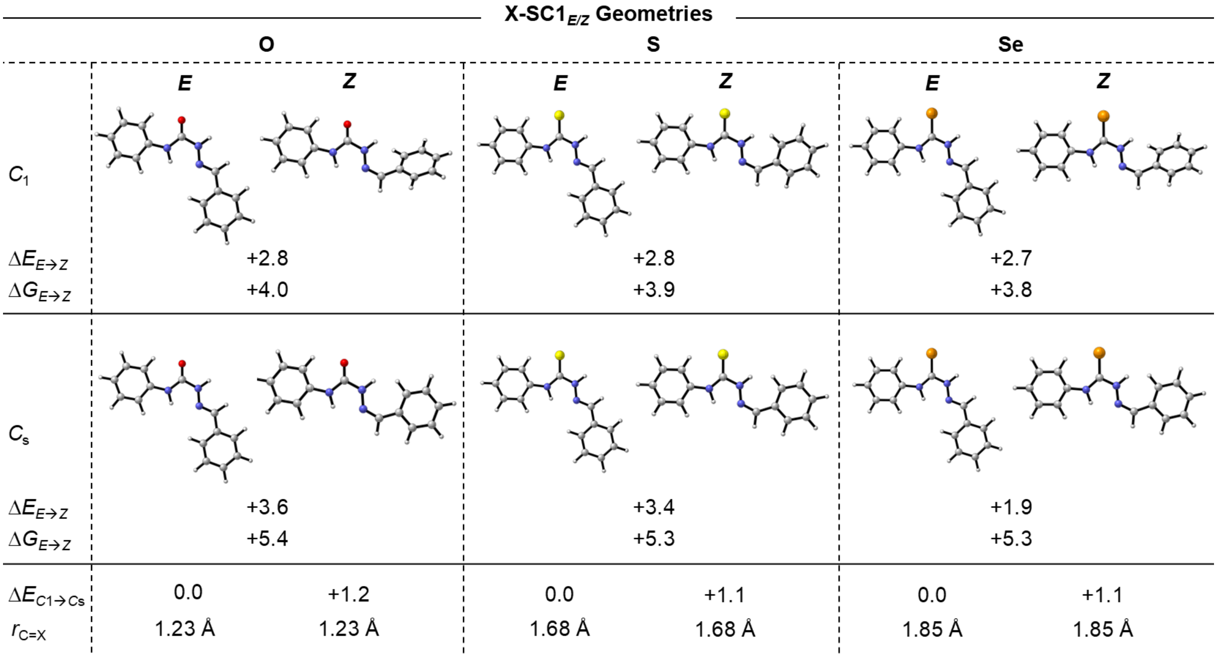

Figure S37. Geometries and (free) energy differences of **X-SC1 isomers** (*E* vs *Z*) with X = O, S, or Se, computed at ZORA-BLYP-D3(BJ)/TZ2P. Bond lengths of the C=X bond are shown (in Å) and the energy terms are defined as D$E_{E\rightarrow Z} = E_Z - E_E$ and D$E_{C1\rightarrow Cs} = E_{Cs} - E_{C1}$ (in kcal mol^{-1}).

4.2 Excitations and time-dependent density functional theory (TD-DFT) computations

The excitation energies were calculated using the linear response TD-DFT as implemented in ADF.[27,28] Excitations were computed for the p electronic system which involved singlet-singlet and singlet-triplet transitions. The long-range separated exchange-correlation functional CAMY-B3LYP[29] was considered with a TZ2P basis set (no frozen core) and the scalar ZORA for accounting for relativistic effects. In general, long-range separated functions perform well for excitations with a charge-transfer nature because the associated correlation potential shows the correct asymptotic nature, leading to successful predictions of excitation energies.[30,31] The CAMY-B3LYP functional was chosen since it can accurately predict various types of excitations in organic dye molecules.[32] The default parameters of CAMY-B3LYP were used (as implemented in ADF), being g = 0.34, a = 0.19, and b = 0.46.

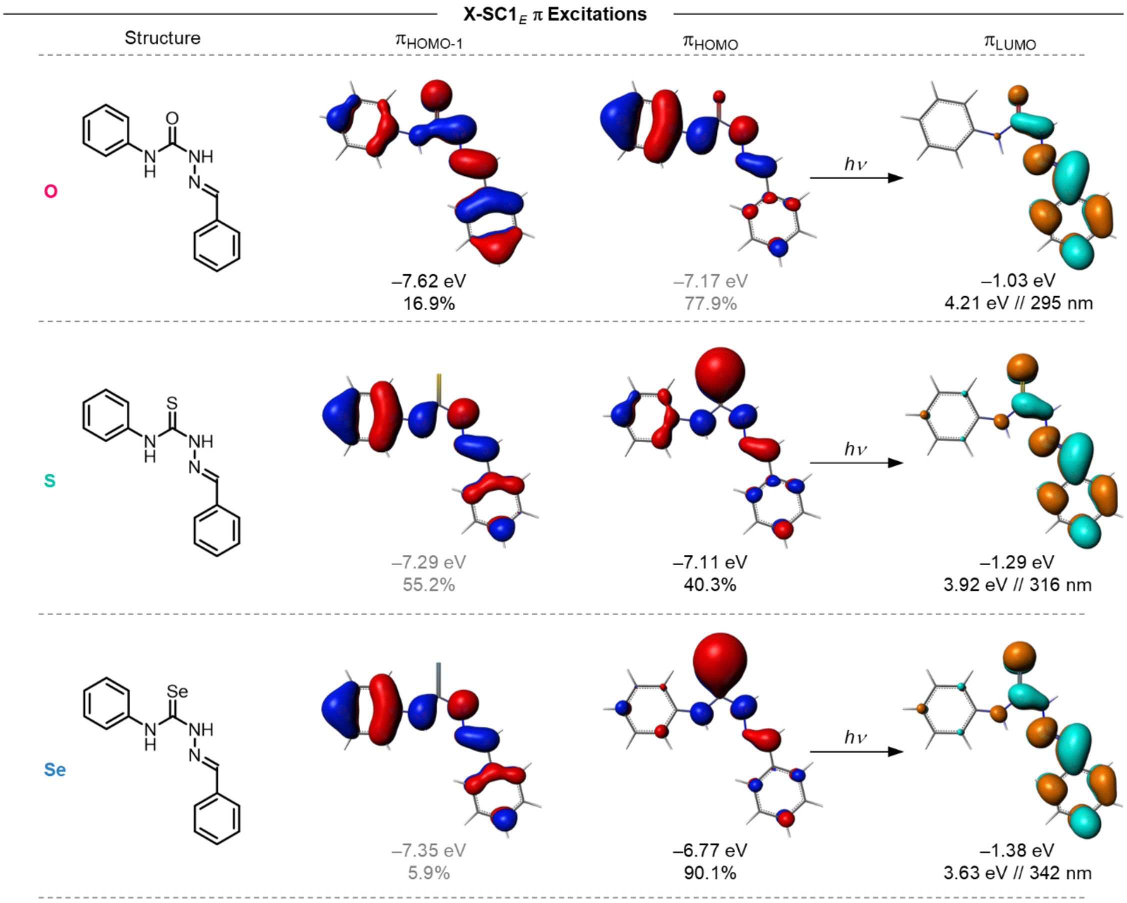

Figure S38. Molecular orbitals involved in singlet-singlet p excitations in **X-SC1*E*** compounds with **X** = O, S, or Se (plotted at isovalue ± 0.03). Associated orbital energies (in eV), contributions (in %), and excitation wavelengths (in eV and nm). The MOs with values in grey represent the orbitals with low or no contributions from the np$_z$ orbital of the chalcogen, computed at ZORA-CAMY-B3LYP/TZ2P//ZORA-BLYP-D3(BJ)/TZ2P.

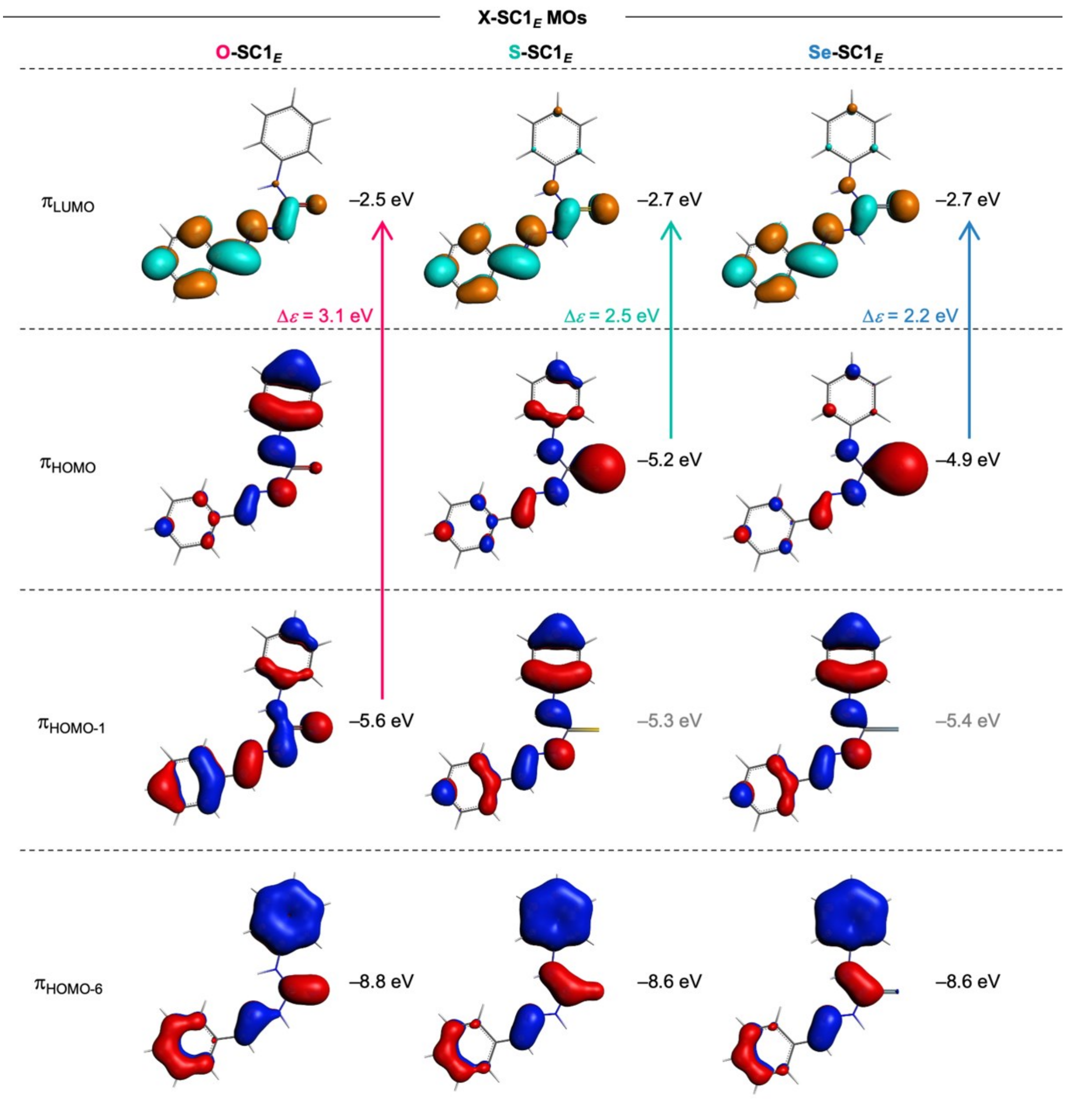

Figure S39. Molecular orbitals (MOs) of **X-SC1*E*** with **X** = O, S, or Se that are shown in **Figure 2**. The MOs with energies in grey denote the orbitals in which the np$_z$ orbital of the chalcogen does not contribute to, computed at ZORA-BLYP-D3(BJ)/TZ2P.

Table S3. Gross populations (in electrons), contributions (%) and overlaps (a.u.) of the fragment molecular orbitals that interact to form the π bond in the C=X double bond in **X-SC1*E*** with **X** = O, S, or Se, computed at ZORA-BLYP-D3(BJ)/TZ2P.

	**O-SC1*E***		**S-SC1E**		**Se-SC1*E***	
	SC1*E* π_{SOMO}	**O-** *2pz*	SC1*E* π_{SOMO}	**S-***3pz*	SC1*E* π_{SOMO}	**Se-** *4pz*
Gross Pop (electrons)	0.35	1.52	0.36	1.53	0.34	1.59
Electron-pair bond (singly occupied – singly occupied)						
S = ⟨SC1*E* π_{SOMO} | **X-** *n*pz⟩	0.19		0.17		0.16	
$S^2 * 10^3$	36		29		26	
Pauli repulsion (occupied – occupied)						
S = ⟨SC1*E* π_{HOMO-6} | **X-** *n*pz⟩	0.065		0.051		0.042	
$S^2 * 10^3$	4.2		2.6		1.8	

4.3 Bond energy analysis

The bonding process underlying tetramer formation was analyzed by considering four isolated **X-SC1*E*** monomers aggregating into a tetramer. The overall bonding energy associated with this process, Δ*E*, is defined as shown in Equation (1):

$$\Delta E = E_{\text{tetramer}} - \sum_{monomers} E_{\text{monomer}} \quad (1)$$

where E_{tetramer} denotes the energy of the tetramer and E_{monomer} the energy of each monomer in its equilibrium geometry. To gain mechanistic insight into the factors governing aggregation, Δ*E* was partitioned according to the activation strain model (ASM) formulated by Equation (2):[33–36]

$$\Delta E = \Delta E_{\text{strain}} + \Delta E_{\text{int}} \quad (2)$$

Here, the strain energy (ΔE_{strain}) represents the energetic cost of deforming each monomer from its equilibrium geometry to the geometry adopted in the tetramer. The interaction energy (ΔE_{int})

accounts for stabilizing interactions between deformed monomers. We can decompose the interaction energy further within the framework of the Kohn-Sham molecular orbital model (KS-MO), using the quantitative canonical energy decomposition analysis (EDA[37,38]). We identify the electrostatic interaction (ΔV_{elstat}), Pauli repulsion (ΔE_{Pauli}), orbital interactions (ΔE_{oi}), and an additional term that accounts for dispersion interactions ΔE_{disp}, as shown in Equation (3).

$$\Delta E_{int} = \Delta V_{elstat} + \Delta E_{Pauli} + \Delta E_{oi} + \Delta E_{disp} \quad (3)$$

The ΔV_{elstat} term denotes the quasi-classical Coulomb interaction between the unperturbed charge distributions of the deformed fragments and it usually attractive (*i.e.*, a negative contribution) for neutral fragments. ΔE_{Pauli} comprises destabilizing interactions between occupied orbitals on each fragment and is responsible for steric repulsion (*i.e.*, a positive contribution). The orbital interaction energy (ΔE_{oi}) accounts for donor-acceptor interactions and polarization effects between and within the fragments, including interactions between the highest occupied and lowest unoccupied MOs (HOMO–LUMO). These interactions cause the electrons to "relax" and stabilize, hence being attractive. This decomposition provides a quantitative framework for understanding the relative contributions of electrostatics, steric effects, orbital interactions, and dispersion forces to tetramer stability.

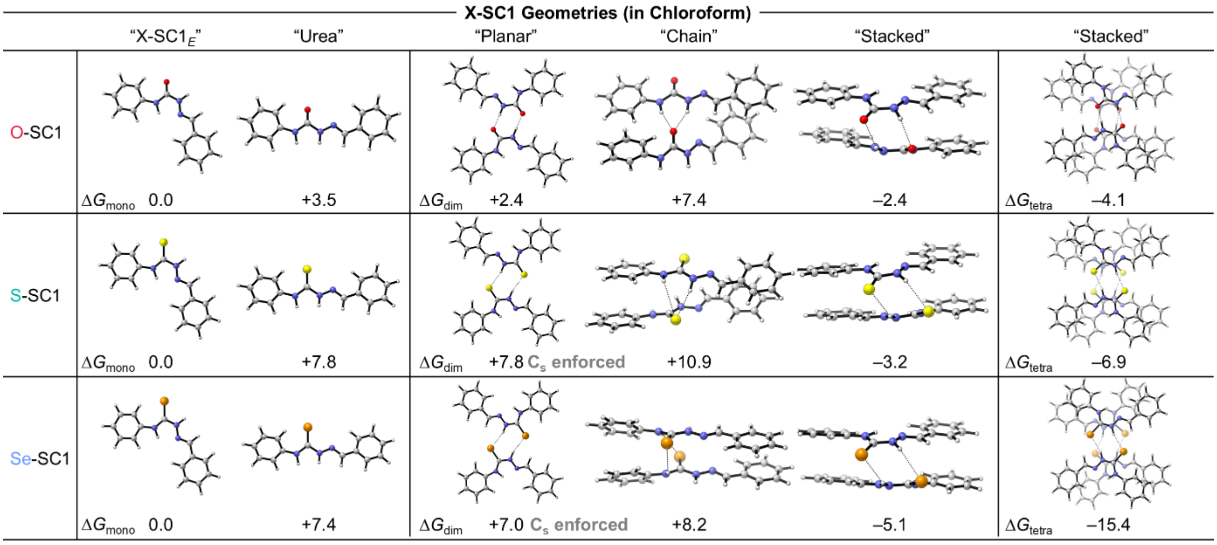

Figure S40. Gibbs free energies of **X-SC1** monomer stability with respect to the global minimum ($\Delta G_{mono} = G_{urea} - G_E$) and Gibbs free energies of the complexation affinity ($\Delta G_{dim} = G_{dimer} - 2^* G_{monomer}$ and $\Delta G_{tetra} = G_{tetramer} - 4^* G_{monomer}$). Energies are reported in kcal mol^{-1}, computed at COSMO(chloroform)-ZORA-BLYP-D3(BJ)/TZ2P.

4.4 Tetramer bonding analysis

To investigate why the formation of a tetramer from four **X-SC1*E*** monomers is increasingly more favorable upon enlarging the chalcogen **X**, we analyzed the components bonding components such as hydrogen bonding and π–π stacking. To achieve this, the activation strand model (ASM, see **Error! Reference source not found.** in the Supporting Information) was employed that decomposes the overall bonding energy associated with forming a tetramer into a deformation term ΔE_{strain} and an interaction term ΔE_{int}. Since we want to gain insight into the type of interactions and their relative importance, we solely focus on ΔE_{int}.

The tetramer was partitioned into three classes of pairwise interactions: (i) two hydrogen-bond interactions (HB^1 and HB^2) between monomers that lie in the same plane (approximately), (ii) two π–π stacking interactions ($[\pi\text{–}\pi]^1$ and $[\pi\text{–}\pi]^2$) between vertically stacked monomers, and (iii) two diagonal interactions between monomers in different stacks ($diag^1$ and $diag^2$). We computed the interaction energy of each pairwise interaction, $\Delta E_{\text{int,HB}}$, $\Delta E_{\text{int,p-p stacking}}$, and $\Delta E_{\text{int,diagonal}}$, and summed over them, yielding $\Delta E_{\text{int,sum}}$. Comparing $\Delta E_{\text{int,sum}}$ in **Table S5** to the interaction energy of four isolated **X-SC1*E*** monomers, ΔE_{int}, we can see that these match very well, justifying our decomposition approach.

The decomposition reveals that hydrogen bonding and p–p stacking are the dominant stabilizing forces, accounting for 54% and 47% of the summed interaction energy in **[O-SC1*E*]4**, respectively. For **[S-SC1*E*]4** and **[Se-SC1*E*]4**, p–p stacking becomes increasingly significant with more negative energies (–44.8 kcal mol^{-1} and–47.8 kcal mol^{-1}), and higher relative contributions (58%, and 59%), while hydrogen bonding contributions decrease slightly (45% and 43%). Also, the hydrogen bonding becomes weaker from **[O-SC1*E*]4** (–40.8 kcal mol^{-1}) to **[S-SC1*E*]4** (34.3 kcal mol^{-1}) and **[Se-SC1*E*]4** (–35.0 kcal mol^{-1}). This shift correlates with enhanced dispersion interactions for heavier chalcogens, as confirmed by energy decomposition analysis.

Table S4. Pairwise decomposition scheme of the **[X-SC1*E*]4** tetramer with interaction energies $\Delta E_{\text{int,[pair]}}$ in kcal mol^{-1}. Also, the relative contributions[(a)] are reported with respect to the summed interaction energy $\Delta E_{\text{int,sum}}$[(b)] in %, computed at ZORA-BLYP-D3(BJ)/TZ2P//COSMO(chloroform)-ZORA-BLYP-D3(BJ)/TZ2P.

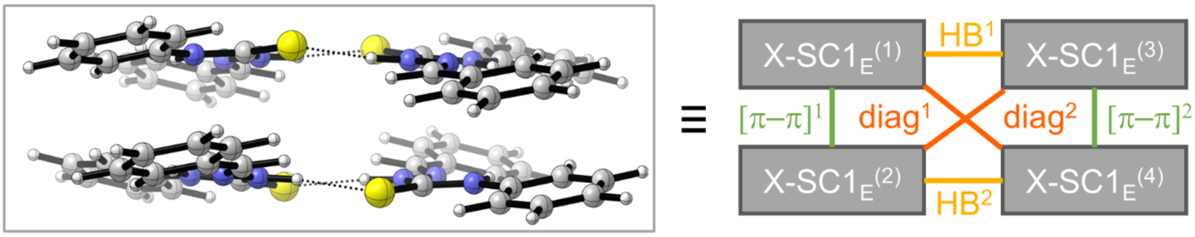

Tetramer	ΔE_{int}[(c)]	$\Delta E_{\text{int,sum}}$[(b)]	$\Sigma\ \Delta E_{\text{int,HB}}$	$\Sigma\ \Delta E_{\text{int,p-p stacking}}$	$\Sigma\ \Delta E_{\text{int,diagonal}}$
**[O-SC1*E*]4**	–75.3	–76.8 (100%)	–40.8 (54%)	–36.3 (47%)	0.4 (–1%)
**[S-SC1*E*]4**	–75.0	–76.8 (100%)	–34.3 (45%)	–44.8 (58%)	2.2 (–3%)
**[Se-SC1*E*]4**	–79.5	–81.4 (100%)	–35.0 (43%)	–47.8 (59%)	1.4 (–2%)

[a] See **Table S6** for all separate terms.

[b] $\Delta E_{\text{int,sum}}$ is the sum of the interaction energies of the two diagonal terms, two hydrogen-bond interactions, and two π–π stacking contributions.

[c] ΔE_{int} is the interaction energy of four, isolated **X-SC1*E*** monomers in the geometry they adopt in the interacting tetramer **[X-SC1*E*]4**.

Table S5. Energy decomposition analyses (EDAs) of the pairwise decomposition of the **X-SC1*E*** tetramer complexes. Energy values are reported in kcal mol^{-1}, computed at ZORA-BLYP-D3(BJ)/TZ2P//COSMO(chloroform)-ZORA-BLYP-D3(BJ)/TZ2P.

**[O-SC1*E*]4**	ΔE_{int}	ΔV_{elstat}	ΔE_{Pauli}	ΔE_{oi}	ΔE_{disp}
Four monomers interacting	–75.3	–80.8	126.0	–48.5	–72.0
Diagonal[1]	0.2	2.5	1.5	–0.9	–2.9
Diagonal[2]	0.2	2.5	1.5	–0.9	–2.9
HB[1]	–20.4	–30.9	36.0	–19.9	–5.6
HB[2]	–20.4	–30.9	36.0	–19.9	–5.6
π–π stacking[1]	–18.1	–12.0	25.7	–4.4	–27.4
π–π stacking[2]	–18.2	–12.0	25.8	–4.4	–27.5
Sum of pairwise interactions[(a)]	–76.8	–80.8	126.4	–50.3	–72.0
**[S-SC1*E*]4**					
Four monomers interacting	–75.0	–70.2	122.5	–43.8	–83.5
Diagonal[1]	1.1	2.9	1.1	–0.7	–2.2
Diagonal[2]	1.1	2.9	1.1	–0.7	–2.2
HB[1]	–17.2	–21.7	28.0	–15.9	–7.5
HB[2]	–17.1	–21.8	28.1	–15.9	–7.5
π–π stacking[1]	–22.4	–16.2	32.7	–6.8	–32.0
π–π stacking[2]	–22.4	–16.2	32.8	–6.8	–32.1
Sum of pairwise interactions[(a)]	–76.8	–70.2	123.7	–46.8	–83.5
**[Se-SC1*E*]4**					
Four monomers interacting	–79.5	–75.1	133.4	–48.7	–89.1
Diagonal[1]	0.7	2.6	1.5	–0.9	–2.5
Diagonal[2]	0.7	2.6	1.6	–0.9	–2.6
HB[1]	–17.5	–22.4	30.5	–17.0	–8.6
HB[2]	–17.5	–22.3	30.4	–17.0	–8.6
π–π stacking[1]	–23.9	–17.8	35.5	–8.2	–33.4
π–π stacking[2]	–23.9	–17.8	35.6	–8.2	–33.4
Sum of pairwise interactions[(a)]	–81.4	–75.1	135.0	–52.1	–89.1

[(a)] The sum includes two diagonal terms, two hydrogen-bond (HB) interactions, and π–π stacking contributions.

4.5 Semicarbazone fragmentation

To understand the role of descending group 16 (X = O, S, and Se) in semicarbazones **X-SC1*E*** on the π_{HOMO}–π_{LUMO} gap, the formation of the C=X bond was analyzed of the semicarbazone. Two fragments were defined: the semicarbazone moiety without the chalcogen, denoted **••SC1*E***, and the chalcogen **••X**. The electron occupations of the fragments were prepared in the triplet state (two unpaired electrons, both either spin up or down; see **Table S7**). The two unpaired electrons of each fragment were distributed over the s and p electronic systems; the former forms the s component of the C=X double bond, and the latter the p component (viewed from a Kohn-Sham MO perspective).

Upon interacting, the fragments experience the potential field of each other (determined by the electrons and nuclei of the other fragment) that changes the shape and energies of the fragment orbitals with respect to isolated fragments. In this study, the fragment orbital energies are reported in the potential field of the full molecule, *i.e.*, the fragments "feel" each other's presence. For a more detailed theoretical background, we refer to the work of Nieuwland *et al.*[39]

Table S7. Occupations of the fragment molecular orbitals (FMOs) belonging to the chalcogen, ••X, and the remaining part, SC1$_E$, of the **X-SC1$_E$** semicarbazone compound with **X** = O, S, and Se. Computed at a ZORA-BLYP-D3(BJ)/TZ2P level of theory with frozen core set to "None".

**X-SC1$_E$**			
Fragment<br># Electrons	Occupations (# spin-a // # spin-b)		SOMOs
**••SC1$_E$**	AA (s)	49 0 // 49 1	49AA (↓)
118 e^-	AAA (p)	9 0 // 9 1	9AA (↓)
C_s symmetry			
**••O**	A.g (s orbitals)	2 // 2	1B1.u (↑)
8 e^-	B1.u (2p$_z$ orbital)	1 // 0	1B3.u (↑)
D_{2h} symmetry	B2.u (2p$_y$ orbital)	1 // 1	
	B3.u (2p$_z$ orbital)	1 // 0	
**••S**	A.g (s orbitals)	3 // 3	2B1.u (↑)
16 e^-	B1.u (*n*p$_z$ orbitals)	2 // 1	2B3.u (↑)
D_{2h} symmetry	B2.u (*n*p$_y$ orbitals)	2 // 2	
	B3.u (*n*p$_z$ orbitals)	2 // 1	
**••Se**	A.g (*n*s + d_{x2-y2} + d_{z2})	6 // 6	3B1.u (↑)
34 e^-	B1.u (*n*p$_z$ orbitals)	3 // 2	3B3.u (↑)
D_{2h} symmetry	B2.u (*n*p$_y$ orbitals)	3 // 3	
	B3.u (*n*p$_z$ orbitals)	3 // 2	
	B1.g (d_{xz} orbital)	1 // 1	
	B2.g (d_{xy} orbital)	1 // 1	
	B3.g ($d_{zy.}$ orbital)	1 // 1	

4.6 ^{1}H NMR trends for X-SC1

^{1}H-NMR spectra recorded in $CDCl_3$ reveal progressive deshielding of the N–H^b proton with increasing concentration for all chalcogen O, S, and Se variants. To probe the origin of this trend, we computed chemical shifts for monomers, stacked dimers, and tetramers, as aggregation is thermodynamically favored. **Figure S42** compares experimental (d^{exp}) and computed (d^{comp}) values. We make two key observations: (i) at low concentrations, the N–H^b proton becomes increasingly deshielded as the chalcogen size grows, with d^{exp} shifting from 7.91 ppm (O) to 9.61 ppm (S) and 9.97 ppm (Se), consistent with computed monomer values (8.40, 9.28, and 9.74 ppm); (ii) aggregation induces pronounced deshielding due to hydrogen bonding between N–H^b and the adjacent C=X group. For instance, d^{comp} for N–H^b in **O-SC1** increases from 8.40 ppm (monomer) to 8.43 ppm (stacked dimer) and 14.19 ppm (tetramer), while **Se-SC1** exhibits an even larger shift from 9.74 ppm to 11.22 ppm and 14.63 ppm. These

findings strongly support the hypothesis that concentration-dependent deshielding arises from aggregation-driven hydrogen bonding.

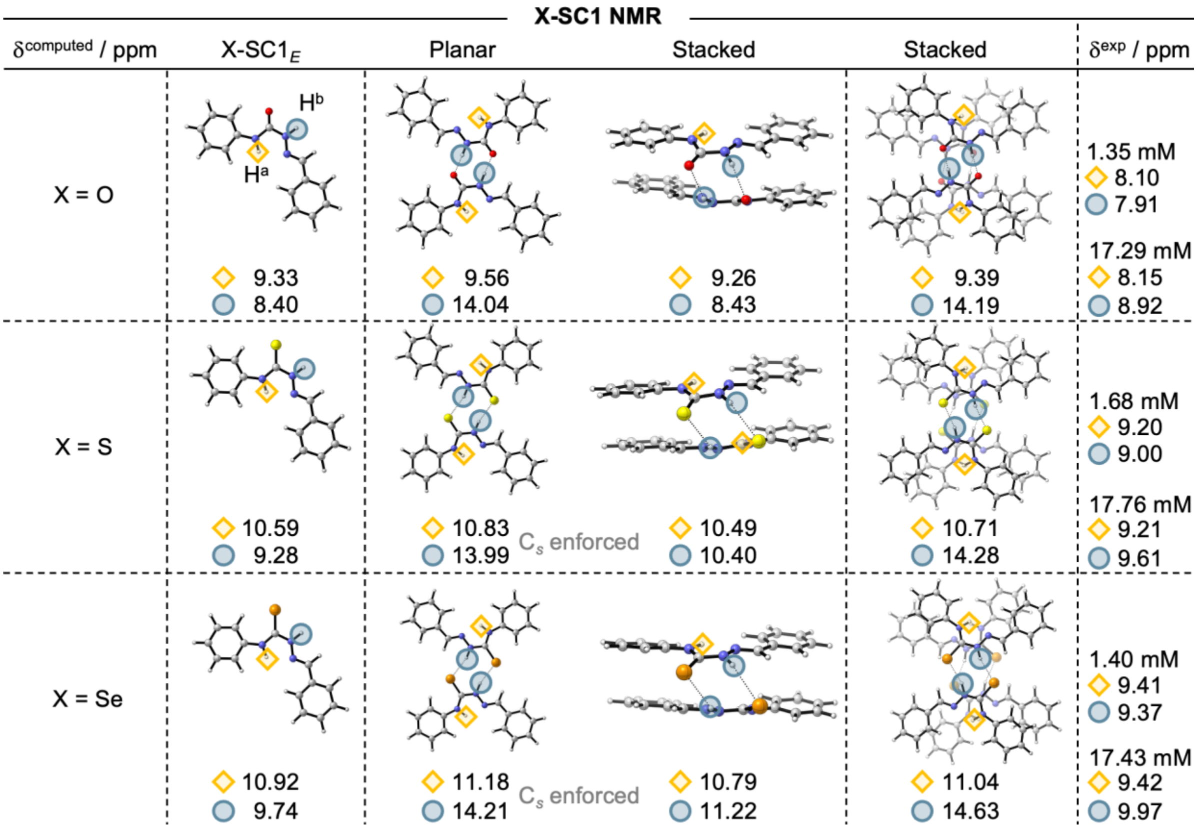

Figure S41. Computed and experimental ^{1}H-NMR chemical shifts (in ppm) of the N-**H**$^{\mathbf{a/b}}$ nuclei, computed at COSMO(Chloroform)-ZORA-SAOP/TZ2P// COSMO(Chloroform)-ZORA-BLYP-D3(BJ)/TZ2P.

Table S8. Calculated ^{1}H-NMR shielding constants s and chemical shifts (δ) of the N–H nuclei (in ppm) of **X-SC1*E*** semicarbazone compounds with **X** = O, S, and Se, computed at COSMO(chloroform)-ZORA-SAOP/TZ2P//COSMO(chloroform)-ZORA-BLYP-D3(BJ)/TZ2P.

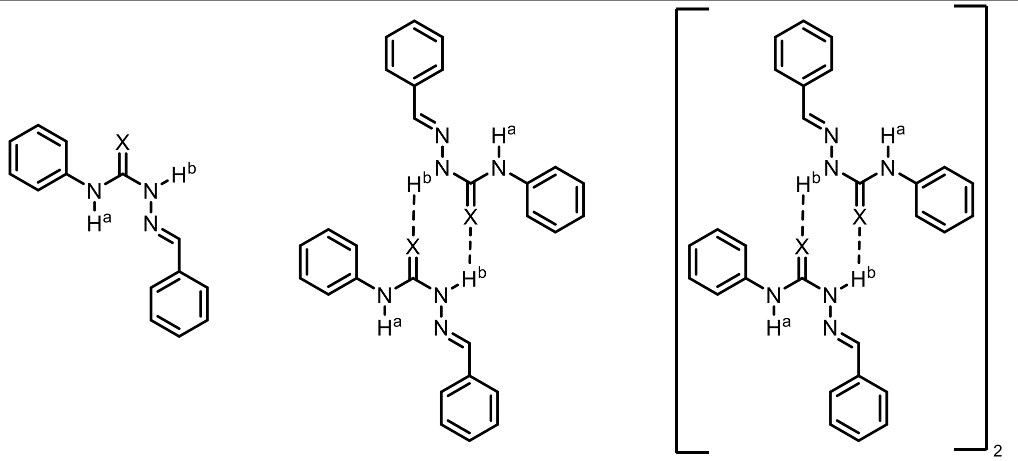

System	σ N-H^a	σ N-H^b	δ N-H^a	δ N-H^b
O-SC1$_E$	22.40	23.33	9.33	8.40
[O-SC1$_E$]$_2$ "Planar" (C$_s$)	22.16	17.69	9.57	14.04
	22.16	17.69	9.27	8.43
[O-SC1$_E$]$_2$ "Stacked"	22.47	23.30	9.26	8.43
	22.46	23.30	9.27	8.43
[O-SC1$_E$]$_4$ "Stacked"	22.34	17.54	9.39	14.19
	22.36	17.53	9.37	14.20
	22.35	17.54	9.38	14.19
	22.35	17.52	9.38	14.21
S-SC1$_E$	21.14	22.45	10.59	9.28
[S-SC1$_E$]$_2$ "Planar" (C$_s$)	20.90	17.74	10.83	13.99
	20.90	17.74	10.83	13.99
[S-SC1$_E$]$_2$ "Stacked"	21.24	21.33	10.49	10.40
	21.25	21.33	10.48	10.40
[S-SC1$_E$]$_4$ "Stacked"	21.02	17.45	10.71	14.28
	21.03	17.44	10.70	14.29
	21.03	17.44	10.70	14.29
	21.01	17.44	10.72	14.29
Se-SC1$_E$	20.81	22.00	10.92	9.73
[Se-SC1$_E$]$_2$ "Planar" (C$_s$)	20.55	17.52	11.18	14.21
	20.55	17.52	11.18	14.21
[Se-SC1$_E$]$_2$ "Stacked"	20.94	20.51	10.79	11.22
	20.94	20.51	10.79	11.22
[Se-SC1$_E$]$_4$ "Stacked"	20.70	17.09	11.03	14.64
	20.70	17.11	11.03	14.62
	20.70	17.09	11.03	14.64
	20.69	17.10	11.04	14.63

Table S9. Experimental ^{1}H-NMR chemical shifts (d) of the N–H nuclei (in ppm) of X-SC1$_E$ semicarbazone compounds with X = O, S, and Se.

**System**	δ N-H^a	δ N-H^b
O-SC1 @ 1.35 mM	8.10	7.91
O-SC1 @ 17.29 mM	8.15	8.92
S-SC1 @ 1.68 mM	9.20	9.00
S-SC1 @ 17.76 mM	9.21	9.61
Se-SC1 @ 1.40 mM	9.41	9.37
Se-SC1 @ 17.43 mM	9.42	9.97

5.1 References

(1) Zakrzewski, J.; Huras, B.; Kiełczewska, A. Synthesis of Isoselenocyanates. *N. Y.* **2016**, *48*, 85–96. https://doi.org/10.1055/s-0035-1560481.

(2) Mahon, M. F.; McGinley, J.; Rooney, A. D.; Walsh, J. M. D. Calix[4]Arene Schiff Bases—Potential Ligands for Fluorescent Studies. *Tetrahedron* **2008**, *64* (49), 11058–11066. https://doi.org/10.1016/j.tet.2008.09.091.

(3) Sentürk, B.; Butschke, B.; Eisenreich, F. Thiosemicarbazones as Versatile Photoswitches with Light-Controllable Supramolecular Activity. *Chem. Sci.* **2025**, *16* (7), 3130–3140. https://doi.org/10.1039/D4SC08530B.

(4) Mabesoone, M. F. J.; Ter Huurne, G. M.; Palmans, A. R. A.; Meijer, E. W. How Water in Aliphatic Solvents Directs the Interference of Chemical Reactivity in a Supramolecular System. *J. Am. Chem. Soc.* **2020**, *142* (28), 12400–12408. https://doi.org/10.1021/jacs.0c04962.

(5) Van Zee, N. J.; Adelizzi, B.; Mabesoone, M. F. J.; Meng, X.; Aloi, A.; Zha, R. H.; Lutz, M.; Filot, I. A. W.; Palmans, A. R. A.; Meijer, E. W. Potential Enthalpic Energy of Water in Oils Exploited to Control Supramolecular Structure. *Nature* **2018**, *558* (7708), 100–103. https://doi.org/10.1038/s41586-018-0169-0.

(6) T. Zick, S. Engel (BASF SE), CN 102076635 A, 2011.

(7) Casimiro, L.; Andreoni, L.; Groppi, J.; Credi, A.; Métivier, R.; Silvi, S. 4,4′-Dimethylazobenzene as a Chemical Actinometer. *Photochem. Photobiol. Sci.* **2022**, *21* (5), 825–833. https://doi.org/10.1007/s43630-021-00162-3.

(8) Stranius, K.; Börjesson, K. Determining the Photoisomerization Quantum Yield of Photoswitchable Molecules in Solution and in the Solid State. *Sci. Rep.* **2017**, *7* (1), 41145. https://doi.org/10.1038/srep41145.

(9) Larsen, D.; Langhorn, L. M.; Akselsen, O. M.; Nielsen, B. E.; Pittelkow, M. Thiosemicarbazone Organocatalysis: Tetrahydropyranylation and 2-Deoxygalactosylation Reactions and Kinetics-Based Mechanistic Investigation. *Chem. Sci.* **2017**, *8* (12), 7978–7982. https://doi.org/10.1039/C7SC03366D.

(10) Baerends, E. J.; Aguirre, N. F.; Austin, N. D.; Autschbach, J.; Bickelhaupt, F. M.; Bulo, R.; Cappelli, C.; Van Duin, A. C. T.; Egidi, F.; Fonseca Guerra, C.; Förster, A.; Franchini, M.; Goumans, T. P. M.; Heine, T.; Hellström, M.; Jacob, C. R.; Jensen, L.; Krykunov, M.; Van Lenthe, E.; Michalak, A.; Mitoraj, M. M.; Neugebauer, J.; Nicu, V. P.; Philipsen, P.; Ramanantoanina, H.; Rüger, R.; Schreckenbach, G.; Stener, M.; Swart, M.; Thijssen, J. M.; Trnka, T.; Visscher, L.; Yakovlev, A.; Van Gisbergen, S. The Amsterdam Modeling Suite. *J. Chem. Phys.* **2025**, *162* (16), 162501. https://doi.org/10.1063/5.0258496.

(11) Te Velde, G.; Bickelhaupt, F. M.; Baerends, E. J.; Fonseca Guerra, C.; Van Gisbergen, S. J. A.; Snijders, J. G.; Ziegler, T. Chemistry with ADF. *J. Comput. Chem.* **2001**, *22* (9), 931–967. https://doi.org/10.1002/jcc.1056.

(12) Becke, A. D. Density-Functional Exchange-Energy Approximation with Correct Asymptotic Behavior. *Phys. Rev. A* **1988**, *38* (6), 3098–3100. https://doi.org/10.1103/PhysRevA.38.3098.

(13) Lee, C.; Yang, W.; Parr, R. G. Development of the Colle-Salvetti Correlation-Energy Formula into a Functional of the Electron Density. *Phys. Rev. B* **1988**, *37* (2), 785–789. https://doi.org/10.1103/PhysRevB.37.785.

(14) Grimme, S.; Antony, J.; Ehrlich, S.; Krieg, H. A Consistent and Accurate *Ab Initio* Parametrization of Density Functional Dispersion Correction (DFT-D) for the 94 Elements H-Pu. *J. Chem. Phys.* **2010**, *132* (15), 154104. https://doi.org/10.1063/1.3382344.

(15) Grimme, S.; Ehrlich, S.; Goerigk, L. Effect of the Damping Function in Dispersion Corrected Density Functional Theory. *J. Comput. Chem.* **2011**, *32* (7), 1456–1465. https://doi.org/10.1002/jcc.21759.

(16) Van Lenthe, E.; Baerends, E. J.; Snijders, J. G. Relativistic Total Energy Using Regular Approximations. *J. Chem. Phys.* **1994**, *101* (11), 9783–9792. https://doi.org/10.1063/1.467943.

(17) Nieuwland, C.; Zaccaria, F.; Fonseca Guerra, C. Understanding Alkali Metal Cation Affinities of Multi-Layer Guanine Quadruplex DNA. *Phys. Chem. Chem. Phys.* **2020**, *22* (37), 21108–21118. https://doi.org/10.1039/D0CP03433A.

(18) Vermeeren, P.; Wolters, L. P.; Paragi, G.; Fonseca Guerra, C. Cooperative Self-Assembly in Linear Chains Based on Halogen Bonds. *ChemPlusChem* **2021**, *86* (6), 812–819. https://doi.org/10.1002/cplu.202100093.

(19) Mitchell, E. C.; Azevedo Santos, L.; Vermeeren, P.; Lahm, M. E.; Fonseca Guerra, C.; Schaefer, H. F.; Bickelhaupt, F. M. Hydrogen Bond Benchmark: Focal-Point Analysis and Assessment of DFT Functionals. *J. Comput. Chem.* **2025**, *46* (30), e70265. https://doi.org/10.1002/jcc.70265.

(20) Van Lenthe, E.; Baerends, E. J. Optimized Slater-type Basis Sets for the Elements 1–118. *J. Comput. Chem.* **2003**, *24* (9), 1142–1156. https://doi.org/10.1002/jcc.10255.

(21) Franchini, M.; Philipsen, P. H. T.; Van Lenthe, E.; Visscher, L. Accurate Coulomb Potentials for Periodic and Molecular Systems through Density Fitting. *J. Chem. Theory Comput.* **2014**, *10* (5), 1994–2004. https://doi.org/10.1021/ct500172n.

(22) Fonseca Guerra, C.; Van Der Wijst, T.; Poater, J.; Swart, M.; Bickelhaupt, F. M. Adenine versus Guanine Quartets in Aqueous Solution: Dispersion-Corrected DFT Study on the Differences in π-Stacking and Hydrogen-Bonding Behavior. *Theor. Chem. Acc.* **2010**, *125* (3–6), 245–252. https://doi.org/10.1007/s00214-009-0634-9.

(23) Becke, A. D. A Multicenter Numerical Integration Scheme for Polyatomic Molecules. *J. Chem. Phys.* **1988**, *88* (4), 2547–2553. https://doi.org/10.1063/1.454033.

(24) Klamt, A.; Schüürmann, G. COSMO: A New Approach to Dielectric Screening in Solvents with Explicit Expressions for the Screening Energy and Its Gradient. *J Chem Soc Perkin Trans 2* **1993**, No. 5, 799–805. https://doi.org/10.1039/P29930000799.

(25) Legault; Claude. CYLview2.0, 2020.

(26) Pracht, P.; Bohle, F.; Grimme, S. Automated Exploration of the Low-Energy Chemical Space with Fast Quantum Chemical Methods. *Phys. Chem. Chem. Phys.* **2020**, *22* (14), 7169–7192. https://doi.org/10.1039/C9CP06869D.

(27) Wolff, S. K. Analytical Second Derivatives in the Amsterdam Density Functional Package. *Int. J. Quantum Chem.* **2005**, *104* (5), 645–659. https://doi.org/10.1002/qua.20653.

(28) Bérces, A.; Dickson, R. M.; Fan, L.; Jacobsen, H.; Swerhone, D.; Ziegler, T. An Implementation of the Coupled Perturbed Kohn-Sham Equations: Perturbation Due to Nuclear Displacements. *Comput. Phys. Commun.* **1997**, *100* (3), 247–262. https://doi.org/10.1016/S0010-4655(96)00120-8.

(29) Rosa, A.; Baerends, E. J.; Van Gisbergen, S. J. A.; Van Lenthe, E.; Groeneveld, J. A.; Snijders, J. G. Electronic Spectra of $M(CO)_6$ (M = Cr, Mo, W) Revisited by a Relativistic TDDFT Approach. *J. Am. Chem. Soc.* **1999**, *121* (44), 10356–10365. https://doi.org/10.1021/ja990747t.

(30) Van Gisbergen, S. J. A.; Snijders, J. G.; Baerends, E. J. Implementation of Time-Dependent Density Functional Response Equations. *Comput. Phys. Commun.* **1999**, *118* (2–3), 119–138. https://doi.org/10.1016/S0010-4655(99)00187-3.

(31) Seth, M.; Ziegler, T. Range-Separated Exchange Functionals with Slater-Type Functions. *J. Chem. Theory Comput.* **2012**, *8* (3), 901–907. https://doi.org/10.1021/ct300006h.

(32) Dev, P.; Agrawal, S.; English, N. J. Determining the Appropriate Exchange-Correlation Functional for Time-Dependent Density Functional Theory Studies of Charge-Transfer Excitations in Organic Dyes. *J. Chem. Phys.* **2012**, *136* (22), 224301. https://doi.org/10.1063/1.4725540.

(33) Bickelhaupt, F. M.; Houk, K. N. Analyzing Reaction Rates with the Distortion/Interaction-Activation Strain Model. *Angew. Chem. Int. Ed.* **2017**, *56* (34), 10070–10086. https://doi.org/10.1002/anie.201701486.

(34) Vermeeren, P.; Van Der Lubbe, S. C. C.; Fonseca Guerra, C.; Bickelhaupt, F. M.; Hamlin, T. A. Understanding Chemical Reactivity Using the Activation Strain Model. *Nat. Protoc.* **2020**, *15* (2), 649–667. https://doi.org/10.1038/s41596-019-0265-0.

(35) Van Zeist, W.-J.; Bickelhaupt, F. M. The Activation Strain Model of Chemical Reactivity. *Org. Biomol. Chem.* **2010**, *8* (14), 3118. https://doi.org/10.1039/b926828f.

(36) Vermeeren, P.; Hamlin, T. A.; Bickelhaupt, F. M. Chemical Reactivity from an Activation Strain Perspective. *Chem. Commun.* **2021**, *57* (48), 5880–5896. https://doi.org/10.1039/D1CC02042K.

(37) Bickelhaupt, M. F.; Baerends, E. J. Reviews in Computational Chemistry. In *Kohn-Sham Density Functional Theory: Predicting and Understanding Chemistry*; John Wiley&Sons Inc., 2007; pp 1–86. https://doi.org/10.1002/9780470125922.

(38) Hamlin, T. A.; Vermeeren, P.; Célia Fonseca Guerra; F. Matthias Bickelhaupt. Energy Decomposition Analysis in the Context of Quantitative Molecular Orbital Theory. In *Complementary Bonding Analysis*; G. Simon, De Gruyter: Berlin, Boston, 2021; pp 199–212. https://doi.org/10.1515/9783110660074-008.

(39) Nieuwland, C.; Fonseca Guerra, C. How the Chalcogen Atom Size Dictates the Hydrogen-Bond Donor Capability of Carboxamides, Thioamides, and Selenoamides. *Chem. – Eur. J.* **2022**, *28* (31), e202200755. https://doi.org/10.1002/chem.202200755.

Cartesian Coordinates

Table S10. Cartesian coordinates (in Å), absolute energies, enthalpies, and Gibbs free energies (in kcal mol^{-1}) for the semicarbazone compounds **X-SC1**, computed at ZORA-BLYP-D3(BJ)/TZ2P in gas phase.

O-SC1$_{E}$

E = –4528.15 kcal mol^{-1}
H = –4367.77 kcal mol^{-1}
G = –4406.79 kcal mol^{-1}
N_{imag} = 0

C	-4.99991366	-1.41901676	0.00682866
C	-1.46281773	3.45647967	0.01100928
H	-1.98005413	5.54215012	0.01141877
C	-6.05287282	-4.01458369	0.00691091
H	1.70719167	2.19981758	0.01105561
C	0.91136325	2.93346774	0.01109306
H	2.22841505	4.62716785	0.01196455
N	-2.08799823	-1.21710315	0.00742307
H	-6.45848791	-5.02373047	0.00697260
N	-0.80505851	1.14344939	0.01007832
H	-2.49983357	3.12351732	0.01065986
C	0.16348052	5.25323623	0.01162240
C	-6.91292977	-2.91204768	0.00670557
C	-1.16737241	4.81917511	0.01142778
H	-1.80143993	0.93777261	0.00896511
H	-0.16272285	-2.02647216	0.00872698
C	-2.66899033	-2.37234082	0.00729396
H	-7.99041500	-3.05862378	0.00655544
C	1.18978197	4.30327631	0.01166392
C	0.00029401	0.02935325	0.00959866
H	-2.07449898	-3.29771151	0.00729631
C	-0.42720864	2.50248170	0.01078789
C	-6.37836325	-1.61478053	0.00665084
N	-0.73045642	-1.17605971	0.00739665
C	-4.12166101	-2.52403218	0.00707625
H	0.39513008	6.31541577	0.01192820
O	1.22941772	-0.00756417	0.01075667
H	-4.00262840	-4.68296247	0.00729069
C	-4.66961624	-3.82251822	0.00710200
H	-4.58665222	-0.41459660	0.00675488
H	-7.04449119	-0.75472507	0.00644351

O-SC1$_{E}$ (planar, C_s symmetry)

E = –4528.15 kcal mol^{-1}
H = –4367.77 kcal mol^{-1}

G = –4406.79 kcal mol^{-1}
N_{imag} = 0

C	-4.99986836	-1.41889894	0.00000000
C	-1.46283592	3.45629778	0.00000000
H	-1.98027005	5.54190752	0.00000000
C	-6.05297538	-4.01437420	0.00000000
H	1.70715164	2.19979358	0.00000000
C	0.91138826	2.93350805	0.00000000
H	2.22830073	4.62725920	0.00000000
N	-2.08786029	-1.21717823	0.00000000
H	-6.45864736	-5.02349935	0.00000000
N	-0.80490309	1.14334174	0.00000000
H	-2.49982000	3.12323270	0.00000000
C	0.16330336	5.25318289	0.00000000
C	-6.91295374	-2.91180249	0.00000000
C	-1.16752129	4.81899953	0.00000000
H	-1.80122124	0.93748177	0.00000000
H	-0.16233576	-2.02641394	0.00000000
C	-2.66895090	-2.37236431	0.00000000
H	-7.99045222	-3.05829361	0.00000000
C	1.18965747	4.30335444	0.00000000
C	0.00045354	0.02921058	0.00000000
H	-2.07462183	-3.29786932	0.00000000
C	-0.42713553	2.50237940	0.00000000
C	-6.37832439	-1.61457281	0.00000000
N	-0.73030741	-1.17616890	0.00000000
C	-4.12167357	-2.52395462	0.00000000
H	0.39482902	6.31538606	0.00000000
O	1.22960669	-0.00779167	0.00000000
H	-4.00276442	-4.68286026	0.00000000
C	-4.66971259	-3.82238833	0.00000000
H	-4.58651426	-0.41451624	0.00000000
H	-7.04443006	-0.75449629	0.00000000

O-SC1*E*
E = –4450.35 kcal mol^{-1}
H = –4291.08 kcal mol^{-1}
G = –4330.83 kcal mol^{-1}
N_{imag} = 0

C	-4.90673239	-1.60428604	0.00729084
C	-1.75537921	3.25374619	0.01114869
H	-2.56325947	5.24401005	0.00896047
C	-5.87163588	-4.23428766	0.00814403
H	1.56072374	2.45802995	0.01560247
C	0.66727248	3.06852813	0.01413716
H	1.73235771	4.93021923	0.01424951
N	-2.00411061	-1.30349480	0.01303485

H	-6.24344493	-5.25632729	0.00847907
N	-0.81018111	1.05721920	0.01355010
H	-2.73521247	2.77802726	0.01031423
C	-0.39952532	5.26017867	0.01154477
C	-6.76799117	-3.16086930	0.00523492
C	-1.65666939	4.64324643	0.01038961
H	-1.79135781	0.77353773	0.01169321
H	-0.05597730	-2.05840155	0.01818309
C	-2.54736570	-2.47985518	0.01307493
H	-7.83994274	-3.34344093	0.00331166
C	0.74936904	4.46429967	0.01337439
C	0.03516779	-0.00985239	0.01651103
H	-1.92109440	-3.38343207	0.01529780
C	-0.59528459	2.45254590	0.01305045
C	-6.27749025	-1.84618497	0.00481091
N	-0.64933840	-1.22537074	0.01585522
C	-3.99175185	-2.67970749	0.01027452
H	-0.31956795	6.34438663	0.01098749
S	1.71144975	-0.06278359	0.02075583
H	-3.80036182	-4.83383196	0.01290187
C	-4.49593084	-3.99642113	0.01062700
H	-4.52752350	-0.58655581	0.00697805
H	-6.97225601	-1.00918275	0.00253461

S-SC1*E* (planar, *C*s symmetry)

E = –4450.37 kcal mol^{-1}

H = –4291.09 kcal mol^{-1}

G = –4330.83 kcal mol^{-1}

N_{imag} = 0

C	-4.93481980	-1.34060535	0.00000000
C	-1.48178213	3.30846664	0.00000000
H	-2.16238237	5.34578363	0.00000000
C	-6.06209902	-3.90528241	0.00000000
H	1.77756144	2.30521495	0.00000000
C	0.92442553	2.97089680	0.00000000
H	2.10490162	4.76172233	0.00000000
N	-2.01784770	-1.22324787	0.00000000
H	-6.49700536	-4.90210681	0.00000000
N	-0.67695004	1.05676911	0.00000000
H	-2.48966520	2.89543965	0.00000000
C	-0.00199628	5.22545575	0.00000000
C	-6.88968482	-2.77798040	0.00000000
C	-1.29552118	4.68898944	0.00000000
H	-1.67407408	0.83561105	0.00000000
H	-0.12187076	-2.10051537	0.00000000
C	-2.63465364	-2.36275272	0.00000000
H	-7.97092835	-2.89323947	0.00000000
C	1.09448752	4.35870304	0.00000000

C	0.09933971	-0.06153326	0.00000000
H	-2.06688353	-3.30419891	0.00000000
C	-0.37447619	2.43570259	0.00000000
C	-6.31805791	-1.49646721	0.00000000
N	-0.66083778	-1.23131723	0.00000000
C	-4.08877791	-2.47112671	0.00000000
H	0.14616756	6.30246660	0.00000000
S	1.76887892	-0.22001624	0.00000000
H	-4.03221569	-4.63298250	0.00000000
C	-4.67422632	-3.75380067	0.00000000
H	-4.49295305	-0.34847114	0.00000000
H	-6.95920402	-0.61768562	0.00000000

Se-SC1*E*

E = –4429.48 kcal mol^{-1}

H = –4271.09 kcal mol^{-1}

G = –4309.71 kcal mol^{-1}

N_{imag} = 0

C	-4.41082988	-4.08904798	0.00985019
C	-1.83973957	3.22203238	0.01156865
H	-2.69596428	5.19208986	0.00996532
C	-6.24841411	-1.98608150	0.00404866
H	1.49480970	2.50663734	0.01270681
C	0.58672184	3.09640560	0.01208280
H	1.60644536	4.98339608	0.01086506
N	-1.99020120	-1.33155142	0.01227397
H	-6.96507465	-1.16774723	0.00173005
N	-0.84131629	1.04968097	0.01315680
H	-2.80744758	2.72197544	0.01172039
C	-0.53317008	5.26071007	0.01025069
C	-6.70397514	-3.31330893	0.00461269
C	-1.77509212	4.61354091	0.01058748
H	-1.81849266	0.74875511	0.01154567
H	-0.02476177	-2.04542110	0.01725238
C	-2.50356802	-2.52208665	0.01203660
H	-7.77072846	-3.52410119	0.00279252
C	0.63517851	4.49356144	0.01106660
C	0.01474867	-0.00053019	0.01590290
H	-1.85416980	-3.40906965	0.01402191
C	-0.65995701	2.45074966	0.01241003
C	-5.77967521	-4.36288909	0.00747920
N	-0.63549363	-1.22446436	0.01495180
C	-3.94154008	-2.75925967	0.00938591
H	-0.47983380	6.34657510	0.00943930
Se	1.85970861	-0.05589995	0.02085875
H	-4.53234634	-0.68074533	0.00588997
C	-4.88457153	-1.70812333	0.00639414

H	-3.69330328	-4.90772031	0.01208258
H	-6.12454761	-5.39432180	0.00791817

Se-SC1$_E$ (planar, C_s symmetry)

E = –4429.49 kcal mol^{-1}

H = –4270.51 kcal mol^{-1}

G = –4311.10 kcal mol^{-1}

N_{imag} = 0

C	-4.94023281	-1.34019463	0.00000000
C	-1.48010938	3.30980508	0.00000000
H	-2.16303225	5.34647542	0.00000000
C	-6.05905328	-3.90886454	0.00000000
H	1.78030658	2.30930874	0.00000000
C	0.92649901	2.97526137	0.00000000
H	2.10528554	4.76724103	0.00000000
N	-2.02347401	-1.21351733	0.00000000
H	-6.49081536	-4.90703604	0.00000000
N	-0.67275428	1.05934803	0.00000000
H	-2.48737643	2.89519940	0.00000000
C	-0.00244782	5.22816159	0.00000000
C	-6.89023426	-2.78409549	0.00000000
C	-1.29555388	4.69057982	0.00000000
H	-1.67230876	0.84401853	0.00000000
H	-0.12736312	-2.09482277	0.00000000
C	-2.63750825	-2.35538696	0.00000000
H	-7.97108751	-2.90286184	0.00000000
C	1.09538002	4.36304467	0.00000000
C	0.08921604	-0.06099554	0.00000000
H	-2.06667752	-3.29484774	0.00000000
C	-0.37122653	2.43958964	0.00000000
C	-6.32286481	-1.50064348	0.00000000
N	-0.66467068	-1.22403756	0.00000000
C	-4.09051131	-2.46819488	0.00000000
H	0.14434727	6.30537945	0.00000000
Se	1.92234184	-0.27631392	0.00000000
H	-4.02687821	-4.63000199	0.00000000
C	-4.67182134	-3.75299896	0.00000000
H	-4.50155042	-0.34665904	0.00000000
H	-6.96697491	-0.62404838	0.00000000

O-SC1Z

E = –4525.38 kcal mol–1

H = –4364.67 kcal mol–1

G = –4403.23 kcal mol–1

Nimag = 0

C	0.05088425	0.07104389	-0.07528114

O	1.26453477	0.08717223	-0.27118626
N	-0.78165074	1.14435138	0.12113177
N	-0.63316003	-1.16860082	-0.06596303
C	-0.46050999	2.51745119	0.13573820
H	-1.75079393	0.88691864	0.29939620
N	-1.94007145	-1.24865922	0.31160327
H	-0.01517501	-1.98082893	-0.04806961
C	0.83748317	3.01049503	-0.08759816
C	-1.51187192	3.42025757	0.38534747
C	-2.52420575	-2.39657652	0.46103178
C	1.06076236	4.39008983	-0.05858842
H	1.64555802	2.31572354	-0.27743854
C	-1.27157462	4.79350306	0.41097793
H	-2.51719788	3.03885684	0.55831181
C	-2.01680605	-3.76607057	0.26833415
H	-3.55045921	-2.31341884	0.82105980
C	0.01856937	5.28926436	0.18861993
H	2.06823923	4.76220478	-0.23247651
H	-2.09556025	5.47657225	0.60515080
C	-1.04876162	-4.12344775	-0.69620818
C	-2.57130494	-4.78939127	1.06714075
H	0.20688948	6.35983913	0.20836775
C	-0.63657473	-5.45114322	-0.83101674
H	-0.65328302	-3.37848995	-1.38014129
C	-2.14875928	-6.11167866	0.93766621
H	-3.33471502	-4.53326320	1.79924879
C	-1.17401169	-6.44764280	-0.00964822
H	0.10274117	-5.70776183	-1.58624670
H	-2.58205931	-6.88212767	1.57144641
H	-0.84465218	-7.47864868	-0.11397756

O-SC1Z (planar, Cs symmetry)
E = –4524.13 kcal mol–1
H = –4364.08 kcal mol–1
G = –4401.36 kcal mol–1
Nimag = 1 (64.8i cm–1)

C	-0.01663461	0.00923224	0.00000000
O	1.21272767	-0.01792525	0.00000000
N	-0.83417162	1.11036359	0.00000000
N	-0.73358009	-1.21119625	0.00000000
C	-0.47485727	2.47413321	0.00000000
H	-1.82757513	0.88452648	0.00000000

N	-2.08708921	-1.24246373	0.00000000
H	-0.14153656	-2.03652543	0.00000000
C	0.85818329	2.92203914	0.00000000
C	-1.52280772	3.41447448	0.00000000
C	-2.77451965	-2.34434306	0.00000000
C	1.11928955	4.29524836	0.00000000
H	1.66315568	2.19832785	0.00000000
C	-1.24453152	4.78075980	0.00000000
H	-2.55519766	3.06779030	0.00000000
C	-2.42937918	-3.77182042	0.00000000
H	-3.84819047	-2.15130627	0.00000000
C	0.08072932	5.23176582	0.00000000
H	2.15369762	4.63246441	0.00000000
H	-2.06626655	5.49345524	0.00000000
C	-1.13493693	-4.34434324	0.00000000
C	-3.53046138	-4.66354716	0.00000000
H	0.29869435	6.29687195	0.00000000
C	-0.96236985	-5.73028699	0.00000000
H	-0.23575817	-3.74084929	0.00000000
C	-3.35618550	-6.04457372	0.00000000
H	-4.53675125	-4.24969330	0.00000000
C	-2.06604635	-6.58790370	0.00000000
H	0.04507317	-6.13970269	0.00000000
H	-4.22530166	-6.69845398	0.00000000
H	-1.92262520	-7.66564160	0.00000000

O-SC1*Z*

E = –4447.58 kcal mol^{-1}

H = –4288.00 kcal mol^{-1}

G = –4327.44 kcal mol^{-1}

N_{imag} = 0

C	-0.05173328	-0.11367554	-0.24366511
S	1.55864897	-0.32402462	-0.66142670
N	-0.75818783	1.02388621	-0.01094927
N	-0.83075652	-1.26525244	-0.08580317
C	-0.40623202	2.39074799	-0.01824634
H	-1.74267406	0.82960731	0.18658723
N	-2.17235795	-1.19125281	0.12537839
H	-0.36515646	-2.13102900	-0.35915652
C	0.89185403	2.88107504	-0.23809689
C	-1.45709865	3.29913538	0.22280316
C	-2.89165323	-2.27052211	0.20116228
C	1.11700633	4.26079570	-0.21546825

H	1.70137639	2.18756614	-0.42465411
C	-1.21627675	4.67106823	0.24211479
H	-2.46385127	2.92043558	0.39380497
C	-2.50436136	-3.68828584	0.12992611
H	-3.95870195	-2.06937233	0.30211144
C	0.07626240	5.16300885	0.02198526
H	2.12610759	4.62908863	-0.38727788
H	-2.04003072	5.35615876	0.42905892
C	-1.26929944	-4.19717586	0.59075588
C	-3.44948012	-4.60023296	-0.38873919
H	0.26660025	6.23333491	0.03605879
C	-0.98763277	-5.56217857	0.50566389
H	-0.54491649	-3.54163485	1.06482205
C	-3.15935813	-5.96046373	-0.48302807
H	-4.41386458	-4.22663131	-0.72740536
C	-1.92315567	-6.44699527	-0.04083695
H	-0.03492561	-5.93532782	0.87407687
H	-3.89790138	-6.64307372	-0.89721140
H	-1.69563811	-7.50790290	-0.11147403

S-SC1$_Z$ (planar, C_s symmetry)

E = –4446.46 kcal mol^{-1}

H = –4287.52 kcal mol^{-1}

G = –4325.49 kcal mol^{-1}

N_{imag} = 1 (56.8*i* cm^{-1})

C	0.10538702	-0.04861522	0.00000000
S	1.77524143	-0.20052701	0.00000000
N	-0.67902030	1.05979842	0.00000000
N	-0.64535324	-1.23133185	0.00000000
C	-0.38919776	2.44103505	0.00000000
H	-1.67427676	0.82205274	0.00000000
N	-1.99805049	-1.21228781	0.00000000
H	-0.08376549	-2.07773726	0.00000000
C	0.90573265	2.98599961	0.00000000
C	-1.50355040	3.30464040	0.00000000
C	-2.71898074	-2.29529673	0.00000000
C	1.06523165	4.37500849	0.00000000
H	1.76406493	2.32692210	0.00000000
C	-1.32764617	4.68652554	0.00000000
H	-2.50782063	2.88326185	0.00000000
C	-2.41485591	-3.73045178	0.00000000
H	-3.78620087	-2.07020554	0.00000000

C	-0.03821503	5.23293446	0.00000000
H	2.07242577	4.78603412	0.00000000
H	-2.19945277	5.33675287	0.00000000
C	-1.13731937	-4.34114650	0.00000000
C	-3.54240969	-4.58919327	0.00000000
H	0.10154674	6.31109199	0.00000000
C	-1.00693628	-5.73133592	0.00000000
H	-0.21889515	-3.76708641	0.00000000
C	-3.40938551	-5.97460597	0.00000000
H	-4.53593700	-4.14560315	0.00000000
C	-2.13578804	-6.55575920	0.00000000
H	-0.01210269	-6.17038003	0.00000000
H	-4.29750503	-6.60235728	0.00000000
H	-2.02435458	-7.63725993	0.00000000

Se-SC1*Z*
E = –4426.73 kcal mol–1
H = –4267.45 kcal mol–1
G = –4307.79 kcal mol–1
Nimag = 0

C	-0.73533441	-4.13772416	-0.78587877
C	-1.88577040	3.19614743	0.24283543
H	-2.80644945	5.12922005	0.41557028
C	-1.83874045	-6.23160669	0.70888595
H	1.42738371	2.61940065	-0.32609405
C	0.50949736	3.17126990	-0.16617738
H	1.43958776	5.09885720	-0.30930441
N	-1.84700715	-1.34495135	0.23800097
H	-2.27279579	-7.04332912	1.28820799
N	-0.81497117	1.06753400	0.04292223
H	-2.81884536	2.65617291	0.39794866
C	-0.67889990	5.28711133	0.05391034
C	-0.78303926	-6.48569985	-0.17515688
C	-1.87667533	4.58915120	0.25251454
H	-1.76490220	0.72053919	0.20273822
H	0.12901954	-1.98273987	-0.05367014
C	-2.37576905	-2.52962158	0.32073109
H	-0.39160418	-7.49421471	-0.28449112
C	0.50225809	4.56920426	-0.15370099
C	0.07195997	0.05594337	-0.09917290
H	-3.42320817	-2.51087952	0.62373058
C	-0.69302397	2.47471902	0.03397336
C	-0.24422278	-5.43698928	-0.92789035

N	-0.52347518	-1.19827233	-0.04534785
C	-1.78565864	-3.86150697	0.11860536
H	-0.66921491	6.37426186	0.06139302
Se	1.89857347	0.07289199	-0.36231277
H	-3.16397480	-4.74527475	1.52957625
C	-2.33973210	-4.93811431	0.84567904
H	-0.33121989	-3.35464560	-1.42037965
H	0.55923428	-5.62995118	-1.63484289

Se-SC1$_Z$ (planar, C_s symmetry)
E = –4425.67 kcal mol^{-1}
H = –4267.02 kcal mol^{-1}
G = –4305.80 kcal mol^{-1}
N_{imag} = 1 (49.9*i* cm^{-1})

C	0.09118845	-0.05161475	0.00000000
Se	1.92432735	-0.26144600	0.00000000
N	-0.67870525	1.05940009	0.00000000
N	-0.65327387	-1.22704669	0.00000000
C	-0.38870661	2.44169745	0.00000000
H	-1.67660846	0.82796545	0.00000000
N	-2.00801411	-1.20622348	0.00000000
H	-0.09311606	-2.07486551	0.00000000
C	0.90559174	2.98590012	0.00000000
C	-1.50384280	3.30374253	0.00000000
C	-2.72478753	-2.29269653	0.00000000
C	1.06517234	4.37473496	0.00000000
H	1.76421347	2.32601735	0.00000000
C	-1.32835881	4.68571258	0.00000000
H	-2.50786886	2.88171346	0.00000000
C	-2.41586938	-3.72636253	0.00000000
H	-3.79280234	-2.07121607	0.00000000
C	-0.03884185	5.23201876	0.00000000
H	2.07220669	4.78603858	0.00000000
H	-2.20015312	5.33586379	0.00000000
C	-1.13636495	-4.33343879	0.00000000
C	-3.54121108	-4.58826032	0.00000000
H	0.10054816	6.31024383	0.00000000
C	-1.00242359	-5.72312098	0.00000000
H	-0.21874912	-3.75793872	0.00000000
C	-3.40435060	-5.97323917	0.00000000
H	-4.53595187	-4.14739690	0.00000000
C	-2.12906802	-6.55071580	0.00000000
H	-0.00632699	-6.15924777	0.00000000
H	-4.29069919	-6.60345689	0.00000000
H	-2.01454344	-7.63188528	0.00000000

Table S11. Cartesian coordinates (in Å), absolute energies, enthalpies, and Gibbs free energies (in kcal mol^{-1}) for the semicarbazone compounds **X-SC1** with **X** = O, S, and Se, computed at COSMO(chloroform)-ZORA-BLYP-D3(BJ)/TZ2P.

O-SC1*E*

E = –4533.79 kcal mol^{-1}

H = –4374.83 kcal mol^{-1}

G = –4410.55 kcal mol^{-1}

N_{imag} = 0

C	-2.63013365	-1.62809596	0.00012852
C	0.89647883	3.21677392	0.00440285
H	0.36381732	5.29820115	0.00476739
C	-3.68721872	-4.22389454	-0.00574752
H	4.07925914	1.98866636	-0.00153383
C	3.27489807	2.71248064	-0.00018324
H	4.58106273	4.41627450	-0.00341408
N	0.27860059	-1.43299958	0.00186092
H	-4.09457673	-5.23224359	-0.00801486
N	1.56610491	0.91036131	0.00328627
H	-0.13734647	2.87574300	0.00657599
C	2.51067017	5.02743045	0.00049443
C	-4.54581947	-3.11911134	-0.00442278
C	1.18226146	4.58186171	0.00335989
H	0.56841004	0.70684905	0.00365704
H	2.19541946	-2.26240283	0.00163032
C	-0.30020690	-2.58943110	0.00018639
H	-5.62351228	-3.26397561	-0.00564361
C	3.54475574	4.08514091	-0.00120960
C	2.36254061	-0.20685606	0.00282019
H	0.29250912	-3.51347082	-0.00034283
C	1.93950593	2.26918455	0.00257284
C	-4.00973857	-1.82155072	-0.00144139
N	1.64174342	-1.40333754	0.00251815
C	-1.75410525	-2.73562918	-0.00124473
H	2.73399070	6.09139508	-0.00036239
O	3.60195479	-0.23447773	0.00255270
H	-1.63641254	-4.89470972	-0.00519978
C	-2.30283650	-4.03429803	-0.00416192
H	-2.21746818	-0.62320532	0.00245798
H	-4.67460778	-0.96067295	-0.00034930

O-SC1*E* "Urea"

E = –4528.51 kcal mol^{-1}

H = –4369.12 kcal mol^{-1}

G = –4407.07 kcal mol^{-1}

N_{imag} = 0

C	1.23260283	-3.93778101	-0.31249457
C	-1.29611970	4.51013996	0.42925599
H	-1.83419806	6.57926916	0.63809100
C	-0.57136189	-6.07948774	-0.44980869
H	1.88165030	3.31535741	0.09321886
C	1.07690832	4.02822852	0.21143762
H	2.37359198	5.73771670	0.25355667
N	0.05824367	-1.28539024	-0.09402464
H	-1.27350669	-6.90842038	-0.50294708
N	-0.62577430	2.21540950	0.18671887
H	-2.32662108	4.16199425	0.47929292
C	0.30772402	6.32931058	0.45694581
C	0.80616112	-6.32147679	-0.49138926
C	-1.01555041	5.87313716	0.51894208
H	-1.62687078	2.05268813	0.22814041
H	-1.58614753	-0.04563631	0.03632701
C	-0.70059967	-2.33072590	-0.15515277
H	1.18033020	-7.33896268	-0.57698543
C	1.34098505	5.39897523	0.30365766
C	0.18709340	1.09885088	0.07460602
H	-1.79610950	-2.24110944	-0.12342199
C	-0.25355956	3.57401599	0.27448397
C	1.70393481	-5.24420153	-0.42224698
N	-0.56452286	-0.07948701	0.01256539
C	-0.15561835	-3.68236139	-0.26957136
H	0.52742891	7.39165429	0.52714445
O	1.41773771	1.11587558	0.03302452
H	-2.12071390	-4.58427000	-0.30696755
C	-1.04878757	-4.77074971	-0.33943334
H	1.92623387	-3.10340140	-0.25872050
H	2.77543566	-5.42916181	-0.45424511

[O-SC1*E*]2 dimer "Stacked"

E = –9083.29 kcal mol^{-1}

H = –8761.82 kcal mol^{-1}

G = –8823.45 kcal mol^{-1}

N_{imag} = 0

H	-1.88639930	-6.28880144	1.42207210
C	-1.50772269	-4.16897569	1.63893858
H	-0.96095469	-4.28223227	2.57254342
C	-2.02869379	-5.30043202	0.99129555
C	-2.72219927	-5.15174430	-0.21330218
H	-3.11738008	-6.02451330	-0.72731753
N	-1.36751415	1.94417818	0.89096807
H	-1.37966230	0.99984746	1.27275765
N	-2.43059142	0.83678032	-0.84428019
H	-2.63038483	0.81705446	-1.84871366
N	-2.22866337	-0.33810701	-0.16310985

C	-0.74058622	4.30416936	1.32155922
H	-1.27300488	4.63400824	0.43920030
C	-0.07227040	5.22026785	2.14027522
H	-0.09638962	6.27561103	1.87745867
C	0.62173180	4.79961855	3.27828351
H	1.14628293	5.51965842	3.90095947
C	0.64804916	3.43679500	3.59827435
H	1.19823191	3.08897669	4.46883841
C	-0.01182501	2.51215603	2.79215771
H	0.02461668	1.45209247	3.03463510
C	-0.71329854	2.93739790	1.64819304
C	-1.88174278	2.03266797	-0.37571463
C	-2.57710973	-1.43547786	-0.74973258
H	-3.02414045	-1.42276863	-1.75057265
C	-2.38212303	-2.74162728	-0.12535391
C	-1.68094382	-2.90185081	1.08891114
H	-1.26858166	-2.02703287	1.58345403
C	-2.89555035	-3.88273730	-0.76925142
H	-3.41745558	-3.77017820	-1.71627834
O	-1.88892627	3.04216162	-1.09962610
C	3.91017618	1.48655749	2.18955960
H	4.26499237	0.59183235	2.69650286
C	4.31313630	2.75325932	2.64141207
H	4.97932514	2.84037454	3.49657961
O	-1.05899540	0.23295836	-3.58492293
N	0.47959776	-0.95660344	-2.29754177
H	1.18057170	-0.77854198	-1.58019935
N	0.45253933	1.35985526	-2.29288848
H	-0.09342052	2.20758047	-2.47287438
N	1.33902783	1.32523135	-1.24513007
C	-0.68732306	-2.70690926	-3.61003033
H	-1.11682866	-1.96365691	-4.26881407
C	-0.95336368	-4.06767161	-3.79388026
H	-1.60059424	-4.37089729	-4.61397284
C	-0.40553058	-5.03205324	-2.94326740
H	-0.62814177	-6.08578062	-3.08975229
C	0.42037144	-4.62397136	-1.88890004
H	0.83995719	-5.35809580	-1.20584402
C	0.69353728	-3.27236006	-1.69211089
H	1.32203422	-2.95758166	-0.86160744
C	0.14429131	-2.30230806	-2.55166795
C	-0.10962839	0.17983754	-2.78554272
C	1.66467682	2.45058744	-0.69993720
H	1.25233365	3.39686115	-1.06902427
C	2.57839732	2.52188898	0.43767137
C	3.05193700	1.36897211	1.09978073
H	2.73040492	0.38981105	0.75661440
C	2.98414139	3.78687902	0.90181444
H	2.60624382	4.68037688	0.41113438

C	3.84694993	3.90092755	1.99364458
H	4.14438512	4.88567787	2.34567218

[O-SC1*E*]2 dimer “Planar”

E = –9078.59 kcal mol^{-1}

H = –8759.16 kcal mol^{-1}

G = –8818.69 kcal mol^{-1}

N_{imag} = 0

H	-7.51685547	-4.53085507	2.24194191
C	-6.28990427	-2.79079186	2.63191677
H	-6.94453153	-2.40701230	3.41128277
C	-6.61360284	-3.98797527	1.97375531
C	-5.76875187	-4.47871928	0.97217699
H	-6.01237774	-5.40537331	0.45765325
N	-1.83511320	1.52535964	2.48667060
H	-2.66502481	0.97193633	2.69385536
N	-1.51487271	-0.21156753	0.98832729
H	-0.95129879	-0.66693705	0.24173158
N	-2.68155179	-0.76878005	1.43846084
C	-0.56921977	3.58770542	3.05116443
H	0.19284114	3.35508611	2.31935567
C	-0.48531470	4.74593383	3.83114785
H	0.36206320	5.41363792	3.69054626
C	-1.46629873	5.05391335	4.77972948
H	-1.38933941	5.95727371	5.37959167
C	-2.55044916	4.18218423	4.94658509
H	-3.32393595	4.40330543	5.67857165
C	-2.64859526	3.02347001	4.17687384
H	-3.49283844	2.34946474	4.31062906
C	-1.65959436	2.71443755	3.22163108
C	-1.04538963	0.97887819	1.51018911
C	-3.05033967	-1.88193028	0.88981623
H	-2.44762972	-2.34586928	0.10056418
C	-4.27429307	-2.57549074	1.28442493
C	-5.13431871	-2.09011195	2.29380071
H	-4.88509213	-1.16419923	2.80439330
C	-4.60890351	-3.77873902	0.63014575
H	-3.95277029	-4.16173235	-0.14910724
O	0.02462649	1.47186158	1.08472103
C	6.27357662	2.80594226	-2.65416295
H	6.92466202	2.42503360	-3.43789087
C	6.59575466	4.00516345	-1.99897939
H	7.49433877	4.55246486	-2.27377112
O	-0.00344449	-1.49255390	-1.05582981
N	1.84215759	-1.53217958	-2.47673021
H	2.66675860	-0.97356504	-2.69104455
N	1.52619746	0.20056379	-0.97261817
H	0.96721512	0.65157228	-0.21994036

N	2.68494200	0.76550120	-1.43350064
C	0.58141785	-3.59956970	-3.03424000
H	-0.17532139	-3.37208521	-2.29533138
C	0.49636296	-4.75671735	-3.81570390
H	-0.34646384	-5.42885441	-3.66900931
C	1.47068447	-5.05824203	-4.77318314
H	1.39296809	-5.96090603	-5.37399475
C	2.54906699	-4.18089968	-4.94781157
H	3.31724961	-4.39688564	-5.68687334
C	2.64830068	-3.02320531	-4.17670450
H	3.48807527	-2.34485321	-4.31646405
C	1.66609287	-2.72075462	-3.21237537
C	1.05896689	-0.99208354	-1.49148701
C	3.05210934	1.88045875	-0.88746205
H	2.45377265	2.34043466	-0.09257732
C	4.26879733	2.58097069	-1.29216399
C	5.12408729	2.09948878	-2.30740366
H	4.87616280	1.17183716	-2.81546238
C	4.60166486	3.78645903	-0.64113229
H	3.94914056	4.16650502	0.14258332
C	5.75545707	4.49218711	-0.99176143
H	5.99792603	5.42040910	-0.47952054

[O-SC1*E*]2 dimer "Chain"

E = –9073.19 kcal mol^{-1}

H = –8752.52 kcal mol^{-1}

G = –8813.69 kcal mol^{-1}

N_{imag} = 0

C	-0.55225053	-3.06065898	-1.73545938
C	-3.09425312	4.58687812	0.73040689
H	-3.13718137	6.73070158	0.55683320
C	-1.63284327	-5.63685005	-1.53424213
H	-1.21401650	2.83110147	2.97211620
C	-1.63060973	3.68190383	2.44620849
H	-0.52639521	5.12884346	3.58741300
N	-1.91448117	-1.04180403	-0.13915857
H	-2.05125068	-6.63729181	-1.45345041
N	-2.95893461	2.17858718	1.00548324
H	-3.80205966	4.42774244	-0.08058894
C	-1.78055889	6.08582207	2.11194633
C	-0.39420950	-5.44180496	-2.15660546
C	-2.71314664	5.88239742	1.08860472
H	-3.83576264	2.13714763	0.49466957
H	-3.71798960	-0.09095849	0.27617172
C	-2.51561065	-2.13602081	-0.47407908
H	0.15408910	-6.29070097	-2.55713930
C	-1.24721804	4.98164818	2.78680811
C	-2.09589530	1.11316463	0.84392403

H	-3.59231917	-2.27161530	-0.30217982
C	-2.54837371	3.48238253	1.40308158
C	0.13726634	-4.15050964	-2.26301343
N	-2.70087075	-0.02806120	0.34747000
C	-1.78744962	-3.24914818	-1.08283245
H	-1.47383384	7.09320497	2.38125656
O	-0.88748643	1.15694093	1.12459212
H	-3.29016728	-4.69987896	-0.51845217
C	-2.33025099	-4.54795141	-1.00845707
H	-0.14366017	-2.05871559	-1.81749905
H	1.09731644	-3.99591177	-2.74825222
C	2.88665809	-4.24211653	0.12895316
C	0.87136241	4.36576060	-0.38375162
H	0.39401996	6.45266830	-0.52153033
C	1.09232559	-6.09667065	1.22009543
H	3.64565610	2.87359444	-1.69609863
C	2.94101928	3.65586278	-1.44599106
H	4.06227083	5.21019798	-2.41335128
N	2.01364100	-1.45845449	0.15421215
H	0.38866725	-6.81445215	1.63557155
N	1.46019678	2.04456835	-0.26439772
H	-0.01924104	4.12884216	0.19233689
C	2.26031241	5.99729948	-1.52220762
C	2.28880227	-6.53807113	0.64508158
C	1.11016494	5.67829020	-0.78714245
H	0.57213510	1.96713542	0.23736275
H	0.53348877	-0.04959678	0.52942986
C	1.28713756	-2.38143418	0.70109251
H	2.52323701	-7.59971540	0.61513039
C	3.16452270	4.97851886	-1.84367099
C	2.18296066	0.86798503	-0.39564450
H	0.31456000	-2.14280478	1.14846205
C	1.78413644	3.33804941	-0.70784501
C	3.18431725	-5.60286900	0.10252230
N	1.48476526	-0.20339410	0.16578460
C	1.68093005	-3.78725535	0.70392552
H	2.44772888	7.02107252	-1.83678474
O	3.29330145	0.76356007	-0.92712700
H	-0.14804869	-4.39338125	1.67915090
C	0.79267925	-4.73351665	1.25143914
H	3.57144695	-3.51801248	-0.30357743
H	4.11525269	-5.94224496	-0.34700665

[O-SC1$_E$]$_4$ tetramer "Stacked"

E = –18187.49 kcal mol^{-1}

H = –17545.30 kcal mol^{-1}

G = –17646.25 kcal mol^{-1}

N_{imag} = 0

H	3.64395541	-1.59911077	-1.96224442
C	3.97916940	0.24244731	-0.87679738
H	4.33770220	-0.25911700	0.01878297
C	3.59213498	-0.51334421	-1.99350168
C	3.14228947	0.13571851	-3.14843866
H	2.84199439	-0.44312057	-4.01886396
N	5.03522150	6.18715675	0.46640816
H	5.05979242	5.17009829	0.40849841
N	3.74210312	5.86887803	-1.42176522
H	3.14744504	6.24631387	-2.18839613
N	3.88012281	4.51933633	-1.23545603
C	5.64176881	8.15986284	1.84379564
H	5.04451632	8.81184812	1.22100260
C	6.34516036	8.66266514	2.94048511
H	6.28530835	9.72563390	3.16022932
C	7.13609390	7.82934905	3.73672580
H	7.68784723	8.23590691	4.58008496
C	7.22689860	6.46864443	3.42142548
H	7.84384540	5.80568695	4.02375348
C	6.53095459	5.95151771	2.32852111
H	6.59978081	4.89171298	2.08849556
C	5.72738409	6.79115026	1.53394614
C	4.30384985	6.76627553	-0.53515095
C	3.40694253	3.75238672	-2.16296893
H	2.94396891	4.17867458	-3.06008938
C	3.47054610	2.29773954	-2.07004940
C	3.92037513	1.63214466	-0.91113231
H	4.23334463	2.21706060	-0.05180721
C	3.07767307	1.53023902	-3.18408304
H	2.72824616	2.03577756	-4.08228318
O	4.14124903	7.99694525	-0.69556326
C	9.69559491	9.08420548	1.82582627
H	10.32140076	8.47840161	2.47659997
C	9.53645340	10.45346789	2.08619375
H	10.04291027	10.91040955	2.93309143
O	6.02646849	5.27377583	-3.85404013
N	7.14547320	4.81552379	-1.86322623
H	7.57824768	5.31730852	-1.08910215
N	6.79205899	6.99075131	-2.55777566
H	6.44658084	7.67271357	-3.26458993
N	7.47978671	7.41070728	-1.45058186
C	6.77152315	2.58501811	-2.88345636
H	6.39393382	3.03444989	-3.79178674
C	6.85397933	1.19726916	-2.75077898
H	6.52979915	0.57046177	-3.57770960
C	7.32071529	0.60956929	-1.57147284
H	7.36815775	-0.47200488	-1.47712949
C	7.70537255	1.43052392	-0.50504907
H	8.06311373	0.99208571	0.42378386
C	7.62809427	2.81853333	-0.62199674

H	7.93249616	3.45472159	0.20756959
C	7.16652683	3.40808414	-1.81423640
C	6.62690922	5.64517855	-2.82046679
C	7.50345951	8.68481368	-1.23000366
H	6.97254247	9.37752996	-1.89273067
C	8.22074427	9.26097419	-0.09775028
C	9.04666352	8.49133779	0.74718716
H	9.15851562	7.42881741	0.55465667
C	8.07262169	10.63658308	0.16741063
H	7.43813213	11.23815999	-0.48059011
C	8.72110877	11.22785940	1.25366376
H	8.59154940	12.28977327	1.44972579
H	-0.07850682	3.48281475	-9.81742288
C	1.14511472	5.20272734	-9.34160026
H	0.81566578	5.77414446	-10.20596615
C	0.63773102	3.91344068	-9.12163189
C	1.05481094	3.18543636	-8.00196096
H	0.66492620	2.18592960	-7.82352875
N	4.51722351	9.36810413	-6.24443802
H	3.84100652	8.89555983	-6.84261120
N	4.72631797	7.20969744	-5.44695090
H	5.18154406	6.51428781	-4.82004175
N	3.82505109	6.81611144	-6.39980497
C	5.70763387	11.50210596	-5.81303663
H	6.37577521	11.01901860	-5.11314434
C	5.82300783	12.86616204	-6.08937071
H	6.59739169	13.44010024	-5.58662823
C	4.94917741	13.50126699	-6.97654064
H	5.04268444	14.56590397	-7.17345353
C	3.93798458	12.75401652	-7.59160833
H	3.24303465	13.23170014	-8.27848241
C	3.80924553	11.39069081	-7.32508944
H	3.02475378	10.81090436	-7.80881300
C	4.69664111	10.75146618	-6.43852721
C	5.13348517	8.52616230	-5.35845475
C	3.42529165	5.58723598	-6.34913875
H	3.78136076	4.91488480	-5.56040820
C	2.48457818	5.03924271	-7.32026697
C	2.05793034	5.76248010	-8.45314147
H	2.43921348	6.76580513	-8.61595704
C	1.97469276	3.74273601	-7.11123666
H	2.29926910	3.17726226	-6.23985601
O	5.98404458	8.86701324	-4.50578538
C	3.35505402	14.46213946	-4.08733118
H	2.70441248	14.99998983	-4.77256693
C	4.40063534	15.13816573	-3.44094014
H	4.55656609	16.19958517	-3.61880838
O	2.02149186	6.84580759	-3.43707309
N	1.47985185	8.61536224	-4.85168832
H	1.70058848	9.60318976	-4.96937037

N	2.91568238	8.91406311	-3.06640339
H	3.39830833	8.54867986	-2.21928870
N	2.99167011	10.23862906	-3.40481043
C	0.05380849	6.74405512	-5.64004603
H	0.35884871	6.12519431	-4.80723231
C	-0.84831764	6.26834036	-6.59407507
H	-1.24105930	5.26014583	-6.48865850
C	-1.23040583	7.05516289	-7.68447678
H	-1.92425927	6.66815010	-8.42595306
C	-0.69150593	8.33962172	-7.82206963
H	-0.96973734	8.96386509	-8.66815381
C	0.21139201	8.82874021	-6.87777374
H	0.62510103	9.83024698	-6.98461826
C	0.58692870	8.03796599	-5.77517594
C	2.11117868	8.04662706	-3.77860426
C	3.83759058	10.96007378	-2.74411678
H	4.47215384	10.51183196	-1.97122478
C	3.99636268	12.38866462	-2.99371246
C	3.15228482	13.10313310	-3.86866664
H	2.35222521	12.57730372	-4.38046057
C	5.03960440	13.07735175	-2.34415381
H	5.69497616	12.53385266	-1.66627439
C	5.24362359	14.44040163	-2.56913416
H	6.05735336	14.95701235	-2.06531602

S-SC1*E*

E = –4454.85 kcal mol^{-1}

H = –4296.32 kcal mol^{-1}

G = –4334.53 kcal mol^{-1}

N_{imag} = 0

C	-2.48608749	-1.71511465	-0.00529797
C	0.66638999	3.12571039	0.00136620
H	-0.14904307	5.11267129	0.00122865
C	-3.44732143	-4.34867199	-0.00159335
H	3.98801208	2.34727884	0.00169093
C	3.09027720	2.95109443	0.00155248
H	4.14950709	4.81767023	0.00141589
N	0.41238363	-1.41387225	0.00005225
H	-3.81811257	-5.37094142	-0.00014715
N	1.61536702	0.93352039	0.00135277
H	-0.31088457	2.64606797	0.00145822
C	2.01537253	5.13916319	0.00137909
C	-4.34506362	-3.27535901	-0.00523333
C	0.76013157	4.51605068	0.00125336
H	0.63193748	0.65440402	-0.00027161
H	2.35074360	-2.18911838	0.00536576

C	-0.12556988	-2.59216731	0.00074014
H	-5.41674100	-3.45934019	-0.00673401
C	3.16834414	4.34809637	0.00152506
C	2.44891272	-0.13826780	0.00411803
H	0.50123985	-3.49299389	0.00299136
C	1.83016437	2.32893518	0.00146884
C	-3.85726236	-1.95877076	-0.00725111
N	1.77406041	-1.34548072	0.00237600
C	-1.57069712	-2.79076129	-0.00156905
H	2.09070913	6.22366426	0.00130192
S	4.14136333	-0.18077232	0.00906196
H	-1.37349731	-4.94436994	0.00310569
C	-2.07114263	-4.10921618	0.00019553
H	-2.11012384	-0.69600336	-0.00668331
H	-4.55336924	-1.12310576	-0.01021925

S-SC1*E* "Urea"
E = –4447.47 kcal mol^{-1}
H = –4289.88 kcal mol^{-1}
G = –4326.70 kcal mol^{-1}
N_{imag} = 0

C	1.13392264	-3.94441336	-0.73134232
C	-1.19841925	4.47677576	-0.21068491
H	-1.71298864	6.54442798	-0.51039282
C	-0.59588689	-6.07636982	-0.15830153
H	1.66030001	3.35126143	1.27209024
C	0.93216559	4.04823012	0.87617065
H	2.07143108	5.78919266	1.40936093
N	0.13008664	-1.28660347	-0.14508939
H	-1.26793061	-6.90182930	0.06470360
N	-0.56244202	2.18723464	0.21592079
H	-2.11225199	4.10134575	-0.66729338
C	0.21304472	6.33390479	0.45088727
C	0.67042080	-6.32715423	-0.69823380
C	-0.96928870	5.85217690	-0.12277166
H	-1.56098520	1.99540227	0.18860535
H	-1.35738712	-0.05250846	0.55547267
C	-0.60442996	-2.32417852	0.09544918
H	0.98706627	-7.34836624	-0.89685254
C	1.15539104	5.42546619	0.94944659
C	0.26573298	1.11279973	-0.02007483
H	-1.61201502	-2.22351883	0.52437702
C	-0.24627947	3.56864878	0.28236251

C	1.53177548	-5.25533579	-0.98278340
N	-0.41266950	-0.07687323	0.16037410
C	-0.14221180	-3.68058567	-0.18660702
H	0.39618257	7.40374607	0.51328282
S	1.87746415	1.20021315	-0.48843615
H	-1.98468334	-4.56850620	0.51633591
C	-0.99972276	-4.76313551	0.09605477
H	1.79790884	-3.11289155	-0.95003215
H	2.51669943	-5.44855607	-1.40199852

[S-SC1$_E$]$_2$ dimer "Stacked"
E = –8929.07 kcal mol^{-1}
H = –8609.82 kcal mol^{-1}
G = –8672.27 kcal mol^{-1}
N_{imag} = 0

H	-2.03955918	-6.15888237	1.28335858
C	-1.68530342	-4.04925794	1.60765903
H	-1.20557322	-4.19823091	2.57244481
C	-2.15617207	-5.15487210	0.88230973
C	-2.76604721	-4.96180488	-0.36102891
H	-3.11960882	-5.81500221	-0.93426030
N	-1.32084350	2.06542184	0.92926046
H	-1.33095403	1.09321605	1.24624740
N	-2.39222276	1.02994604	-0.80366524
H	-2.64698635	1.03025664	-1.79818911
N	-2.21307270	-0.15709164	-0.13929212
C	-0.78107864	4.36909136	1.70808543
H	-1.39854864	4.81396622	0.93915970
C	-0.09449427	5.17161310	2.62525354
H	-0.19251075	6.25257526	2.55448123
C	0.70461135	4.60584460	3.62248290
H	1.24001520	5.24018145	4.32423966
C	0.81670072	3.21238072	3.70262529
H	1.44525638	2.75522214	4.46211727
C	0.14222395	2.40169154	2.79365101
H	0.24708229	1.32006170	2.84470598
C	-0.65929900	2.97323957	1.78705348
C	-1.85866512	2.20198123	-0.30851280
C	-2.60277255	-1.23315854	-0.74540523
H	-3.05029550	-1.18236954	-1.74374394
C	-2.44278333	-2.55682037	-0.15582963
C	-1.82525432	-2.76189544	1.09672156
H	-1.45588668	-1.90604420	1.65437771

C	-2.90819056	-3.67326375	-0.87728566
H	-3.36542520	-3.52388253	-1.85244839
S	-1.90191310	3.57987822	-1.29681752
C	3.94547168	1.54006806	1.97840879
H	4.38360530	0.67191824	2.46575891
C	4.24486821	2.82958493	2.44501246
H	4.91185668	2.96017676	3.29385461
S	-1.42737407	0.12598488	-4.00622439
N	0.42591472	-1.06825824	-2.35576498
H	1.09665587	-0.83154089	-1.62104817
N	0.37579520	1.21601770	-2.41032172
H	-0.13925487	2.07308657	-2.64289724
N	1.26949206	1.21069578	-1.36927434
C	-0.43840736	-3.00548284	-3.66098396
H	-0.79460138	-2.36047409	-4.45316387
C	-0.60165576	-4.39235525	-3.74208299
H	-1.09168468	-4.81377295	-4.61693141
C	-0.14377211	-5.23335635	-2.72410118
H	-0.28398687	-6.30894478	-2.79450990
C	0.48933825	-4.67539957	-1.60674540
H	0.83841194	-5.31253574	-0.79863429
C	0.65472480	-3.29632038	-1.51105338
H	1.12797496	-2.86315223	-0.63249129
C	0.18913745	-2.45004910	-2.53535759
C	-0.15056234	0.03572084	-2.89315199
C	1.59199537	2.36005461	-0.86711084
H	1.15980841	3.28516786	-1.26329683
C	2.50974649	2.48454938	0.25870258
C	3.08671213	1.36548487	0.89677361
H	2.84953283	0.36780908	0.53861430
C	2.81421761	3.77386194	0.73674863
H	2.35634651	4.63896726	0.26325076
C	3.67549947	3.94399993	1.82129199
H	3.89176458	4.94450243	2.18697317

[S-SC1$_E$]$_2$ dimer "Planar" (C$_s$ symmetry enforced)
E = –8919.98 kcal mol^{-1}
H = –8604.11 kcal mol^{-1}
G = –8661.24 kcal mol^{-1}
N$_{imag}$ = 4 (25.4*i*, 24.9*i*, 13.6*i*, 8.3*i* cm^{-1})

H	-8.88142660	2.22281597	0.00000000
C	-7.50384279	0.55377216	0.00000000
H	-8.29109698	-0.19671284	0.00000000

C	-7.83773881	1.91758492	0.00000000
C	-6.82329347	2.88137399	0.00000000
H	-7.07488279	3.93931899	0.00000000
N	-2.44301394	-2.93766370	0.00000000
H	-3.37960984	-2.52613290	0.00000000
N	-1.97715886	-0.70086281	0.00000000
H	-1.30447661	0.08719297	0.00000000
N	-3.32671721	-0.47439767	0.00000000
C	-1.25849775	-5.14021952	0.00000000
H	-0.28501565	-4.66878401	0.00000000
C	-1.37447217	-6.53456818	0.00000000
H	-0.46778566	-7.13552040	0.00000000
C	-2.62576381	-7.15848054	0.00000000
H	-2.70121386	-8.24295206	0.00000000
C	-3.78249118	-6.36747161	0.00000000
H	-4.76555774	-6.83227869	0.00000000
C	-3.68278766	-4.97768070	0.00000000
H	-4.58426841	-4.36747274	0.00000000
C	-2.42007726	-4.34897795	0.00000000
C	-1.48096229	-1.98080063	0.00000000
C	-3.72332150	0.75912031	0.00000000
H	-3.00265751	1.58306992	0.00000000
C	-5.13724185	1.11912093	0.00000000
C	-6.16939268	0.15515692	0.00000000
H	-5.91209479	-0.90016318	0.00000000
C	-5.48353732	2.48610986	0.00000000
H	-4.69469757	3.23573764	0.00000000
S	0.20893775	-2.19410493	0.00000000
C	7.50384279	-0.55377216	0.00000000
H	8.29109698	0.19671284	0.00000000
C	7.83773881	-1.91758492	0.00000000
H	8.88142660	-2.22281597	0.00000000
S	-0.20893775	2.19410493	0.00000000
N	2.44301394	2.93766370	0.00000000
H	3.37960984	2.52613290	0.00000000
N	1.97715886	0.70086281	0.00000000
H	1.30447661	-0.08719297	0.00000000
N	3.32671721	0.47439767	0.00000000
C	1.25849775	5.14021952	0.00000000
H	0.28501565	4.66878401	0.00000000
C	1.37447217	6.53456818	0.00000000
H	0.46778566	7.13552040	0.00000000
C	2.62576381	7.15848054	0.00000000
H	2.70121386	8.24295206	0.00000000

C	3.78249118	6.36747161	0.00000000
H	4.76555774	6.83227869	0.00000000
C	3.68278766	4.97768070	0.00000000
H	4.58426841	4.36747274	0.00000000
C	2.42007726	4.34897795	0.00000000
C	1.48096229	1.98080063	0.00000000
C	3.72332150	-0.75912031	0.00000000
H	3.00265751	-1.58306992	0.00000000
C	5.13724185	-1.11912093	0.00000000
C	6.16939268	-0.15515692	0.00000000
H	5.91209479	0.90016318	0.00000000
C	5.48353732	-2.48610986	0.00000000
H	4.69469757	-3.23573764	0.00000000
C	6.82329347	-2.88137399	0.00000000
H	7.07488279	-3.93931899	0.00000000

[S-SC1$_{E}$]$_2$ dimer "Chain"
E = –8915.25 kcal mol^{-1}
H = –8597.09 kcal mol^{-1}
G = –8658.13 kcal mol^{-1}
N_{imag} = 0

C	-1.29327074	-3.85469596	-0.51136003
C	-2.51457876	4.78323195	0.50495028
H	-2.86607522	6.89979668	0.53428514
C	-0.16952753	-4.88204224	-2.86285412
H	-1.05988958	2.98222857	3.00745438
C	-1.44804303	3.83584189	2.46945540
H	-0.97327703	5.23290107	4.02623824
N	-1.41845543	-0.96114062	-0.32414140
H	0.27513575	-5.27942305	-3.77218799
N	-2.06077689	2.43953437	0.48388262
H	-2.92624630	4.65671835	-0.49436459
C	-1.91557358	6.22394833	2.35658088
C	-0.55231319	-5.74710868	-1.83214442
C	-2.47262272	6.04839399	1.08403971
H	-2.41983474	2.56119090	-0.46050383
H	-1.50994607	0.86579040	-1.28111628
C	-1.05621125	-1.52664339	-1.42976340
H	-0.40487058	-6.81904608	-1.93618430
C	-1.40855118	5.11300279	3.03668406
C	-1.63085991	1.17195567	0.76989960
H	-0.81596395	-0.93335449	-2.32088455
C	-1.99937718	3.66550159	1.18909262

C	-1.11532870	-5.22738835	-0.65822517
N	-1.55243969	0.40239285	-0.36942383
C	-0.90935021	-2.97332160	-1.54332647
H	-1.87897739	7.21155436	2.80936246
S	-1.21908380	0.56439510	2.29581198
H	-0.03981100	-2.82890521	-3.51605546
C	-0.34781375	-3.50485234	-2.72113705
H	-1.71967701	-3.44685574	0.40035120
H	-1.40041356	-5.89756685	0.14851009
C	2.43811217	-4.37008712	-0.28487831
C	1.25417679	4.31957483	0.39555744
H	1.06041292	6.45045396	0.57383668
C	1.62848848	-6.18617852	1.69090601
H	0.34082555	2.47437572	-2.32252468
C	0.49196322	3.33994965	-1.69024551
H	-0.28259354	4.72451096	-3.13708750
N	1.87555835	-1.58168515	0.22736846
H	1.30976232	-6.89019705	2.45622701
N	1.36459096	1.93190532	0.16284330
H	1.66934065	4.19793047	1.39337837
C	0.36153370	5.74601513	-1.34667461
C	2.27634169	-6.64594240	0.53898602
C	0.91017216	5.58784516	-0.06959738
H	1.37331856	1.93904491	1.18019139
H	0.86318892	-0.13962253	1.31072463
C	1.47652133	-2.48310167	1.06471288
H	2.46178249	-7.70909238	0.40425701
C	0.15266119	4.61715299	-2.14603518
C	1.62506948	0.70966413	-0.41698708
H	0.86924283	-2.22007294	1.94037858
C	1.05458724	3.18567730	-0.41231865
C	2.67876401	-5.73135710	-0.44609626
N	1.51010806	-0.29807927	0.51652262
C	1.78501475	-3.89642722	0.87213384
H	0.08919603	6.73394111	-1.70881932
S	2.03824304	0.45189783	-2.02862045
H	0.86815273	-4.46382758	2.74526470
C	1.38307147	-4.82163172	1.85570358
H	2.72632178	-3.65996054	-1.05384858
H	3.17009491	-6.08871057	-1.34818480

[S-SC1*E*]4 tetramer "Stacked"

E = –17876.90 kcal mol^{-1}

H = –17238.40 kcal mol^{-1}

G = –17345.02 kcal mol^{-1}
N_{imag} = 0

H	3.74072621	-1.66654533	-1.31312259
C	4.08427877	0.21058153	-0.29427846
H	4.42368192	-0.26081642	0.62476706
C	3.70260152	-0.58227600	-1.38692517
C	3.27768684	0.02657144	-2.57284378
H	2.98356595	-0.58231294	-3.42461998
N	5.25827181	6.13833212	0.75967270
H	5.23356607	5.11792621	0.68435593
N	3.91330971	5.81375776	-1.05005381
H	3.34849781	6.16654851	-1.84456917
N	4.03013275	4.46889915	-0.81398133
C	6.04854149	7.98030202	2.24905238
H	5.47326506	8.72066637	1.71053845
C	6.83555230	8.34707737	3.34331818
H	6.86008229	9.39120501	3.64417451
C	7.59952154	7.40354668	4.03498590
H	8.21646739	7.70562538	4.87713365
C	7.57328120	6.06648713	3.62081752
H	8.16286235	5.31719826	4.14376080
C	6.79224514	5.68603724	2.53107162
H	6.76642407	4.64429306	2.21596705
C	6.02218923	6.63887259	1.83465841
C	4.50701674	6.72009229	-0.21054683
C	3.57132844	3.67161479	-1.72561433
H	3.14438869	4.06438402	-2.65219076
C	3.61735816	2.22289735	-1.57362417
C	4.04313780	1.59834402	-0.38281410
H	4.34802900	2.21213614	0.45909734
C	3.23164276	1.41892827	-2.66445277
H	2.90694744	1.89504660	-3.58745174
S	4.27231399	8.38007477	-0.49404164
C	10.05835271	8.60063628	1.93088373
H	10.70233331	7.96874934	2.53754242
C	9.93350079	9.96517227	2.23186667
H	10.48412143	10.39158722	3.06704547
S	5.88660449	4.89278785	-4.03977503
N	7.22659072	4.51802024	-1.66641481
H	7.67859100	5.05659544	-0.92271141
N	6.95127333	6.66370319	-2.38028096
H	6.58007112	7.36478029	-3.04779296
N	7.68870299	7.05166114	-1.29231990
C	6.89141237	2.17827091	-2.47145775
H	6.51181203	2.51546254	-3.42607267
C	6.99623241	0.81454828	-2.18768721
H	6.68865835	0.09710011	-2.94398335
C	7.46652723	0.36594131	-0.95076960
H	7.53158715	-0.69819797	-0.73987776

C	7.83840454	1.30357220	0.02001726
H	8.20363378	0.97515504	0.99020547
C	7.73981637	2.66738902	-0.24943381
H	8.03784329	3.39495684	0.50352892
C	7.26611170	3.11812821	-1.49774021
C	6.73491827	5.33424861	-2.63395029
C	7.73076086	8.32013408	-1.03456496
H	7.17071854	9.03577362	-1.64230801
C	8.50441868	8.85259527	0.07991194
C	9.35312832	8.04688339	0.86716802
H	9.44169339	6.98896528	0.64022952
C	8.38844355	10.22286389	0.38648525
H	7.73129963	10.84853410	-0.21412786
C	9.09507690	10.77409944	1.45701242
H	8.99148069	11.83198337	1.68704653
H	-0.00062577	3.92787629	-10.31436186
C	1.22065490	5.62540391	-9.76084097
H	0.93900727	6.21122898	-10.63235011
C	0.68823541	4.34002757	-9.58076898
C	1.04289022	3.59236494	-8.45252563
H	0.63124062	2.59652255	-8.30547574
N	4.40009222	9.67598901	-6.41825784
H	3.75184615	9.16101274	-7.01995635
N	4.65551754	7.53261596	-5.68994154
H	5.06398880	6.82724264	-5.04929892
N	3.79090127	7.15927181	-6.68558920
C	5.41726055	11.93493719	-6.10532897
H	6.14703916	11.55643339	-5.40300654
C	5.39301880	13.28909583	-6.44730235
H	6.12127971	13.95673608	-5.99425842
C	4.44244247	13.79457496	-7.33799763
H	4.42847670	14.85263994	-7.58586708
C	3.49796190	12.92471633	-7.89582183
H	2.74766403	13.29859616	-8.58848506
C	3.51058366	11.57112359	-7.56423454
H	2.78069829	10.89478227	-8.00584696
C	4.47051470	11.06277923	-6.66646469
C	5.03659504	8.84225785	-5.55573233
C	3.36389476	5.93680553	-6.66232426
H	3.65825624	5.25437771	-5.86065435
C	2.46222801	5.41727340	-7.68262129
C	2.09861087	6.16150740	-8.82416246
H	2.50289732	7.16010069	-8.95810162
C	1.92644248	4.12540664	-7.51216370
H	2.19945455	3.54851569	-6.63087258
S	6.17566867	9.24867417	-4.36052996
C	2.82695217	14.54193875	-4.37848505
H	2.13676440	15.05397866	-5.04442540
C	3.86167346	15.25852201	-3.75879872
H	3.96996381	16.32533073	-3.93970298

S	1.81238121	6.47137130	-3.57426562
N	1.27557070	8.65944372	-5.15303996
H	1.49814932	9.65610471	-5.22094512
N	2.62556268	8.98384654	-3.34716819
H	3.15009306	8.63601680	-2.52334658
N	2.64556502	10.31223132	-3.68304965
C	-0.18281200	6.89944472	-6.15811477
H	0.03456253	6.20295336	-5.36013506
C	-1.03660720	6.54104816	-7.20400667
H	-1.47908481	5.54831301	-7.20202864
C	-1.30945009	7.42442206	-8.25167790
H	-1.96643514	7.12739776	-9.06485598
C	-0.71437741	8.69160155	-8.24982947
H	-0.91119525	9.39275107	-9.05740165
C	0.13907337	9.06299597	-7.21250305
H	0.59399723	10.05201405	-7.20836280
C	0.41287098	8.17103217	-6.15646432
C	1.87306721	8.09613974	-4.07150898
C	3.48823708	11.06496822	-3.05020730
H	4.17179019	10.64294327	-2.30874448
C	3.58124734	12.49703059	-3.30454310
C	2.68493533	13.17599890	-4.15602985
H	1.89044177	12.61936034	-4.64334192
C	4.61478513	13.22532831	-2.68297139
H	5.31238609	12.70597294	-2.02900017
C	4.75605302	14.59539576	-2.91147844
H	5.56223455	15.14448510	-2.43051345

Se-SC1*E*

E = –4433.93 kcal mol^{-1}

H = –4275.67 kcal mol^{-1}

G = –4313.90 kcal mol^{-1}

N_{imag} = 0

C	-1.97611294	-4.15881708	0.00862697
C	0.59983035	3.15088094	0.14439457
H	-0.24713059	5.12275641	0.23797217
C	-3.82149183	-2.06064201	-0.08427303
H	3.91177312	2.43398844	-0.25075193
C	3.00934405	3.02228754	-0.14356780
H	4.02625398	4.90814517	-0.27228149
N	0.43010606	-1.39427881	0.02218276
H	-4.54075368	-1.24562750	-0.12048300
N	1.58537564	0.97700070	0.02195952
H	-0.36091235	2.65239528	0.26102509
C	1.90143273	5.18894579	-0.01539255
C	-4.27158487	-3.39054556	-0.06912519
C	0.66662053	4.54298869	0.13146864

H	0.60567741	0.68176055	0.02012402
H	2.38548154	-2.12447817	0.10343150
C	-0.07552285	-2.58720774	0.02981191
H	-5.33724870	-3.60523346	-0.09340680
C	3.06150163	4.42017388	-0.15288180
C	2.43095442	-0.07649884	0.06739531
H	0.57550236	-3.46995243	0.06476963
C	1.77105450	2.37808042	0.01034426
C	-3.34438808	-4.43754863	-0.02268725
N	1.79150101	-1.29374333	0.05991451
C	-1.51345430	-2.82658550	-0.00574000
H	1.95577826	6.27468105	-0.02389628
Se	4.29156084	-0.11384065	0.15498642
H	-2.11132937	-0.74840215	-0.06476554
C	-2.45831986	-1.77766278	-0.05301725
H	-1.25527712	-4.97324263	0.04474341
H	-3.68622188	-5.46977757	-0.01088078

Se-SC1 "Urea"
$\boldsymbol{E}$ = –4426.17 kcal mol^{-1}
$\boldsymbol{H}$ = –4268.87 kcal mol^{-1}
$\boldsymbol{G}$ = –4306.52 kcal mol^{-1}
N_{imag} = 0

C	1.11618325	-3.93744162	-0.75895232
C	-1.18212815	4.47169231	-0.25588714
H	-1.66784444	6.53939779	-0.60574295
C	-0.59025243	-6.07508592	-0.13730363
H	1.60436168	3.34651761	1.35763776
C	0.89972399	4.04531814	0.92345836
H	2.02930238	5.78626039	1.47895235
N	0.12859023	-1.28586361	-0.13516855
H	-1.25286676	-6.90284758	0.10450968
N	-0.57344176	2.18329463	0.22791593
H	-2.07840727	4.09451892	-0.74429263
C	0.21547451	6.32805861	0.43608751
C	0.65934440	-6.32116143	-0.71684562
C	-0.94623313	5.84727900	-0.17833980
H	-1.57325328	1.99136586	0.23875277
H	-1.34984066	-0.05958445	0.59494575
C	-0.59955551	-2.32363010	0.12254828
H	0.97201919	-7.34110973	-0.92781848
C	1.13061815	5.42144837	0.98696087
C	0.24485080	1.11318049	-0.01412275

H	-1.59610644	-2.22438645	0.57683237
C	-0.25609166	3.56758446	0.28766930
C	1.50911028	-5.24673172	-1.02558372
N	-0.40979658	-0.07670651	0.18604915
C	-0.14335565	-3.67857755	-0.17457540
H	0.40391317	7.39744429	0.49043249
Se	2.00527546	1.21540662	-0.58115768
H	-1.96138495	-4.57212345	0.58305128
C	-0.98940992	-4.76347444	0.13223318
H	1.77043074	-3.10329112	-0.99630496
H	2.48077036	-5.43675182	-1.47594141

[Se-SC1$_{E}$]$_2$ dimer "Stacked"
E = –8888.73 kcal mol^{-1}
H = –8570.74 kcal mol^{-1}
G = –8632.87 kcal mol^{-1}
N_{imag} = 0

H	-2.11478178	-6.16704395	1.22497058
C	-1.75582801	-4.06499919	1.58941612
H	-1.29632299	-4.23201818	2.56104285
C	-2.21834241	-5.15629758	0.83753933
C	-2.80315204	-4.94071686	-0.41423262
H	-3.14974996	-5.78296366	-1.00747639
N	-1.29214482	2.06383471	0.95660045
H	-1.29979920	1.08882070	1.26814449
N	-2.37083392	1.05025894	-0.77927896
H	-2.63226015	1.05771777	-1.77378379
N	-2.20747650	-0.14483808	-0.11995889
C	-0.80339998	4.35821275	1.79059312
H	-1.45216485	4.80462920	1.04807629
C	-0.12080983	5.15596006	2.71459323
H	-0.25327120	6.23489720	2.67776241
C	0.71699946	4.58651986	3.67776276
H	1.24845228	5.21716705	4.38589273
C	0.87142957	3.19547136	3.71725194
H	1.52939605	2.73682617	4.45035937
C	0.20131932	2.39001717	2.79998383
H	0.33906292	1.31111931	2.81754055
C	-0.63669741	2.96615435	1.82731116
C	-1.83651826	2.20611577	-0.27135922
C	-2.61324760	-1.20908903	-0.73761324
H	-3.05629017	-1.14078283	-1.73678568
C	-2.47133661	-2.54119531	-0.16437342

C	-1.87885090	-2.76896459	1.09637604
H	-1.51679608	-1.92385897	1.67485791
C	-2.92914021	-3.64360585	-0.91236658
H	-3.36692215	-3.47585702	-1.89345657
Se	-1.88413043	3.71542004	-1.36793398
C	3.97341642	1.59232291	1.94498839
H	4.43162750	0.73644302	2.43550039
C	4.24581863	2.89036143	2.40426572
H	4.91169784	3.03960718	3.25086613
Se	-1.54177805	0.11012762	-4.11025820
N	0.43113683	-1.09859925	-2.34188236
H	1.09862583	-0.85762848	-1.60419666
N	0.37726546	1.18072564	-2.41941876
H	-0.13655057	2.03791238	-2.66155184
N	1.27737055	1.18931484	-1.38030649
C	-0.37932853	-3.05028185	-3.65864465
H	-0.70839403	-2.41242673	-4.46876470
C	-0.53674842	-4.43799981	-3.73337744
H	-0.99378251	-4.86872685	-4.62142133
C	-0.11282201	-5.26826631	-2.69187819
H	-0.24753015	-6.34486678	-2.75770190
C	0.48043183	-4.69920255	-1.55846726
H	0.80325865	-5.32837383	-0.73346991
C	0.63875296	-3.31862801	-1.46927485
H	1.08010106	-2.87579631	-0.57905237
C	0.20547415	-2.48434595	-2.51644017
C	-0.13535160	-0.00110207	-2.88862727
C	1.59715309	2.34889554	-0.89869154
H	1.15749083	3.26465472	-1.30768351
C	2.51443724	2.49708200	0.22382184
C	3.11622322	1.39369736	0.86622738
H	2.90050699	0.38944011	0.51295226
C	2.79207579	3.79545720	0.69452761
H	2.31477531	4.64785240	0.21709068
C	3.65130725	3.98943431	1.77646278
H	3.84694635	4.99600481	2.13695041

[Se-SC1$_{E}$]$_2$ dimer "Planar" (C$_s$ symmetry enforced)

E = –8878.69 kcal mol^{-1}

H = –8564.07 kcal mol^{-1}

G = –8620.84 kcal mol^{-1}

N$_{imag}$ = 5 (27.8*i*, 27.3*i*, 13.5*i*, 12.7*i*, 10.3*i* cm^{-1})

H	-8.92556389	2.19416959	0.00000000
C	-7.56191959	0.51389785	0.00000000
H	-8.35540655	-0.22993661	0.00000000
C	-7.88440488	1.88048662	0.00000000
C	-6.86236544	2.83628084	0.00000000
H	-7.10550929	3.89615614	0.00000000
N	-2.51351939	-3.00999696	0.00000000
H	-3.45009877	-2.59575217	0.00000000
N	-2.04079573	-0.77901964	0.00000000
H	-1.36611491	0.00831896	0.00000000
N	-3.39138032	-0.54620064	0.00000000
C	-1.35158810	-5.22501770	0.00000000
H	-0.37284064	-4.76329911	0.00000000
C	-1.48170172	-6.61794240	0.00000000
H	-0.58136523	-7.22828433	0.00000000
C	-2.73968279	-7.22829019	0.00000000
H	-2.82661707	-8.31190196	0.00000000
C	-3.88822032	-6.42549286	0.00000000
H	-4.87601657	-6.88004219	0.00000000
C	-3.77378804	-5.03680074	0.00000000
H	-4.66859736	-4.41685088	0.00000000
C	-2.50438481	-4.42263534	0.00000000
C	-1.55640436	-2.05633519	0.00000000
C	-3.78096936	0.68947147	0.00000000
H	-3.05512353	1.50883121	0.00000000
C	-5.19131882	1.06032446	0.00000000
C	-6.23089670	0.10433102	0.00000000
H	-5.98190768	-0.95295532	0.00000000
C	-5.52598160	2.43022963	0.00000000
H	-4.73083755	3.17311243	0.00000000
Se	0.30330902	-2.26720249	0.00000000
C	7.56191959	-0.51389785	0.00000000
H	8.35540655	0.22993661	0.00000000
C	7.88440488	-1.88048662	0.00000000
H	8.92556389	-2.19416959	0.00000000
Se	-0.30330902	2.26720249	0.00000000
N	2.51351939	3.00999696	0.00000000
H	3.45009877	2.59575217	0.00000000
N	2.04079573	0.77901964	0.00000000
H	1.36611491	-0.00831896	0.00000000
N	3.39138032	0.54620064	0.00000000
C	1.35158810	5.22501770	0.00000000
H	0.37284064	4.76329911	0.00000000
C	1.48170172	6.61794240	0.00000000

H	0.58136523	7.22828433	0.00000000
C	2.73968279	7.22829019	0.00000000
H	2.82661707	8.31190196	0.00000000
C	3.88822032	6.42549286	0.00000000
H	4.87601657	6.88004219	0.00000000
C	3.77378804	5.03680074	0.00000000
H	4.66859736	4.41685088	0.00000000
C	2.50438481	4.42263534	0.00000000
C	1.55640436	2.05633519	0.00000000
C	3.78096936	-0.68947147	0.00000000
H	3.05512353	-1.50883121	0.00000000
C	5.19131882	-1.06032446	0.00000000
C	6.23089670	-0.10433102	0.00000000
H	5.98190768	0.95295532	0.00000000
C	5.52598160	-2.43022963	0.00000000
H	4.73083755	-3.17311243	0.00000000
C	6.86236544	-2.83628084	0.00000000
H	7.10550929	-3.89615614	0.00000000

[Se-SC1*E*]2 dimer "Chain"
E = –8874.41 kcal mol^{-1}
H = –8556.91 kcal mol^{-1}
G = –8619.62 kcal mol^{-1}
$N_{imag} = 0$

C	-1.44582146	-3.79723272	-0.33800394
C	-2.38950853	4.88499465	0.45569450
H	-2.74098452	7.00171517	0.42167640
C	-0.48514927	-4.92602963	-2.71526287
H	-0.88073370	3.16654540	2.98358859
C	-1.28173259	4.00324466	2.42777631
H	-0.77428997	5.45099781	3.92673735
N	-1.43826958	-0.89871564	-0.21804382
H	-0.10260311	-5.36332108	-3.63445221
N	-1.94637653	2.54084038	0.50518484
H	-2.82388767	4.72483269	-0.52915459
C	-1.75206722	6.38601837	2.24507195
C	-0.84899124	-5.74964091	-1.64434340
C	-2.33540460	6.16900304	0.99086221
H	-2.33746136	2.64168032	-0.43002959
H	-1.53404517	0.89675366	-1.22926735
C	-1.16406929	-1.50457679	-1.32839899
H	-0.74951168	-6.82886603	-1.72766094
C	-1.23055652	5.29821022	2.95129991

C	-1.53514958	1.27753896	0.80634845
H	-0.95361091	-0.94333186	-2.24687180
C	-1.86104984	3.79085185	1.16724256
C	-1.33021104	-5.17937647	-0.45767139
N	-1.52388202	0.46894901	-0.29844925
C	-1.08001459	-2.95788940	-1.41069208
H	-1.70643359	7.38809626	2.66402609
Se	-1.00677869	0.63605871	2.47681732
H	-0.30414173	-2.89561095	-3.42584865
C	-0.60061159	-3.53986216	-2.60094478
H	-1.80914923	-3.34992559	0.58256165
H	-1.60077011	-5.81795979	0.37913636
C	2.30371145	-4.48825573	-0.37293913
C	1.44584711	4.23251289	0.34463797
H	1.32288614	6.36417485	0.57273656
C	1.54250769	-6.24644687	1.67389312
H	0.36393703	2.46870910	-2.36468008
C	0.56684668	3.31805857	-1.72475884
H	-0.22843412	4.75379734	-3.10867029
N	1.86127404	-1.67573303	0.12244287
H	1.24246622	-6.92796795	2.46661281
N	1.48459605	1.85104024	0.06537460
H	1.89818740	4.07802999	1.32173086
C	0.52386133	5.71945278	-1.32956407
C	2.11137525	-6.74475956	0.49641150
C	1.12103931	5.51975634	-0.08059577
H	1.52676323	1.85201178	1.08357604
H	0.98490595	-0.18773646	1.26488890
C	1.48173975	-2.55087669	0.99665085
H	2.25415848	-7.81545961	0.36978572
C	0.24687729	4.61364673	-2.14037783
C	1.64975656	0.62112115	-0.51547130
H	0.93579612	-2.25379690	1.90121350
C	1.17987557	3.12216927	-0.47667889
C	2.49004545	-5.85922053	-0.52398851
N	1.55768618	-0.37692183	0.41774802
C	1.73085453	-3.97598551	0.80986648
H	0.26480267	6.72188537	-1.66001372
Se	1.96595850	0.29479296	-2.31410437
H	0.89838857	-4.48383861	2.73873909
C	1.35217646	-4.87225900	1.82899931
H	2.57331186	-3.79931300	-1.16759252
H	2.92006814	-6.24658019	-1.44480170

[Se-SC1$_E$]$_4$ tetramer "Stacked"

E = –17798.12 kcal mol^{-1}

H = –17161.05 kcal mol^{-1}

G = –17270.97 kcal mol^{-1}

N_{imag} = 0

H	3.76706950	-1.68858510	-1.32952064
C	4.10172240	0.17340912	-0.28075552
H	4.42203134	-0.31150128	0.63808805
C	3.73564609	-0.60328640	-1.39017954
C	3.33496164	0.02243877	-2.57593558
H	3.05367214	-0.57434864	-3.44046889
N	5.30694600	6.10245995	0.82803962
H	5.28385177	5.08128102	0.74734113
N	3.96481910	5.78915984	-0.98009226
H	3.39854106	6.14494452	-1.77347285
N	4.07867167	4.43980532	-0.75152444
C	6.09725859	7.92467947	2.34289037
H	5.51813678	8.67222061	1.81739558
C	6.88654041	8.27698064	3.44012591
H	6.90935416	9.31644631	3.75673678
C	7.65369025	7.32438606	4.11580517
H	8.27209817	7.61493037	4.96096969
C	7.62756617	5.99317545	3.68332479
H	8.21852900	5.23705415	4.19463277
C	6.84381036	5.62763129	2.59042744
H	6.81645607	4.59024008	2.26164095
C	6.07244858	6.59003413	1.90955984
C	4.55523738	6.68186430	-0.13572084
C	3.62821477	3.65344396	-1.67738135
H	3.21568853	4.05808965	-2.60505390
C	3.66679579	2.20346999	-1.54243533
C	4.06897627	1.56244014	-0.35229302
H	4.36091857	2.16421766	0.50280653
C	3.29716227	1.41580918	-2.65064502
H	2.99348667	1.90619126	-3.57319950
Se	4.27598809	8.50109360	-0.45550292
C	10.07852380	8.58006336	1.98961493
H	10.73112516	7.95922869	2.59842739
C	9.92986277	9.94235241	2.28981627
H	10.47059072	10.37780085	3.12679068
Se	5.88231454	4.81333847	-4.14058883
N	7.27301068	4.46134917	-1.60999496
H	7.71826147	5.00154475	-0.86182650
N	7.00913412	6.60040230	-2.33404628
H	6.63912575	7.30185074	-3.00323826
N	7.73903553	6.99358365	-1.23939149
C	6.95540447	2.11299293	-2.39938881
H	6.59329344	2.44041708	-3.36480396
C	7.05542241	0.75223622	-2.10061388

H	6.76194807	0.02727749	-2.85528398
C	7.50422464	0.31644887	-0.85116160
H	7.56597156	-0.74551395	-0.62855090
C	7.86038443	1.26355907	0.11624486
H	8.21016553	0.94493083	1.09528304
C	7.76638538	2.62453403	-0.16880756
H	8.05346601	3.35982920	0.58078831
C	7.31210088	3.06170449	-1.42866636
C	6.79793069	5.27607640	-2.57943470
C	7.76642163	8.26323831	-0.98345660
H	7.20073644	8.97105855	-1.59462565
C	8.52714881	8.80685182	0.13372883
C	9.38594611	8.01461736	0.92365883
H	9.49315942	6.95846681	0.69638602
C	8.38689761	10.17500848	0.43962128
H	7.71984361	10.78834164	-0.16252499
C	9.08061395	10.73761424	1.51239781
H	8.95863803	11.79350818	1.74249846
H	-0.04707464	3.93126443	-10.29843261
C	1.18077233	5.63560730	-9.78240403
H	0.89740663	6.20515873	-10.66405600
C	0.64579105	4.35535770	-9.57551230
C	1.00234080	3.62822155	-8.43435645
H	0.58798429	2.63679793	-8.26667960
N	4.36648908	9.73772635	-6.48037670
H	3.71318548	9.21960217	-7.07579854
N	4.63680525	7.60128980	-5.75134321
H	5.05059693	6.89721686	-5.11111707
N	3.76495689	7.21968863	-6.74146667
C	5.37648477	12.00511186	-6.20195692
H	6.12476929	11.63502296	-5.51385648
C	5.33596529	13.35717830	-6.55011440
H	6.06957552	14.03193439	-6.11674115
C	4.36338819	13.85106374	-7.42346143
H	4.33701755	14.90762438	-7.67672652
C	3.41436993	12.97184451	-7.95831654
H	2.64825693	13.33664104	-8.63829231
C	3.44359899	11.62019129	-7.61995906
H	2.71109113	10.93583297	-8.04438799
C	4.42414006	11.12467657	-6.73807963
C	5.00929648	8.90687461	-5.62826716
C	3.33699967	5.99757643	-6.69919862
H	3.63378286	5.32739990	-5.88850295
C	2.42966088	5.46315590	-7.70574939
C	2.06377029	6.18715087	-8.85947762
H	2.47103687	7.18154441	-9.01419458
C	1.89079792	4.17647651	-7.50779584
H	2.16448740	3.61767650	-6.61528232
Se	6.27321885	9.33396817	-4.32072651
C	2.81531237	14.57997116	-4.42184523

H	2.12354316	15.10545144	-5.07552883
C	3.86683017	15.27886229	-3.81034664
H	3.98655654	16.34537777	-3.98571820
Se	1.74850010	6.36181810	-3.56493850
N	1.22196494	8.69456123	-5.22122308
H	1.44942108	9.69108302	-5.29212781
N	2.56631150	9.01473283	-3.41592745
H	3.09215325	8.66629606	-2.59202770
N	2.59351356	10.34596735	-3.75210028
C	-0.25265884	6.95137852	-6.23415918
H	-0.05070058	6.25286287	-5.43311557
C	-1.10288957	6.60214356	-7.28587773
H	-1.55807136	5.61522315	-7.28578755
C	-1.35711621	7.48807617	-8.33613373
H	-2.01192440	7.19849629	-9.15378530
C	-0.74797454	8.74850679	-8.33047381
H	-0.93150717	9.45177514	-9.13925363
C	0.10188449	9.11034751	-7.28684241
H	0.56687561	10.09457228	-7.27861172
C	0.35820673	8.21487110	-6.22986205
C	1.81262626	8.13853516	-4.13899970
C	3.44903135	11.08766614	-3.12254495
H	4.13456128	10.65470595	-2.38972463
C	3.55674364	12.51919143	-3.37046847
C	2.65863694	13.21440427	-4.20669203
H	1.85076606	12.67103856	-4.68696145
C	4.60760901	13.22956532	-2.75722600
H	5.30714782	12.69570266	-2.11739618
C	4.76349795	14.59895647	-2.97864801
H	5.58275730	15.13466056	-2.50479802
